# Harnessing plant agriculture to mitigate climate change: A framework to evaluate synthetic biology (and other) interventions

**DOI:** 10.1093/plphys/kiaf410

**Published:** 2025-09-26

**Authors:** Claudia E Vickers, Philipp Zerbe

**Affiliations:** Centre for Environment and Society, School of Biology and Environmental Science, ARC Centre of Excellence in Synthetic Biology, and Centre for Agriculture and the Bioeconomy, Queensland University of Technology, Brisbane, QLD 4001, Australia; BioBuilt Solutions, Brisbane, QLD 4075, Australia; Department of Plant Biology, College of Biological Sciences, University of California Davis, Davis, CA 95616, USA

## Abstract

Plant agriculture contributes substantially to global greenhouse gas emissions, yet it also offers powerful opportunities for climate change mitigation. Here, we focus on how to identify and prioritize synthetic biology strategies to reduce emissions and sequester carbon through plant-based interventions. Effective solutions must process large volumes of carbon, be scalable, yield a positive life-cycle balance, and be economically viable, technically feasible, and deployable in field conditions without undue damage to what remains of nature on Earth. Using Fermi estimation, we quantify the per-hectare, annual, and 100-year CO_2_-equivalent (CO_2_e) drawdown potential of emerging synthetic biology strategies—including improved CO_2_ fixation, reduced yield losses, root-deposited biopolymers, engineered nitrogen fixation, and methane reduction—and benchmark them against nonengineered approaches such as biochar, forestation, and fast-growing biomass crops. We used a 100-year horizon to allow for both development and implementation of high-risk but high-impact synthetic biology strategies. We integrate factors such as per-hectare effectiveness, year-on-year sequestration, deployment area, and storage durability. We demonstrate that while per-hectare impacts vary by orders of magnitude (<1 to >30 t CO_2_e/ha/year), deployment scale is the dominant factor determining total impact. Targeted synthetic biology strategies implemented across existing agricultural systems could deliver ∼120 Gt CO_2_e drawdown over a century and contribute to an additional ∼140 Gt CO_2_e drawdown. Decreasing synthetic nitrogen fertilizer use and biochar implementation have the biggest CO_2_e impact potential. Early-stage quantitative evaluation is critical to guide R&D toward climate-relevant solutions and deliver a prioritized portfolio of near- and long-term strategies. A transdisciplinary approach—linking synthetic biology, agronomy, engineering, and social systems—is essential to realize impact. This work offers a framework for evaluating plant agriculture-based climate mitigation strategies and highlights a key role for synthetic biology in mitigation pathways. Regular re-evaluation of strategies should be performed to ensure that they are meaningful for climate change mitigation as other factors evolve.

## Teaser

The scale of plant agriculture can be used to mitigate climate change, and meaningful targets (synthetic biology and comparators) can be selected to increase carbon sequestration or reduce emissions.

## The potential for plant agriculture in mitigating climate change

**Figure 1.**
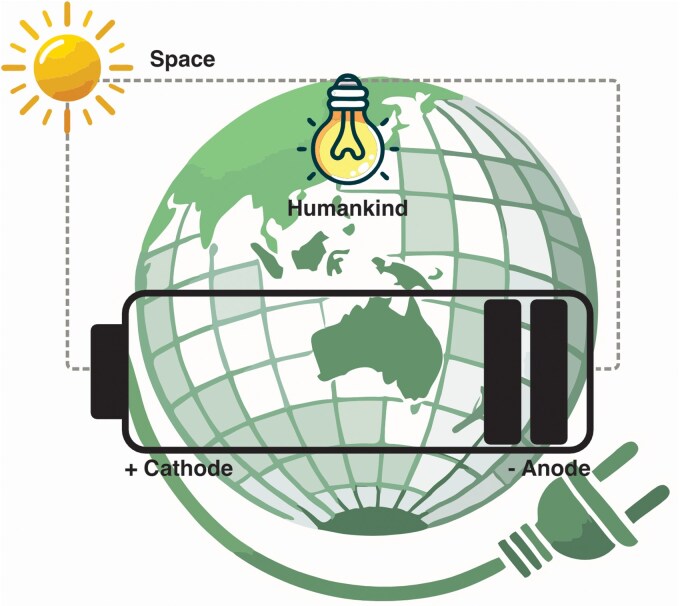
Cartoon representation of the Earth–Space battery concept (Schramski et al. 2015). Energy from sunlight is captured by photosynthesis over millennia. This energy is converted and stored in chemical bonds, turning the planet into a cathode of stored organic chemical energy (biomass and fossil fuels). Humans play a dominant role in dissipating this energy, which eventually radiates as heat toward the chemical equilibrium of deep space (anode). The battery is rapidly discharging without significant replenishment in the discharge timescale. Figure and caption adapted from Schramski et al. (2015) with permission, using Corel Draw.

For ∼3.5 billion years, nature has used photosynthesis to capture solar energy and store it in chemical bonds. The evolution of oxygenic photosynthesis resulted in a dramatic increase in surface carbon cycle fluxes (Lyons et al. 2014) and effectively turned the Earth–Space system into a solar-powered battery (Fig. 1), where Earth operates as the cathode and stores organic chemical energy (Schramski et al. 2015). This battery stores both energy and carbon in biomolecules. Relatively short cycle living biomass forms a dynamic part of the carbon cycle, whereas fossilized biomass (coal, oil, and natural gas—production of which peaked 300 to 400 mYA) forms a long-term carbon store.

In the last few hundred years, industrialization, population growth, and the intensification of agriculture have been enabled by large-scale burning of fossil fuels. The Earth–Space battery, charged over billions of years, is undergoing a rapid discharge as a consequence of this. Biogeochemical cycles have been transformed (Schramski et al. 2015) and Earth's carbon fluxes dramatically altered again. In 2024, the total global CO_2_ emissions derived from fossil fuels were 37.4 Gt (billion tonnes), an increase of 0.8% since 2023, and they will continue to increase (Friedlingstein et al. 2025). At the same time, the ability of ecosystems to absorb CO_2_ is decreasing, due in part to the loss and degradation of soils, native plant communities, and other natural carbon sinks (Ontl and Schulte 2012). A significant rise in average global temperatures is underway (IPCC 2023). Atmospheric and biospheric impacts are already being felt regionally and globally, and more frequent extreme weather events are virtually certain (IPCC 2023). The extent of these impacts will depend on our ability to reduce greenhouse gas (GHG) emissions and increase carbon sequestration within this decade and beyond (IPCC 2023). The task is formidable: to meet the Paris climate stabilization goals, we need to reduce carbon emissions by 10 Gt of CO_2_-equivalents (CO_2_e) per year and remove a further 10 Gt CO_2_e from the atmosphere by 2050 (National Academies of Sciences, Engineering, and Medicine 2019). Faced with this challenge, we must put into action multiple concerted and integrated strategies.

Plant agriculture plays a critical role in global carbon fluxes. Global cropland net primary productivity (NPP) was estimated to be ∼5.25 Pg C (∼19 Gt CO_2_e) for 2011 (Wolf et al. 2015). Roughly half of fixed carbon is lost due to respiration (Amthor et al. 2019), making the total carbon fixed ∼10.5 Pg C (∼38 Gt CO_2_ equivalents) in 2011. However, this may be an underestimation. Recent analyses indicate that photosynthesis fixes ∼157 Pg C (∼576 Gt CO_2_) per annum globally (Lai et al. 2024). Based on a global cropland contribution of 20% of global gross primary productivity (GPP), we estimate that plant agriculture could be fixing ∼31.4 Pg C (∼115.2 Gt CO_2_) per year (Supplementary Table S1)—three times the previous estimate. Leveraging this CO_2_ sequestration capacity is an attractive proposition. Plant agriculture, forestry, and land use also account for 12.5% of global GHG emissions (Ritchie et al. 2020)—another target for intervention. Given the massive scale of plant agriculture, even small innovations that increase sequestration or decrease emissions have the potential to make significant contributions to mitigating climate change.

## Plant synthetic biology has come of age

Genetic engineering essentially started with the building of the first recombinant DNA molecule half a century ago (Jackson et al. 1972). The possibilities of this technology appeared endless, and when more formalized bioengineering approaches emerged a few decades ago as the field of synthetic biology was established, a dramatic Gartner hype cycle (Linden and Fenn 2003)^1^ was triggered. Synthetic biology was seen as having the power to solve almost any problem. However, even in the relatively early days, significant limitations and challenges were identified (Kwok 2010). Today, we can assess the possibilities far more realistically. Major breakthroughs have been achieved in microbial systems, and many challenges have been overcome. However, the physiological and genetic complexity of plants, along with their larger size, longer life-cycles, and lower transformation efficiencies, pose challenges that cannot readily be addressed by adopting approaches established for microbial systems. Nonetheless, in recent years, our ability to engineer both model plant systems and more challenging crop species has made great strides (Jez et al. 2016; Pouvreau et al. 2018; Wurtzel et al. 2019; Patron 2020; Rhee et al. 2025). For instance, utilization of transient leaf-infiltration systems for rapid analysis (Sainsbury and Lomonossoff 2014; Morey and Khakhar 2024) has allowed us to decipher ever more complex metabolic pathways, interactions, and response systems, as well as prototype intricate genetic circuitry (Kitaoka et al. 2015; Owen et al. 2017; Tiedge et al. 2020). We now have access to robust, predictable, and tunable components for genetic toggle switches, biosensors, complex logic gates, gene editing, and spatiotemporal control, as well as toolkits for specific nonmodel organisms such as diazotrophs, inter-kingdom communication components for engineered symbiosis, and much more (de Lange et al. 2018; Pouvreau et al. 2018; Andres et al. 2019; Wright and Nemhauser 2019; Brophy et al. 2022; Liu et al. 2022; Lloyd et al. 2022; Kocaoglan et al. 2023; Vazquez-Vilar et al. 2023; Venkataraman et al. 2023; Zhong et al. 2023; Boo et al. 2024; Borowsky and Bailey-Serres 2024; Kassaw et al. 2025; Kong et al. 2025). Dramatic decreases in the costs of reading and writing DNA have provided broad access to natural and synthetic components and genetic modules for metabolic and genetic engineering. Likewise, advanced plant metabolomics and proteomics tools provide us the ability to sensitively and specifically examine metabolism (Lew et al. 2020; Nakabayashi and Saito 2020; Belt et al. 2025). Along the way, applying these technologies has greatly advanced our understanding of the fundamental mechanisms underlying plant development, reproduction, defense, ecological adaptation (Weng et al. 2021; Yasmin et al. 2024) and inter-organismal interactions (Huang and Osbourn 2019; Weng et al. 2021; Chakraborty et al. 2024; Bouwmeester et al. 2025).

Remarkable progress has been made toward goals that were considered unattainable a few decades ago, such as mitigating the pleiotropic effects of useful genes by employing predictable, tissue-specific expression (Zhong et al. 2023); controlling complex phenotypes such as root and shoot structure to optimize water, nutrient acquisition, and productivity (Khakhar et al. 2018; Brophy et al. 2022; Kocaoglan et al. 2023); engineering the rhizo-microbiome to improve plant resilience (Kozaeva et al. 2024); engineering plants to secrete molecules that positively influence the rhizo-microbiome (Kwon et al. 2024; Shi et al. 2024); and engineering plants for phytoremediation (Medford et al. 2023). These examples highlight the enormous potential of today's synthetic biology capabilities for improving our plant agroecosystem. One day soon we might even be able to observe metabolism in real time using bespoke metabolite-sensing biosensors (Alexandrov and Vickers 2023) in sentinel plants (Lovell-Read et al. 2023) to detect a wide variety of biotic and abiotic conditions, including soil and water contaminants (Bick et al. 2017). These tools and technologies provide us with an unprecedented level of control over crop genetics and the resulting traits.

## How can we identify and prioritize impactful targets?

**Figure 2.**
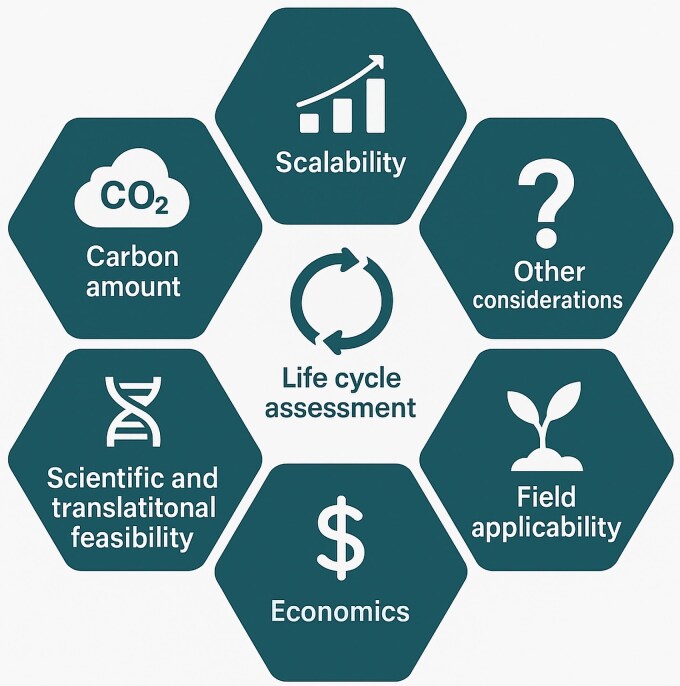
Evaluative criteria for identifying impactful plant synthetic biology interventions for climate change mitigation. These criteria guide the assessment of potential solutions, helping to prioritize those most likely to deliver real-world impact at scale: (i) Amount of carbon—Can the solution influence gigatonne-scale carbon fluxes? (ii) Scalability—Can it be deployed rapidly and widely enough to matter? (iii) Life-cycle assessment—Does it deliver a net climate benefit when all inputs and outputs are considered? (iv) Economics—Is the solution cost-effective and aligned with market demand or policy incentives? (v) Scientific and translational feasibility—Can the biology be engineered within a reasonable time frame? (vi) Field applicability—Will it work reliably in real-world agricultural conditions? (vii) Other considerations—Are broader social, ecological, or policy impacts likely to support or hinder adoption? Figure created using ChatGPT.

Considering the above achievements, we can now ask: how can plant engineering and synthetic biology contribute to reducing GHG emissions and increasing carbon sequestration by plant agriculture? To prioritize interventions that can provide timely and meaningful impact, we must first ask: can this solution scale to a reasonable level of carbon? Fermi calculations—simple, order-of-magnitude estimates based on identifying key factors and using approximate figures—offer a valuable tool for the early evaluation of feasibility. In the following sections, we explore potential strategies to reduce emissions or increase sequestration and demonstrate the use of Fermi analyses to numerically assess their potential. A deeper evaluation can then be performed on strategies that look promising. Key evaluative criteria (Box 1; summarized in Fig. 2) can be used to help prioritize proposed strategies with positive Fermi estimates.

Box 1. Criteria for evaluating climate-positive strategies

1. **Amount of carbon.** Meaningful solutions must be able to process very large amounts of carbon: within 1–2 orders of magnitude of a gigatonne/annum. This can be either a singular process or an ensemble of complementary carbon sequestration or emission reduction solutions.
2. **Scalability.** The intervention must be scalable in a time- and cost-effective manner. Plant agriculture has a significant advantage: it already operates at vast scales and is fully integrated with the food, energy, and bioproduct industry sectors. Even small percentage differences in broadacre crops can have large effects.
3. **Life-Cycle Assessment.** A proper Life-Cycle Assessment (LCA) is required to determine if a given process actually does result in decreased emissions (Skrzypczak et al. 2025)(Chen et al. 2022). For example, U.S. Renewable Fuel Standard (RFS) estimates for 2008 to 2016 showed an unintended increase in the carbon intensity of corn ethanol produced under the RFS due to rising nationwide fertilizer use, increasing water quality degradants, and domestic land use changes during this period (Lark et al. 2022). This resulted in higher emissions than fossil fuel use, defeating the intended purpose. LCA evaluates both direct and indirect emissions and balances them with sequestration. While an international standard for LCA exits (ISO 2020), methodologies vary between practitioners. For carbon sequestration, storage durability is an important LCA consideration (Brunner et al. 2024).
4. **Economics.** Technoeconomic analysis (TEA) is critical to ensure that a proposed solution is economically viable (Kobos et al. 2020; Poddar and Scown 2025). “Lightweight” open-source models are available online (Kobos et al. 2020). Market relationships are also key. There should be a market pull (which can be driven by incentivization) and potential for good economic growth.
5. **Scientific and translational feasibility.** The scientific hurdles to be overcome must be addressable in a reasonable (decadal) time frame. Realistic estimations and prioritizations are critical given the short timeframe remaining to mitigate the worst impacts of climate change.
6. **Field applicability.** Solutions must be deployable and functional under real word field and economic conditions (Khaipho-Burch et al. 2023). Engineered traits commonly behave very differently under controlled and field environments; trait behavior must therefore be assessed across different field conditions and varieties, and on multi-year time spans.
7. **Other considerations**. Knock-on effects and other unknowns can be complex and unpredictable. For example, the early expansion of the biofuels sector in the 2000's also contributed to food price increases, deforestation, and changes in arable land use that displaced smallholder farmers and Indigenous communities (Ziegler 2007; Mitchell 2008; GRAIN 2013; IWGIA 2015), as well as increasing emissions (Lark et al. 2022). This demonstrates the complex and often multi-faceted impacts of individual climate interventions on adjacent sectors and highlights the need for carefully evaluating broader sociopolitical and ecological challenges and impacts. These considerations are further explored in Box 3.

## Reducing emissions

**Figure 3.**
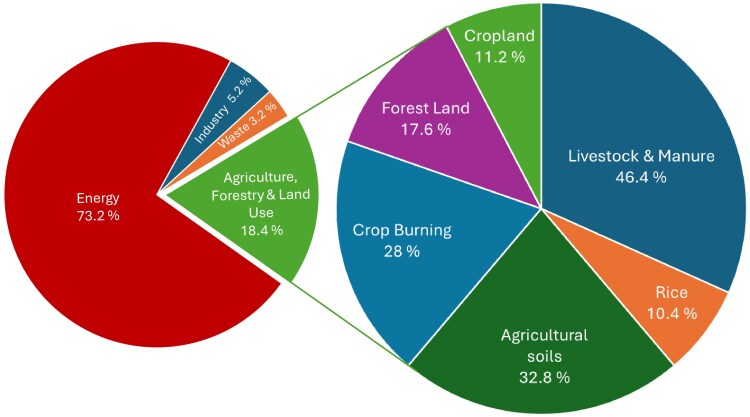
Global greenhouse gas emissions by sector (left) and for agriculture and forestry (right). Data are from 2016, when global greenhouse gas emissions were 49.4 Gt tonnes of CO_2_ equivalents (Jones et al. 2023a, 2023b). The major plant agriculture emissions sources are rice cultivation (methane production from flooded rice paddies), agricultural soils (nitrous oxide from use of synthetic fertilizers), crop burning (burning of agricultural residues), deforestation (net emissions from changes in forestry cover), and cropland (net CO_2_ equivalents after both emissions from land degradation and sequestration from carbon fixation are accounted for) (Ritchie et al. 2020). The source data for this visualization are available at https://ourworldindata.org/ghg-emissions-by-sector.

Identifying emissions sources and scales is the first step to identifying specific areas with high potential for impact. Although estimates of global GHG emissions from agriculture vary depending on how emissions are accounted for and how sectors are identified, the range is relatively small, especially considering inherent uncertainties. Regardless of the accounting model, agriculture is the highest emissions sector after energy (Fig. 3). Estimates for food production alone vary from one-fifth to one-quarter of total global emissions (Poore and Nemecek 2018; IPCC 2019; Rosenzweig et al. 2020; Crippa et al. 2021), depending on whether and how land use, agricultural production, supply chain, and post-retail emissions are accounted for. Models focusing on primary emissions allocate 10% to 20% of global GHG emissions to agriculture, forestry, and land use (Ritchie et al. 2020; Center for Climate and Energy Solutions 2024; Ge et al. 2024; ClimateWatch 2025). However, these percentages also include animal agriculture. Removing livestock and manure leaves ∼12.5% of emissions from plant agriculture and forestry (Ritchie et al. 2020). The major emissions sources are rice (*Oryza sativa*) cultivation (methane production from flooded rice paddies), agricultural soils (nitrous oxide from use of synthetic fertilizers), crop burning (burning of agricultural residues), deforestation (net emissions from changes in forestry cover), and cropland (net CO_2_ equivalents after both emissions from land degradation and sequestration from carbon fixation are accounted for) (Ritchie et al. 2020) (Fig. 3).

CO_2_ is the most impactful GHG by volume, but methane (CH_4_) and nitrous oxide (N_2_O) are also critical targets for agriculture emissions. While smaller in emission volume, these trace gases are particularly important due to their potency. CH_4_ is 25-fold more potent as a GHG than CO_2_ on a 100-year Global Warming Potential (GWP) basis. That means that one molecule of CH_4_ traps 25 times more heat than one molecule of CO_2_ over a 100-year period (IPCC 2023). CH_4_ is the second largest contributor to global warming after CO_2_, despite being relatively short-lived. N_2_O is 270-fold more potent than CO_2_ as a GHG on a 100-year GWP basis (IPCC 2023). It is responsible for ∼10% (∼0.1 °C) of net global warming to date; moreover, it depletes the ozone layer (Organization 2024; Tian et al. 2024). As such, it is a critical target for reducing emissions and limiting global warming. Anthropogenic N_2_O emissions primarily arise from the use of synthetic fertilizers and manure.

### Reducing CH_4_ emissions

Agriculture is the largest source of anthropogenic CH_4_ emissions, with residue burning and rice cultivation being the main sources for plant agriculture (Smith et al. 2021; FAO 2022a, b). Rice cultivation in flooded paddies is the primary source of CH_4_ emissions, due to the anaerobic conditions in the flooded soil favoring microbial methanogenesis. The CH_4_ mitigation potential for rice is ∼10.6 Tg CH_4_ y^−1^ (Linquist et al. 2012; Smith et al. 2021; IPCC 2022a, 2022b). This is equivalent to 0.29 Gt CO_2_e using standardized emissions equivalence conversion (IPCC 2021). How can synthetic biology help deliver on this potential?

Some of the CH_4_ produced in rice paddies is metabolized by methanotrophs in the aerobic parts of the soil. An approach currently being explored is to alter root exudates via genetic engineering to influence the microbial community toward methanotrophy, thereby decreasing net CH_4_ emissions (pers. comm. Subah Soni and Pamela Ronald, University of California, Davis). An alternative strategy involves engineering rice to produce peptide inhibitors of methanogenesis (Shi et al. 2024). The mechanism for this appears to be a reduction of H_2_ available for hydrogenotrophic methanogenesis. An unexplored idea is engineering rice roots to produce direct methanogenesis inhibitors, such as bromoform (which acts as a competitive inhibitor for methyltransferases in the methanogenesis pathway). Bromoform is effective for decreasing CH_4_ emissions from methanogens in ruminant animal guts (McGurrin et al. 2023).

Dryland rice cultivars are grown in unflooded fields, avoiding the anaerobic conditions that favor methanogenesis and decreasing crop CH_4_ emissions by up to 90% (Patel et al. 2010; Luo et al. 2019; Heredia et al. 2022; Zhang et al. 2022). However, they typically suffer an 18% to 40% yield penalty compared to flooded rice cultivars. The practice of alternate wetting and drying (AWD) can be used to decrease CH_4_ emissions in parallel with reducing water use and without affecting yield. However, the outcome depends on many environmental factors including soil conditions, and there are also sociopolitical adoption challenges (Patel et al. 2010; Howell et al. 2015; Carrijo et al. 2017; Leon and Izumi 2022; Bwire et al. 2024; Vicente et al. 2025). Engineering drought resilience in dryland rice might improve yield, allowing the substitution of dry field rice cultivation for flooded paddies and the consequent reduction in CH_4_ emissions (also see “Increasing per hectare yield” below).

Burning of agricultural residues (bagasse, trash, stover, etc.) results in CH_4_ production due to incomplete biomass combustion—albeit the total amounts are small compared to rice cultivation (FAO 2022a, b). A synthetic biology alternative to burning is valorization of biomass as a feedstock for precision fermentation. However, while many breakthroughs have been achieved in lignocellulosic residue conversion, major hurdles are yet to be overcome to achieve cost-effective, efficient lignocellulosic fermentation processes at the scale required to contribute significantly to emissions reduction. Non-synthetic biology options for converting residual biomass into long-term storage carbon—for example, conversion to biochar—can also be considered (see section “Choosing the right tools and determining what not to do”).

### The Haber–Bosch process: CO_2_ and N_2_O

The Haber–Bosch (HB) process, developed and industrialized in the early 1900's, converts atmospheric nitrogen and fossil fuel hydrogen into ammonia fertilizer. This process revolutionized agriculture and food production through the mass production of synthetic nitrogen fertilizer. However, it comes at significant cost: ∼1.8% of global carbon emissions come from this process (Royal Society 2020). N_2_O emissions in agriculture primarily result from the application of fertilizers containing inorganic nitrogen compounds (ammonium, nitrite and nitrate) and associated nitrification and denitrification processes. Therefore, decreasing our dependence on the HB process has high potential for substantially reducing plant agriculture emissions.

There are numerous synthetic biology strategies for engineering alternatives to nitrogen fertilizers and/or improving biological nitrogen fixation. These can be divided into plant-based and microbe-based strategies, including (i) engineering symbiotic nitrogen fixation in nonlegume crops; (ii) inserting nitrogen-fixing machinery directly into crops by engineering nitrogenase activity in chloroplasts or mitochondria; (iii) engineering plant roots to secrete signaling molecules that alter the local microbiome, thereby increasing the local community of diazotrophs; (iv) engineering/improving root secretions that host diazotrophs; (v) engineering nitrogen fixation in crop-associated root microbes or endophytic bacteria; (vi) manipulating nitrogen metabolism in diazotrophs to improve fixation or increase ammonium release; and (vii) engineering improved root/rhizosphere colonization in diazotrophs (Burén et al. 2017; Van Deynze et al. 2018; Wurtzel et al. 2019; Ryu et al. 2020; Haskett et al. 2022; Guo et al. 2023; Johnston et al. 2023; Chakraborty et al. 2024; Martinez-Feria et al. 2024; Venado et al. 2025; Zhao et al. 2025). Some of these approaches, such as direct nitrogen fixation or engineering root nodules in non-nodulating crops (e.g. broadacre cereals) are complex, high risk, high reward efforts with long-term potential that are currently being pursued in academia and industry. However, although the potential of moving nitrogen fixation into nonlegume crops has been recognized for over a century, and significant progress has been made in understanding the molecular mechanisms underlying symbiotic nitrogen fixation, this goal remains a challenge (Rogers and Oldroyd 2014; Huisman and Geurts 2020). Nonetheless, considering the potential impact and recent advances in both our understanding and our plant synthetic biology toolkit, this approach is still considered a viable long-term strategy (Jhu and Oldroyd 2023).

An intriguing recent discovery with high potential for exploitation is the presence of diazotroph-hosting aerial root mucilage in maize (*Zea mays*) accessions from the highlands of Oaxaca (Venado et al. 2025). Fermi analysis suggests that, if this trait was available in US accessions, it could reduce the use of maize fertilizer nitrogen by 10% without significant effect on grain yield (Van Gelder et al. 2023).

## Carbon sequestration/drawdown

Not only emissions reduction, but also an increase in carbon sequestration and storage will be required to achieve global net zero emissions by mid-century (IPCC 2023). To meet the Paris climate stabilization goals, ∼10 Gt of CO_2_e must be removed from the atmosphere per year by 2050, with this number potentially rising to 20 Gt CO_2_ annually by 2100 (National Academies of Sciences, Engineering, and Medicine 2019). These estimates are based on integrated assessment models and assume a global decarbonization trajectory aligned with limiting warming to 1.5 to 2 °C above pre-industrial levels. The 10 Gt target reflects the scale of removal required to offset residual emissions from hard-to-abate sectors such as industrial processes, long-haul transport, and agriculture.

### The importance of durability in carbon sequestration strategies

The durability of carbon storage is a pivotal factor in evaluating the efficacy of CO_2_ drawdown and removal (CDR) strategies. The more durable the carbon storage is, the more impactful it is on carbon fluxes. To meet net-zero targets and achieve long-term climate stabilization under the Paris Agreement, 1,000-year storage duration is desirable (Brunner et al. 2024). Extended durability can be achieved by locking carbon up in degradation-resistant biomolecules or removing carbon completely from biosphere-atmosphere carbon cycles and sequestering it into the geosphere, for example, through conversion to biochar (see section “Choosing the right tools for the job”). Nevertheless, even carbon storage with a 100-year lifespan can contribute meaningfully to net emissions reductions when deployed at scale (National Academies of Sciences, Engineering, and Medicine 2019). LCA is particularly important in these analyses, as it can account for emissions as a result of changes in land use, opportunity costs when considering alternative approaches, and of course, whole-life-cycle emissions including Scope 1 (direct), Scope 2 (indirect, energy source), and Scope 3 (indirect, all other) emissions (WRI & WCBSD 2024). These observations highlight an important consideration: the effectiveness of carbon sequestration strategies depends not only on storage longevity, but also on deployment scale, cost, and integration with broader emissions mitigation efforts.

### Increasing/extending biological sequestration of atmospheric carbon

#### Fixing more carbon and losing less carbon

Nature-based solutions have demonstrated potential in increasing CO_2_ sequestration. Forests act as a major natural sink for CO_2_ sequestration, making reforestation and afforestation efforts a promising avenue for accelerating biological CO_2_ sequestration in the short term (Skrzypczak et al. 2025). Permanent plantings represent a one-off low to high carbon state change for the land; once planted and mature, carbon sequestration decreases dramatically. Carbon in permanent plantations will re-mobilize in a time frame of decades to centuries as trees die and decay. Fast-growing forestry species represent an ongoing carbon sequestration strategy if replanted after harvesting. The impact of forestry plantations depends on the fate and durability of the resulting biomass. Harvested wood can provide long-term storage when embedded in materials and structures—particularly in buildings, where carbon may be retained for centuries. However, forests are also vulnerable to rapid remobilization through increasingly frequent wildfires (Gowda et al. 2018). As noted above, croplands also sequester significant carbon, but most of that carbon is rapidly remobilized. Notwithstanding these considerations, simply growing more biomass at scale will sequester more atmospheric CO_2_ into the biosphere (see section “Fermi analysis in action”), and the more carbon is in the biosphere at any one time, the less there is in the atmosphere. However, can we accelerate biomass accumulation using synthetic biology?

Optimizing CO_2_ assimilation and decreasing carbon loss through engineering of photosynthetic, respiratory, and translocation pathways in crop plants is being explored to increase carbon sequestration, crop yield, and long-term carbon storage in stems and roots and rhizodeposition into the soil (Bar-Even 2018; Kubis and Bar-Even 2019; Yang et al. 2021; Joshi et al. 2023). It should be noted, however, that improved photosynthetic efficiency would only be effective as a carbon sequestration strategy where biomass accumulation is limited by photosynthate production (which is likely the case for any crop that is fertilized, unless the crop has insufficient access to water).

While RuBisCO fixes ∼157 Gt CO_2_ per annum globally (Lai et al. 2024), it is not an efficient enzyme (Bar-Even et al. 2011). Many decades of effort toward improving carbon fixation delivered deep insights into the underlying mechanisms, but various challenges have yet to be overcome to achieve scalable improvements in carbon fixation. Recent enzyme biochemical studies (Prywes et al. 2025) and metabolic design approaches (Bar-Even 2018; Kubis and Bar-Even 2019; Qin et al. 2025) may accelerate progress. Very recently, massively parallel mutational analysis of RuBisCO was used to identify mutations that improve CO_2_ affinity (Prywes et al. 2025), providing targets for engineering to improve carbon fixation efficiency. Alternative, novel, and more efficient carbon fixation pathways could improve carbon sequestration (Wurtzel et al. 2019). AI-guided design and engineering of novel carbon fixation pathways can increase CO_2_ fixation efficiency with improved control over side reactions and regulatory constraints (Qin et al. 2025). Similarly, engineering of natural and new-to-nature pathways has enabled carbon acquisition, for example, in core metabolic intermediates such as acetyl-CoA and glycine (Schulz-Mirbach et al. 2024; Dronsella et al. 2025). Integrating engineered nitrogen and carbon fixation pathways in relevant crop species also has the potential to simultaneously improve photosynthetic efficiency through higher nitrogen availability (e.g. for protein synthesis) and increased nitrogen assimilation with increased availability of carbon resources, thus enhancing plant growth and environmental resilience (Fernie et al. 2020).

One strategy to reduce carbon losses from photorespiration is to reduce the transport of the photorespiration byproduct, glycolate, thereby retaining it for CO_2_ fixation. Engineering of glycolate metabolism in transgenic tobacco plants resulted in a 24% increase in seasonal biomass (South et al. 2019a, 2019b). A similar approach was used in transgenic poplar (Tao et al. 2023). The best transgenic poplar line accumulated 35% to 53% more above-ground biomass than the wild type in short (4 month) trials in controlled environment conditions. Field trials were initiated in 2022 (Living Carbon Team 2022). While promising, many questions have yet to be answered about the long-term performance of lines with decreased photorespiration losses.

Crop respiration results in emission of ∼8 Gt CO_2_ per annum (Garcia et al. 2023; Joshi et al. 2023). Decreasing respiratory carbon loss is a largely underexplored alternative that could complement current photosynthesis-based yield-enhancement strategies (Joshi et al. 2023) and would also deliver sequestration benefits. In this strategy, overall metabolic balance must be considered to avoid yield penalties (Bathe et al. 2023; Sciarresi et al. 2025).

Although the above examples underscore how photosynthetic efficiency has been successfully engineered at the cellular level in several plant species, this may not hold true at the canopy or agroecosystem level due to limitations in, for example, light interception and water limitation (Jardine et al. 2024). Engineered fast-growing photosynthetic microbes such as cyanobacteria, which can be grown in highly intensified systems, are also being discussed as tools to increase both CO_2_ and NO_x_ drawdown (Victoria et al. 2024). However, infrastructure and process costs to deliver the required scale remain a challenge.

#### Sequestering carbon in agricultural soils

It is estimated that agricultural soils could sequester >1 Gt of CO_2_ per annum (Zomer et al. 2017). Optimizing crop traits such as enhanced root biomass and root depth can provide innovative solutions for increasing soil carbon sequestration at agricultural scales (reviewed by Paustian et al. (2016) . Indeed, breeding efforts targeting increased yield in maize over the last 25 years have also led to increases in root mass and soil carbon (Sciarresi et al. 2025). In addition, understanding and engineering of root biochemistry can help improve soil carbon storage, such as the synthesis and exudation of specialized metabolites that mediate defensive and cooperative interactions with soil microorganisms (Bouwmeester et al. 2025). Altering root morphology and function through genetic engineering or breeding can also directly benefit crop drought tolerance, general stress resilience, and nutrient uptake to increase yield and biomass (Zhan and Lynch 2015; Galindo-Castañeda et al. 2018; Jia et al. 2018; Sciarresi et al. 2025). Modern modeling approaches can aid these solutions, for example, by integrating biogeochemical and technology models to estimate the impact of root engineering on carbon sequestration (Faggiani Dias et al. 2025). To establish long-term storage, such approaches must also address soil organic carbon breakdown by microbial communities (Eckardt et al. 2023). One approach to increase durability is to lock carbon up into durable biomolecules (see the following).

### Increasing per-hectare yield

Intensifying production through improving crop performance on the same land area (without, of course, increasing emissions) will also result in an overall decrease in emissions from plant agriculture. The Green Revolution of the 1960s drove major advances in crop breeding and agricultural practices, significantly increasing the productivity of certain grain crops through the introduction of high-yielding varieties and new inputs (especially fertilizers and irrigation). Today, climate change is increasingly impacting agricultural productivity by exacerbating crop losses caused by both abiotic stressors and intensifying pest and pathogen damage. Current approaches to address these problems focus on increasing nutrient uptake and use efficiency, pest and pathogen resistance, and environmental resilience traits to mitigate rising crop losses and enable cultivation on marginal lands (Jez et al. 2016; Yang et al. 2021; Borowsky and Bailey-Serres 2024; Yasmin et al. 2024).

The above targets are all amenable to engineering approaches. For example, novel and exciting applications of synthetic biology to increase yield or to minimize yield loss from pests in rice include the following: (i) Genome editing of rice Baby Boom1(*BBM1*) alongside substitution of Mitosis Instead of Meiosis (*MiMe*) to generate a synthetic, heritable asexual-propagation trait that enables the production of hybrid crops with increased yields (Khanday et al. 2019); (ii) The use of directed evolution on the rice Nucleotide-binding Leucine-rich Repeat (NLR) immune receptor Pik-1 to deliver broader spectrum resistance to the major rice pathogen *Magnaporthe oryzae* (Rim et al. 2024), a generalizable approach that could be used to deliver broad-spectrum resistance in other crops (Rim et al. 2024); (iii) Gene editing to identify an *RBL1* allele, *RBL1*^Δ12^, which confers broad-spectrum disease resistance while preventing the ∼20-fold yield decrease observed in earlier mutations of *RBL1* (Sha et al. 2023); (iv) Very recently, Liu et al. (2025) found that knocking out the rice transcription factor gene *OsWRKY36* resulted in increased biosynthesis of lignin and thickening of the sclerenchyma, conferring broad-spectrum resistance against both insects and diseases without compromising yield. These engineered traits are stackable and can increase per-hectare biomass production, and therefore carbon drawdown. They also help meet food security objectives.

As noted above, microbiome engineering is already being explored for the reduction of methanogenesis in rice paddies. Looking ahead, with advanced synthetic biology and “omics tools available, we can gain a deeper understanding of the phytobiome of rice (Hosseiniyan Khatibi et al. 2024) and other crops. This knowledge could also be used to reprogram phytobiome interactions for disease resistance, increased nutrient uptake, and climate resilience. These approaches will also result in increased per-hectare yields.

### Locking carbon up in durable biomolecules

Given the importance of carbon storage durability, the potential to lock carbon up in highly resistant biopolymers (e.g. cutin, suberin, lignin, and sporopollenin) has drawn significant interest. The growth of engineered plants over-accumulating these polymers and sequestration of polymer-heavy biomass into soils represents a potential approach for increasing soil carbon storage, provided that possible limitations in carbon and/or nutrient availability and uptake can be addressed. For instance, in cases where crops are carbon limited, the production of relatively expensive biopolymer molecules is likely to entail a yield penalty unless it is accompanied by an increase in photosynthetic capacity or a decrease in respiration (Bathe et al. 2023). Where resources other than carbon pose the major constraints to growth, a simultaneous improvement in water or nutrient availability/use is needed, for example, through improvements in root system performance (Bathe et al. 2023). Target tissue mass and crop acreage have a strong impact on this strategy. If the entire US maize crop stored 10% of its root biomass as resistant biopolymers, it would only sequester 13 million tonnes of CO_2_ per year (Bathe et al. 2023). While this contribution is modest as a singular intervention, it could contribute to the many combined solutions that will be required to reach the 10 Gt y^−1^ target. For plants grown specifically for carbon sequestration, the approach would differ—for example, carbon could be stored in shoot tissues as well—and a different cost/benefit analysis would apply (see section “Fermi analysis in action”).

Different biopolymer targets also have different levels of durability. Once in the soil, cutin can be degraded within one year (Mendez-Millan et al. 2010), whereas lignin has a residence time of years to centuries (Zeikus 1981; Zabel and Morrell 2020). Efforts to increase the resistance of lignin are underway in transgenic poplar (Mills 2023). Suberin can be over-produced in transgenic plant tissues (Medford et al. 2023) and remains stabilized within the pool of soil organic carbon for extended periods, with one study showing that it was residual for at least six years (Mendez-Millan et al. 2010). However, suberin can be degraded by both plant and microbial enzymes (Beaulieu et al. 2016; Ursache et al. 2021). The true residence time of suberin in soil is unclear but may decrease if large amounts are available and it becomes attractive as a carbon source for microbes.

Sporopollenin is a complex, variable co-polymer mainly consisting of long-chain fatty acids, phenylpropanoids, phenolics and traces of carotenoids (Guilford et al. 1988). Sporopollenin is a major component of the exine of plant spores and pollen grains and is also found in the cell walls of some green algae (Atkinson et al. 1972; Good and Chapman 1978). It is extremely inert and residual: no enzyme has yet been identified that degrades sporopollenin, and while it is decomposed in the presence of strong oxidizing agents, it is resistant to high temperatures and strong acids and bases (Shaw 1971). Sporopollenin has been found chemically intact in 475 million-year-old sedimentary rock (Wellman et al. 2003), and it is used as a biomarker in the fields of geobiology, palynology and paleoclimatology (Summons and Lincoln 2012). The durability of sporopollenin is likely due to the complexity and heterogeneity of its chemical structure, which provides protection from enzymatic degradation (Li et al. 2019). This durability makes it an attractive biomolecule for long-term carbon storage (Frederick 2020; Mills 2023) and other bio-based applications (Mackenzie et al. 2015; Atalay et al. 2022; Glinkerman et al. 2022; Maruthi and Ramakrishna 2022). At least two groups are currently exploring sporopollenin production as a long-term carbon storage molecule (Mills 2023; Rabin 2023). However, TEA studies aimed at achieving delivery of 1,000+ years of storage at less than $100/ton were not favorable (Mills 2023). This analysis delivered several interesting findings (Mills 2023). In particular, while decay-resistant biopolymers might deliver carbon storage solutions, the fate of the remaining (degradable) biomass must be considered. Other considerations include potential disruption of ecosystems and other environments though deploying such engineered plants (Mills 2023) and the potential for yield penalties in crops (Bathe et al. 2023).

Another extremely durable organic molecule class that has not yet been explored as a long-term carbon storage molecule is hopanoids (hopenes, hopanols, hopanes, and more complex molecules). These pentacyclic triterpenoids are membrane components in bacteria, plants, lichens, and fungi (Volkman 2005). Like sporopollenin, hopanoids are extremely durable and are used as geological biomarkers (Hunt et al. 2002; Summons and Lincoln 2012). While hopanoids are susceptible to oxidation and chemical transformation, the common core hopane skeleton persists. Their environmental durability over geologic timescales is due to their fused, stable structures (Summons and Lincoln 2012). Somewhat ironically—given the context of this discussion—they are well-preserved in petroleum reservoirs (Hunt et al. 2002).

The long-term residence times of suberin, sporopollenin, and hopanoids in agricultural soils are presently unknown, making it difficult to calculate the full benefit of engineering crops to produce these molecules. The incorporation of resistant biomolecules, as well as residual degradable biomass, in large-scale materials that are relatively unfriendly to microbes and other degradative processes could potentially increase the durability of resistant biopolymers, keeping the carbon locked up and out of terrestrial and atmospheric cycles for much longer. After water, concrete is the most widely used substance on Earth, with more than 4 Gt produced per annum (Lehne and Preston 2018). The production of cement, the binder in concrete, is responsible for some ∼8% of GHG emissions globally (Lehne and Preston 2018). Like agriculture, this makes it an impactful target for emissions reduction, because even small differences in emissions make a big difference at scale. The development of construction materials incorporating biomass with high levels of resistant biomolecules could both store carbon drawn down from the atmosphere and decrease the carbon footprint of the construction sector.

As noted, there will inevitably be a metabolic tradeoff between plant growth and the production of resistant biopolymers. In addition, toxicity will almost certainly be a problem at high production levels: for example, hopanoids integrate into lipid membranes and will likely disrupt them at high concentrations; and suberin is highly hydrophobic, potentially forming a water use barrier. How severe this will be in plants, and whether it can be mitigated with clever engineering approaches, can only be determined by experimentation. Engineering toolkits are more established (Vavitsas et al. 2019) and design cycles are much faster in photosynthetic microbes, so using them for prototyping could accelerate research. For example, it might be possible to engineer storage compartments, or to take advantage of photosynthetic microalgae that already produce high levels of lipids and/or have specialized storage organs to address this problem.

## Fermi analysis in action

As outlined above, Fermi analysis can be used to estimate the potential of different approaches for achieving specific goals given a set of quantified assumptions. Oliveira-Filho et al. (2025) and Van Gelder et al. (2023) provide very useful primers for those starting out on using Fermi calculations. A worked example is shown in Box 2 (see Table 1 for parameters and assumptions) and discussed in the following. We ran Fermi analysis on the carbon sequestration and emissions reduction strategies that we have explored above (Supplementary File: Fermi Analysis); a summary comparing the sequestration potential of these strategies is provided in Tables 2, 3, and 4.

Box 2. Fermi estimation for sporopollenin-based carbon storage Enhanced sporopollenin production is a potential solution for the long-term storage of photosynthetically fixed carbon that can be achieved through synthetic biology. Here, we compare carbon storage potentials for sporopollenin in maize in the U.S. to the biomass crop *Miscanthus* × *giganteus* in Europe across root, shoot, and total biomass targets for both crops.

Table 1 provides the assumptions used to run the Fermi analysis. A worked example is shown, followed by a species comparison.

**Fermi Question:** If 10% biomass is converted to sporopollenin, how much photosynthetically fixed carbon (t CO_2_e/ha/yr) could be stored annually in different tissues of maize grown in the US and Miscanthus grown in Europe?

**Step-by-Step Calculation:** Total CO_2_e sequestration per year over 10 mha *Miscanthus* shoots:

1. Sporopollenin produced per hectare = 20 t/ha × 10% = 2.0 t/ha
2. Carbon stored per hectare = 2.0 t/ha × 60% = 1.2 t C/ha
3. CO_2_e stored per hectare per year = 1.2 t C/ha × 3.67 = 4.4 t CO_2_e/ha/year
4. Total CO_2_e sequestration over 10 mha = 4.4 t CO_2_e/ha/year × 10,000,000 ha = 44 Mt CO_2_e/year

**Figure 4.**
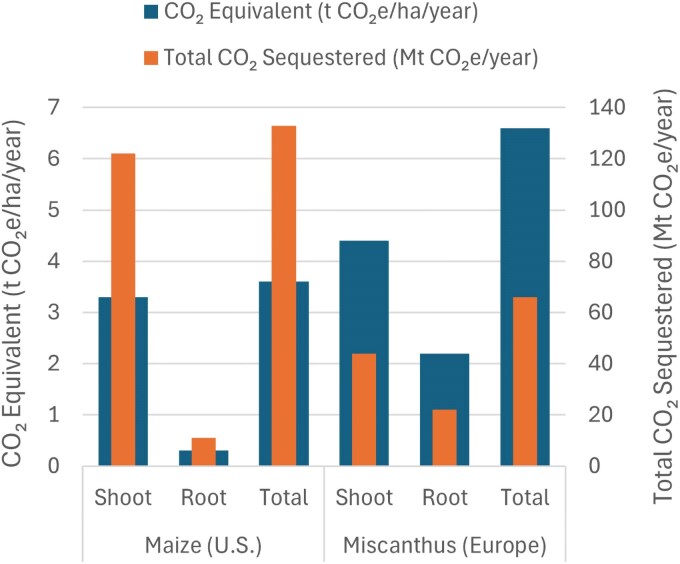
CO_2_-equivalent (t CO_2_e/ha/year) and total CO_2_ sequestered (mt CO_2_e/year) in different tissues of US maize and European *Miscanthus*. These data presume that the total US maize crop, and a potential crop of 10 mha *Miscanthus*, converts 10% of biomass to sporopollenin in different tissues. Supplementary Table S4 shows the raw data.

Once the shoot sporopollenin carbon storage potential is calculated, it is straightforward to calculate the root and total sporopollenin carbon storage potential. Extrapolating using further assumptions noted in Table 1 provides the data presented in Supplementary Table S4 and Fig. 4.

**Table 1.** Assumptions used to run the Fermi analysis

Parameter	Maize (U.S.)	Miscanthus (Europe)
Area (Mha)	37	10
Above-ground biomass (t/ha/yr)	15	20
Root-to-shoot ratio (R:S)	0.09	0.50
Root biomass (t/ha/yr)	1.4	10
Total biomass (t/ha/yr)	16	30
Biopolymer allocation (%)	10	10
Biopolymer mass (t/ha/yr)	1.6	3.0
C content, Sporopollenin (%)	60	60

Numbers for U.S. maize were extracted from Bathe et al. (2023), and calculations are shown in Supplementary Table S2. Numbers, references, and assumption approach for European *Miscanthus* are provided in Supplementary Table S3.

**Table 2.** Summary of potential carbon sequestration strategies using engineered crop and forestry species

ENGINEERED DRAWDOWN STRATEGY	Drawdown profile^a^	Scenario	t CO_2_e/ ha/year	Area (Mha)	CO_2_e (Mt/year)	100-year impact (Gt CO_2_e)	Tech readiness	Benefits	Limitations	Summary
Improved CO_2_ fixation, maize, +15% biomass**^b^**	Sustained	Pilot Total USA crop USA/China**/**Brazil	3.7	0.1 31 100	0.37 128 370	0.04 13 40	Low	Yield-positive; compatible with other engineered traits	Limited impact at early deployment; full benefit requires biomass retention/durability; regulatory hurdles.	Scalable C sequestration with minimal land use change if adopted broadly. Large 100-year impact at scale.
Decreased yield loss in rice, +20% **^b^**	Sustained	Pilot Global	4.95	15 150	74 740	7.4 74	Medium	Improves food production and C efficiency; compatible with existing practices; minimal disruption.	Requires widespread trait deployment; full benefit requires biomass retention/ durability; regulatory hurdles.	Mitigates emissions and boosts food security; strong near-term opportunity if biomass fate ensures C retention. Very high 100-year impact at scale.
Sporopollenin: 10% in maize roots	Sustained	Total USA crop USA/China**/**Brazil	1.1	37 100	41 111	4.1 11	Low	Leverages existing staple crop; integrates with current cropping systems; stored carbon is durable	Experimental; requires genetic engineering for novel traits; untested at scale; may impose metabolic costs.	Potential low-disruption pathway for long-term C storage; promising but early-stage; scalability depends on trait performance and regulatory progress. Moderate 100-year impact, positively compounded due to storage durability.
Sporopollenin: 10% *Miscanthus* shoot, Europe	Sustained	Current area Potential area	4.4	0.002 10	0.88 44	0.01 4.4	Very Low	High biomass productivity; grows on marginal land; low input crop; scalable outside food systems.	Very limited current deployment; speculative trait engineering; land conversion and logistics required.	High per ha C sink with co-benefits; very low TRL; scaling challenges exist—addressable via infrastructure and incentive mechanisms. Moderate 100-year impact; impact depends on fate of biomass.
Engineered forestry trees with reduced photo-respiratory losses +20–40% biomass	Back-loaded	**Poplar (+40%)** Year 1 Year 30–100 **Eucalyptus (+20%)** Year 1 Year 15–100 **Global (+20%)** Year 1 Years 15–100	1.49 9.91 0.89 5.95 0.74 4.95	1.6 20 131	2.4 15.9 18 119 97 649	0.27 6.2 34	Medium Low Very low	Improves carbon fixation efficiency on existing land, with potential for value-add for timber markets. Poplar is currently under field tests	Slow cumulative drawdown due to plantation turnover and age-dependent productivity; limited by current plantation area; trait durability in the field unknown; speculative engineering for nonpoplar tree species	Faster growth and larger deployment areas are key considerations for impact from engineered forestry tree strategies. Impact is highest when compounded over long-time scales. Increasing plantation size would increase impact.

Calculations are made for potential sequestration resulting from crop engineering approaches discussed in the text. Estimates assume that carbon is retained in durable forms and not re-released over time. Assumptions and calculations are provided in Supplementary Tables S5 to S11, along with interpretation comments and notes for each analysis.

^a^“Sustained” strategies are assumed to sequester the same amount annually for 100 years; “front-loaded” strategies (reforestation, afforestation) are modeled as 30 years at full sequestration rate followed by 70 years at 20% of that rate; and “back-loaded” profiles for replacing current forestry plantations with lines engineered for reduced photorespiratory losses account for two key dynamics: (a) progressive replacement of existing plantations with engineered lines, and (b) age-dependent growth over the relevant forestry species rotation cycle.

^b^Durability depends on the fate of the biomass. Short-term biodegradation = months to years. Potential to be converted to longer term storage by converting stover to biochar or stabilizing in other materials.

**Table 3.** Summary of currently available biomass sequestration strategies (not requiring genetic engineering)

DRAWDOWN STRATEGY	Drawdown profile^f^	Scenario	t CO_2_e/ ha/year	Area (Mha)	CO_2_e (Mt/year)	100-year Impact (Gt CO_2_e)	Tech readiness	Benefits	Limitations	Summary
Miscanthus biomass, 10 **m**ha	Sustained	Additional Drawdown Biomass alone	33 37	0.002 10 10	6.6 330 374	0.66 33 37	High	High per-hectare C drawdown; low inputs; valuable co-benefits for soil health and marginal land restoration; does not compete with food production; no engineering required	Durable sequestration depends on fate of biomass; logistics challenges; early-stage adoption, market, and monitoring challenges	Scalable, low-disruption C sink with strong sequestration potential, especially when paired with biomass stabilization (e.g. biochar or valorization to 2nd generation biofuel). Very high 100-year impact at scale. Full benefit depends on biomass fate.
Biochar^a**c**^	Sustained	Wheat stover, Australia Land required for 1 Gt drawdown^de^	2.2 6.2	12 162	26 1,000	2.6 100	Medium/ High	High permanence; soil health improvement; wide feedstock flexibility; positive TEA due to co-benefits.	Requires large-scale biomass supply and distributed pyrolysis infrastructure; logistics-intensive; requires energy supply; potential ecological impacts.	Best near-term C drawdown and durable storage option; already deployable at national scale with multi-sectoral benefits. Possible unintended impacts on ecosystems need to be assessed. Source and cost of energy must be considered.
Reforestation^a^ 150 **m**ha	Front-loaded		11.0	150	1,650	73	High	Biodiversity, watershed protection, soil health; strong international policy support.	Land competition; permanence risk from disturbance; C saturation over time.	One of the most powerful nature-based solutions; effective when protected from re-emission. Very high 100-year impact even considering front-loading.
Afforestation^a^ 100 **m**ha	Front-loaded		9.2	100	920	40	High	Restores degraded lands; long-term biomass sink; biodiversity gains with native species.	Land availability, competition with agriculture, and sociopolitical tradeoffs may limit adoption.	Reliable drawdown method when deployed with ecological safeguards and permanence monitoring; effective when protected from re-emission. Very high 100-year impact even considering front-loading.

Includes currently-available strategies that could be scaled for impact: reforestation, afforestation, biochar, *Miscanthus* biomass. Estimates assume that carbon is retained in durable forms and not re-released over time. Assumptions and calculations are provided in Supplementary Tables S1 (Miscanthus) and Supplementary S12 to S15, along with interpretation comments and notes for each analysis.

^a^Potential for relatively long-term durability (100's of years), but vulnerable to re-emission (e.g. fire). ^b^Durability depends on the fate of the biomass. Short-term biodegradation = months to years. Potential to be converted to longer term storage by converting stover to biochar or stabilizing in other materials. ^c^Biochar calculations performed based on *sustainable* harvest assumptions—i.e. the portion of crop residues or biomass that can be removed without degrading soil organic carbon, nutrient balance, or long-term productivity (Verheijen et al. 2010; Crawford et al. 2016; Cifuentes García et al. 2024). The average was calculated based on both high-biomass and low-biomass crops. ^d^Average CO_2_e/ha /year based on both low-biomass crops such as cereals and high-biomass crops such as switchgrass, Miscanthus, etc. ^e^Fermi analysis was aimed at calculating the area required to deliver 1 Gt CO_2_e impact. ^f^“Sustained” strategies are assumed to sequester the same amount annually for 100 years, “front-loaded” strategies (reforestation, afforestation) are modeled as 30 years at full sequestration rate followed by 70 years at 20% of that rate; and “back-loaded” profiles for replacing current forestry plantations with lines engineered for reduced photorespiratory losses account for two key dynamics: (i) progressive replacement of existing plantations with engineered lines and (ii) age-dependent growth over the relevant forestry species rotation cycle. Estimates assume that carbon is retained in durable forms and not re-released over time.

**Table 4.** Summary of CO_2_e impacts from reducing agricultural emissions: CH_4_, CO_2_, and N_2_O

EMISSIONS REDUCTION STRATEGY	Reduction Profile^b^	Scenario	t CO_2_e/ ha/ year	Area (Mha)	CO_2_e (Mt/year)	100-year Impact (Gt CO_2_e)	Tech Readiness	Benefits	Limitations	Summary
Decreasing global rice methane emissions	Sustained	30% reduction in emissions	0.54	160	86	8.6	Variable	High short-term climate impact. Technically feasible: proven mitigation strategies already exist. Bioengineering strategies in development. Co-benefits: Reducing methane can also improve air quality (via reduced ozone formation) and enhance agricultural sustainability	Some mitigation practices (e.g. alternate wetting/drying) can elevate N_2_O emissions if not properly managed. Some engineering technologies remain a long way from reality	One of the most effective near-term strategies to impact climate. Methane mitigation is vital for short-term temperature control and stabilization trajectories
Converting current agricultural residue burning into generating biochar^a^	Sustained	Complete transition to biochar for ∼500 Mt dry biomass			308	31	High	Significantly reduces CH₄ and black carbon emissions from open burning; enables long-term, stable carbon sequestration in soils; Enhances soil health, water retention, and nutrient cycling; Compatible with the circular bioeconomy and distributed rural deployment; reduces air pollution and associated health impacts	Requires investment in pyrolysis infrastructure and logistics; biochar properties and soil impacts can be variable and context-specific; life-cycle emissions from transport and processing must be monitored. Feedstock competition may emerge as valorization of crop residues gains popularity	Addresses an existing, avoidable emissions source while creating a long-lived carbon sink and improving soil health. However, its climate benefit scales with infrastructure, sustainable residue management^a^, and coordinated deployment strategies. When integrated into broader agricultural and waste management systems, it offers a compelling win–win for climate and productivity.
Alternatives to the Haber-Bosch Process	Sustained	30% reduction in HB dependency			1,300	130	Variable	Dual benefit: Reductions stem from both manufacturing (fossil hydrogen) and downstream emissions (e.g. N_2_O). Some strategies are being commercialized now; others remain long-term goals	Deployment, regulatory acceptance, and ecological safety are all challenges.	Substantial long-term potential: Fertilizer-related emissions exceed 4 Gt CO_2_e/year. Replacing Haber–Bosch with biological alternatives could meaningfully reduce this.

Average reduction scenarios are based on generic approaches, since there are numerous different options. These calculations apply to both synthetic biology and nonsynthetic biology interventions. Reduction profiles are modeled as sustained annually for all these strategies. Assumptions and calculations are provided in Supplementary S16–S18, along with interpretation comments and notes for each analysis.

^a^By default this is sustainable harvesting, since this biomass would otherwise be burned. As per the table footnotes for Table 3: biochar calculations performed based on *sustainable* harvest assumptions—i.e. the portion of crop residues or biomass that can be removed without degrading soil organic carbon, nutrient balance, or long-term productivity (Verheijen et al. 2010; Crawford et al. 2016; Cifuentes García et al. 2024). The average was calculated based on both high-biomass and low-biomass crops. ^b^“Sustained” strategies are assumed to reduce the same amount of emissions annually for 100 years.

### Sporopollenin as a durable carbon storage molecule in maize and *Miscanthus*

Sporopollenin accumulation in maize roots has been proposed, since it shows potential as a mechanism to increase soil carbon durability and would be relatively straightforward to integrate into existing crop systems (Bathe et al. 2023). It would also be interesting to explore the potential for carbon drawdown in a biomass crop for increased scale. The grass *Miscanthus* × *giganteus* is currently being developed as a biomass crop due to its exceptionally high productivity (Zub et al. 2011; Dohleman et al. 2012; Lewandowski et al. 2016). Its low input requirements and perennial nature—with annual regrowth from its extensive rhizomatous root system—further enhance its scalability and sustainability. The use of *Miscanthus* could result in minimal disruption to food systems, as it can be grown on marginal land. Targeting sporopollenin only to above-ground biomass would be an ideal strategy in *Miscanthus* to allow annual harvesting and minimal disruption to rhizomes.

A worked example of a Fermi analysis and comparison between maize (grown in the US) and the biomass crop *Miscanthus* × *giganteus* (grown in Europe) for sporopollenin storage in different tissues is shown in Box 2. The analysis demonstrates a clear advantage of *Miscanthus* for carbon storage per hectare per year, since the biomass per hectare is much greater than maize. This is particularly striking when considering preferred tissues for sporopollenin accumulation: 15-fold more carbon per hectare can be stored in *Miscanthus* shoots than in maize roots. However, the analysis changes when total crop area is considered, since the current crop area for maize is 3.7-fold higher in the United States than the potential crop area used in this analysis for *Miscanthus* in Europe. Thus, the total CO_2_ sequestered (Mt CO_2_e/year) is 2.8-fold higher for US maize than for European *Miscanthus*. However, when target tissue is considered, *Miscanthus* again has a more favorable outcome: 4-fold fold more total CO_2_e/year can be sequestered in *Miscanthus* shoots than in maize roots per annum and on almost four times less land.

It should be noted that while the potential area for *Miscanthus* planting in Europe is estimated to be around 17.5 mha (Lewandowski et al. 2016)—almost twice the 10 mha used for the purposes of this analysis—currently only 0.02 mha is planted (Lewandowski et al. 2016). The total CO_2_e sequestration potential for sporopollenin across the current crop area of *Miscanthus* in Europe is only ∼0.088 Mt CO_2_e/year (Table 2). This underscores the importance of scaling deployment if *Miscanthus* is to contribute meaningfully to carbon drawdown efforts. In addition, when assessing *Miscanthus* as a carbon storage target, use cases for the resulting biomass need to be considered. While maize roots can stay in the ground, *Miscanthus* shoots are currently primarily used as a feedstock for biofuel production, particularly cellulosic ethanol. Sporopollenin accumulation would interfere with this use, and its application as a biofuel feedstock would be counterproductive to the carbon sequestration objective (although some impact would still be achieved from converting from fossil carbon to recently fixed carbon. Such impacts are important for emissions from hard-to-abate sectors such as aviation). Other applications that can benefit from enhanced carbon durability would need to be established, e.g. mulching (will also increase soil carbon), materials (e.g. paper/cardboard, construction materials), and so on. Potential co-benefits (e.g. increased farmer income, soil improvement) and environmental tradeoffs (e.g. impacts of land use change, transport and processing emissions, progressive nutrient removal) should also be considered (Ng et al. 2010; Zub et al. 2011; Dohleman et al. 2012; McCalmont et al. 2017). Accurate modeling and life-cycle assessments are critical for understanding and optimizing these proposed processes (Zhang et al. 2011).

### Comparing carbon management strategies

Similar Fermi analyses can be applied to all the carbon sequestration approaches examined here (see Supplementary File: Fermi Analysis for details). In addition to potential synthetic biology strategies currently being explored, nonsynbio strategies currently available (reforestation, afforestation, and biochar) are included for comparative purposes. For each strategy, an Assumptions & Calculations table and an Interpretation & Notes section are provided (Supplementary File: Fermi Analysis). These analyses are summarized for direct comparison in Tables 2 and 3. An estimation of 100-year drawdown impact is included for each strategy. For the purposes of this exercise, estimates assume that carbon is retained in durable forms and is not released over time, although for many examples, this is not a reasonable assumption.

Before exploring the data, it is important to emphasize again that Fermi analyses are run using a set of *assumptions.* Changes in these assumptions can dramatically affect outcomes, in particular (in these cases), land area, indirect effects, technical feasibility, and so on. Some of these analyses have been performed using a set percentage of current crop area; some assume a significant increase in current crop area; and some use direct calculations of land area extracted from publications. Comparative examples are shown to demonstrate the effect of modifying assumptions. Also, these drawdown estimates presume stable land use, carbon durability, and idealized implementation, which are neither realistic nor accurate in most cases. Fermi analysis identifies strategies that deserve further investigation to present a fuller picture of the intervention being examined, as well as those that can be discarded early. Please keep this in mind as the following interpretations are presented.

#### Carbon sequestration strategies

Firstly, it is interesting to note that the per-hectare annual sequestration rates for almost all strategies are within one, and sometimes just over one, order of magnitude (Tables 2, 3). A notable outlier is *Miscanthus*, with a remarkable potential of 37 t CO_2_e/year, highlighting why it is being pursued as a biomass crop (Lewandowski et al. 2016). The additional biomass from expanded *Miscanthus* cultivation delivers an impressive 330 Mt CO_2_e/year drawdown with a relatively small (10 mha) deployment area (Supplementary Table S5).

As previous analyses have shown (Pan et al. 2011; Griscom et al. 2017; Bastin et al. 2019), reforestation and afforestation lead in total drawdown potential per annum (Tables 2, 3). However, enormous land areas are required to deliver on this potential: 150 mha for 1.7 Gt CO_2_e/year and 100 mha for almost 1 Gt CO_2_e/year. Land availability and use competition are challenges. As noted, forests are also vulnerable to re-release of carbon through fires and may not have the durability of some other approaches.

We also examined biochar, which has been proposed as an effective near-term strategy for durable, scalable carbon sequestration and storage, with numerous co-benefits (Sohi et al. 2010; Verheijen et al. 2010; Woolf et al. 2010; Barlow et al. 2024; Lehmann and Joseph 2024; Sanei et al. 2024; Schmidt et al. 2024), as discussed further in the following. Approximately 162 mha cropland would be required to deliver 1 Gt CO_2_e/year durable carbon drawdown based on average sustainable residue harvesting (the sustainable residue harvest is the portion of crop residues that can be removed without degrading soil organic carbon, nutrient balance, or long-term productivity; Verheijen et al. 2010; Crawford et al. 2016; Cifuentes García et al. 2024). This is a very substantial amount of land. The entire Australian wheat (*Triticum aestivum*) crop is only 12 mha and would deliver only 0.026 Gt CO_2_e/year drawdown in biochar (a value that is also reflective of the relatively small amount of biomass in wheat stover per hectare). To determine cost-effectiveness, logistical challenges for accessing crop residues also need to be considered, among many other things (see further commentary under the section “Choosing the right tools”).

The total annual CO_2_e sequestration impacts from reforestation, afforestation, and biochar are due primarily to the high land area that could be used for deployment. All these strategies could, in theory, meet or exceed gigatonne annual CO_2_e sequestration. Biochar is the most durable. All three strategies also deliver substantially better near-term annual carbon sequestration—by 1 to 2 orders of magnitude—than any of the engineering strategies examined.

The effect of scaling deployment area can also be clearly seen for engineering strategies. For example, improving CO_2_ fixation in maize at small scale (0.3% total US maize cultivation area) delivers a modest drawdown potential. However, when applied at the scale of the entire U.S. field maize crop, the strategy delivers 128 Mt CO_2_e/year sequestration. When the three largest maize producers (USA, China, and Brazil) are considered, CO_2_e sequestered/year becomes 370 Mt. However, this impact relies on the assumption that carbon fixation is the primary limiting factor, which may not be the case.

A similar, and even more impressive impact is seen when scaling increased rice biomass yields as a result of decreasing losses from pests and pathogens. A 15 mha deployment area (e.g. Bangladesh's annual rice planting) delivers 74 Mt CO_2_e/year sequestration, whereas the current 150 mha global rice paddy would deliver three-quarters of a gigatonne of CO_2_e/year sequestration.

These analyses demonstrate that even modest per-hectare increases in sequestration can yield massive benefits when scaled over large areas. A co-benefit of these strategies is their integration with food security priorities, especially for important staple crops like rice and maize. If residual biomass is processed into durable carbon storage or embedded in long-lived materials, these strategies could scale well as net-negative pathways.

Another interesting observation is what happens when a 100-year timeframe is considered (100-year Impact column; Tables 2, 3). For this analysis, we allocated drawdown strategies as (i) *sustained*: assumed to sequester the same amount annually; (ii) *front-loaded*: reforestation, afforestation: modeled as 30 years at full sequestration rate followed by 70 years at 20% of that rate; or (iii) *back-loaded:* replacing current forestry plantations with lines engineered for reduced photorespiratory losses. The latter analysis accounts for two key dynamics: (a) progressive replacement of existing plantations with engineered lines, and (b) age-dependent growth over the relevant forestry species rotation cycle.

Under the 100-year scenario, increased biomass yield in rice matched or overtook forestry approaches for total sequestration, delivering a remarkable 74 Gt CO_2_e potential impact (Table 2). Miscanthus biomass was half this, and similar to afforestation, but would require just one-tenth of the land area. The engineered poplar strategy requires a timeline of 100 years to deliver scaled impact due to the replacement and growth dynamics but would deliver and additional ∼0.27 Gt carbon sequestration over conventional poplar over that time. While the impact is relatively modest, it is scalable and demonstrates the potential of synthetic biology to improve drawdown efficiency in managed forestry systems.

The assumptions behind the poplar model (in particular, that a 40% biomass increase would be sustained in field conditions and over the life of the tree; see Supplementary File) are optimistic. How would more conservative assumptions play out for a fast-growing tree species planted over a larger area? Parsing for high biomass accumulation and large acreage identified Eucalyptus as the most widely planted forestry tree, with forests comprised primarily of just two species, *Eucalyptus grandis* and *E. globulus*, covering 20 mha in the tropics and sub-tropics (FAO 2020). These species have a 15-year harvest cycle (faster than poplar's 30 years) and higher per-hectare carbon sequestration. Assuming a more conservative 20% biomass gain, replacing eucalyptus forests with fast-growing high biomass engineered lines could deliver 6.22 Gt CO_2_e additional drawdown over 100 years (Table 2). Scaling to the global forestry plantation with adjusted assumptions results in a potential 100-year impact of 34 Gt.

If we were to scale sporopollenin production in maize roots to the three largest maize producers (USA, China, and Brazil) there is a 100-year CO_2_e storage potential of 11 Gt (Table 2). However, soil carbon storage capacity for nondegradable carbon would likely saturate relatively early in the 100-year period.

#### Emissions reduction strategies

Table 4 provides a summary of CO_2_e impacts from reducing agricultural emissions of CH_4_, CO_2_ and N_2_O. The average reduction scenarios were modeled based on generic percentages, since there are numerous different options (both bioengineering and nonbioengineering interventions) that could be used to decrease emissions. Reduction profiles are modeled as sustained annually for all the strategies.

Reducing global CH_4_ emissions by 30% could yield a climate mitigation benefit of ∼81 Gt CO_2_e over 100 years (assuming sustained reductions from a 10.6 Tg CH₄/year baseline from rice alone; Table 4). Methane has a 20-year GWP ∼84 to 86 times that of CO_2_ (IPCC 2021), so reductions deliver rapid climate benefits with a high short-term climate impact. This would make it one of the most effective near-term strategies for bending the climate curve before 2040. While it does not represent carbon storage, CH_4_ mitigation is vital for short-term temperature control and stabilization trajectories, especially when coupled with long-term CO_2_ drawdown strategies.

Replacing open burning of ∼500 Mt/year of agricultural residues with biochar production could reduce emissions by ∼308 Mt CO_2_e/year, with a cumulative climate benefit of ∼31 Gt CO_2_e over 100 years—about one-third of the emissions reductions that we modeled to achieve a 1 Gt per annum sequestration benefit. This is not a synthetic biology solution, but is a high-potential, near-term carbon mitigation strategy that provides both immediate emissions reductions and durable carbon sequestration.

Decreasing our reliance on the HB process is the single most impactful opportunity available to us: a 30% reduction in our dependency would deliver a 100-year Impact of 130 Gt CO_2_e (Table 4). Unlike CH_4_ mitigation, which is short-term, HB alternatives can lead to sustained reductions in both CO_2_ and N_2_O emissions, especially as synthetic biology strategies scale in major cereal crop systems. While deployment barriers are real—including technical, regulatory, and adoption challenges—even partial substitution could yield gigatonne-scale emissions reductions annually.

## Choosing the right tools and determining what not to do

History has taught us that there is almost always more than one solution to a given problem. In the context of climate change mitigation, synthetic biology will neither be the only nor always the best solution. Almost invariably, a combination of strategies is needed to deliver the required impact. Comparative Fermi analysis, as we have demonstrated above for accelerated carbon sequestration, can help identify promising strategies. However, prioritization and selection require a more detailed investigation than Fermi analysis, including exploring technical feasibility, reasonable estimation of technology development timelines, incorporation of LCA, and so on (see Box 1).

Sometimes, synthetic biology is clearly not the right choice. For example, the accumulation of sporopollenin in *Miscanthus* shoots, as explored above, is probably not a useful approach unless it contributes to long-term carbon durability. The current use case for *Miscanthus*, i.e. 2nd generation biofuels, would defeat the purpose of sporopollenin production. The value of increased carbon durability in the form of sporopollenin would depend on the end use scenario.

Substantial decreases in N_2_O emissions can be achieved through altered management practices, including fertilizer management, applying arbuscular mycorrhizal fungi, integrated nutrient management, cover cropping, and more (Millar et al. 2010; Hassan et al. 2022; Du et al. 2024). Since these practices can be implemented immediately, they can potentially have much greater effects than genetic engineering approaches in the short term, provided that they are economically sustainable for farmers and growers.

In some cases, breeding to achieve desirable traits will be faster, require fewer regulatory steps, and be more publicly accepted by consumers than engineering those traits. Introgression of diazotroph-hosting aerial roots (Venado et al. 2025) into elite maize cultivars by breeding is one example; another is introgression of drought resilience into dryland rice to improve yield through breeding. However, timescales for the success of breeding programs, especially for complex traits where there is likely to be a yield tradeoff and for species with long reproduction cycles such as forest trees, are unpredictable. Moreover, cross-species introduction of traits or the use of new-to-nature processes obviously require an engineering approach. This means that parallelization of different potential solutions is needed.

In some cases, alternative interventions could have much greater potential for overall emissions reductions. For example, a large-scale switch to more plant-rich diets and decreasing food waste in developed countries could dramatically reduce GHG emissions (Foley et al. 2011; Poore and Nemecek 2018; FAO 2022a, 2022b; IPCC 2022a, 2022b) and would deliver fivefold higher annual CH_4_ emissions reductions from decreased ruminant animal use than from reducing methane emissions from rice cultivation (Smith et al. 2021). However, such cultural shifts are challenging and presently not realistic at the scales and timeframes required. Indeed, we are seeing an increase in consumption of animal protein globally per capita (FAO 2023; OECD/FAO 2023; Ritchie et al. 2023).

Non-synthetic biology alternatives to decrease CH_4_ emissions from the burning of crop residues include incorporating residue into soils to increase soil carbon (although this will eventually be liberated into the atmosphere), incorporation into useful materials (durability depends on the material), and converting residues into biochar. Biochar is made by slow baking in a kiln that keeps the biomass separate from the flame and oxygen, preventing direct combustion. The resulting highly carbonized organic matter is virtually nondegradable. Over the longer term, biochar forms sedimentary rocks, which are stable over multi-million-year timescales, thus removing carbon from biosphere carbon cycles and sequestering it into the geosphere (Sanei et al. 2024). Biochar can also be used to improve soil water holding capacity (due to its porosity) and soil fertility. A key advantage of the biochar process is the production of higher-value side stream products, including bio-oil (which can be used as a fuel or a chemical synthesis feedstock, or for carbon sequestration) and wood vinegar (pyroligneous acid, used as a bio fertilizer and bio pesticide). Some biogas is also made and can be captured if there is a sufficient amount to make it worthwhile. Overall, this makes for a positive technoeconomic analysis. Estimates place the potential of carbon sequestration by biochar between 0.05 and 2 Gt CO_2_ yr^−1^ (Fuss et al. 2018; Barlow et al. 2024). Our analysis calculated that 162 mha cropland would be required to deliver sufficient sustainably harvested biomass to sequester 1 Gt CO_2_e yr^−1^ (Table 3). This is 15% of the global land used for annual crops that have crop residues (FAO 2021). As noted in Table 3, infrastructure and logistics remain a significant challenge for rolling out biochar generation on this scale. In particular, it is necessary to develop distributed infrastructure to avoid transport costs, which would negatively affect economic analysis (Sahoo et al. 2021; Gamaralalage et al. 2025). Moreover, the environmental release of biochar may also cause environmental pollution and adverse effects on organisms (Dong et al. 2025). This necessitates careful analysis of the environmental fate of biochar to ensure its safe and sustainable development. Another consideration for biochar is energy demands for different processes. Depending on different conditions (feedstock type, feedstock condition, kiln design, temperature, time period, policy drivers, source and cost of energy), energy demands, emissions, and profitability can be quite variable (Harsono et al. 2013; Peters et al. 2015; Schaffer et al. 2019; Cornelissen et al. 2023; Patel and Panwar 2024). Slow pyrolysis at high temperatures (optimal for biochar production) requires higher energy inputs but may become self-sustaining (Schaffer et al. 2019). Pyrolysis can be far more emissions-friendly than open burning: for example, life-cycle assessment of a slow pyrolysis system found that the biochar system had negative GHG emissions, including a reduction in cumulative nonrenewable energy demand (Peters et al. 2015).

Fossil fuels account for over 75% of total global GHG emissions and nearly 90% of all CO_2_ emissions (WRI 2025). Replacing fossil resources is nontrivial given that the bulk of our global industrial system relies on them to some extent (Michaux 2024). Although synthetic biology has been successfully applied to produce bioethanol and sustainable aviation fuels and to improve lignocellulosic biofuel production, reaching the required scale and cost-effectiveness has remained challenging. While plant-based biofuels are an important component of displacing the use of fossil fuels, combustion of the resulting molecules releases carbon back into the atmosphere and contributes to emissions. A transition to renewable energy (wind, solar) could potentially deliver 90% of the emissions reductions required and has been proposed as the most cost-effective and scalable solution for emissions reduction (IRENA 2019). However, some of the assumptions underlying the requirements for raw materials, technology and infrastructure development, workforce, land use changes, and societal impacts (among others) for such a transition have been challenged, shedding doubt that the current timescales envisioned for transition are accurate (Michaux 2024).

In the last decade or two, there has been great excitement about, and funding directed toward, living off-planet (i.e. beyond Earth). This includes a significant focus on engineering crop plants for extraplanetary living. These efforts are likely to deliver innovations in plant synthetic biology and intensification of production systems, which could contribute to emissions reductions and/or carbon sequestration objectives. However, extraplanetary colonization is well beyond the event horizon required for climate change objectives on Earth; focusing on this as an end goal therefore distracts from much more urgent objectives. Making a non-Earth location habitable would be far more costly, take much longer, and be far less feasible than solving the problems we have here on Earth today.

## Conclusions and forwards directions

We discussed current and potential future plant synthetic biology approaches that could meaningfully (in the context of climate change) help mitigate GHG emissions and increase carbon drawdown. Our broader analysis framework for comparison of different interventions (Fermi estimation plus considerations outlined in Box 1) is useful for identifying and prioritizing climate-relevant interventions, but many questions remain (Outstanding Questions box). We explore these questions, along with other considerations, in the following.

Fermi analyses confirmed that synthetic biology has the potential to provide unique, high-impact leverage points for carbon sequestration in agriculture. However, many of the more innovative approaches require major advances in metabolic and genetic engineering. Accelerating the design and generation of engineered plants will be critical, especially in nonmodel species and plants with longer generation times such as trees. Trait analysis in field trials is also critical and takes time to explore. Moreover, some engineered traits will have a physiological tradeoff, potentially negating advantages. Some nonbiological solutions would have a much greater and much faster impact on climate change (e.g. dietary changes, waste reduction) but appear unlikely to be implemented quickly enough or at sufficient scale. Altering planetary albedo (the solar radiation that is reflected back into space) through atmospheric and/or surface geoengineering have been proposed to improve the planet's radiation balance and mitigate climate change—albeit not without challenges, uncertainties, and controversy (Wigley 2006; Shepherd 2009; Foley et al. 2011; Irvine et al. 2011, 2016; MacMartin et al. 2018; Grasso 2022; Sen 2025).

Comparative Fermi analysis (Tables 2, 3, 4) clearly showed that deployment area is a driving force for the scalability and impact potential of biological solutions. Land-use tradeoffs are also central considerations for biological solutions. Nonsynthetic biology biomass solutions such as reforestation, afforestation and fast-growth biomass species (e.g. *Miscanthus*) offer enormous potential but require land use changes for scalability. *Miscanthus* and other stress-tolerant crops have the advantages of extraordinarily fast growth, high yield, and marginal land use but short carbon durability under current end use plans.

Crop-based solutions for engineered biomass and yield improvements are notable in that they leverage existing agricultural/forestry systems and land use scenarios, offering reduced disruption, increased feasibility, and financial co-benefits to the sector. However, they depend on trait maintenance over long periods and our ability to simultaneously address carbon and/or nutrient limitations to mitigate detrimental growth impacts in the field. While Fermi analysis demonstrated high potential for engineered high-yield forestry trees, genetic engineering in these species is a cost- and time-intensive process (Whitehill and Bohlmann 2019; Gagalova et al. 2022), making this strategy a long-term goal. Engineering in broadacre crops with large deployment area potential can happen relatively quickly in species with established engineering approaches, including rice, maize, wheat, barley (*Hordeum vulgare*), soybean (*Glycine max*), cotton (*Gossypium hirsutum*), and sorghum (*Sorghum bicolor*). This would also help meet food, feed, and fiber priorities. Resource competition (especially for land and water) will intensify as the global population continues to increase, with peak population expected in the mid-2080s (United Nations 2024). This necessitates a careful balancing between sequestration-only strategies (reforestation, afforestation) and strategies with co-benefits (crop production). Carbon sequestration solutions that simultaneously improve food yield, such as decreasing harvest losses, become even more attractive in this scenario.

No single solution provides all the required mitigation, and stacking of strategies across systems and timeframes is necessary to approach global targets. Technology readiness also varies widely: mature strategies (biochar, nonengineered forestry and biomass; Table 3) are deployable now and can deliver near-term impact, while others—especially early-stage engineering strategies (Tables 2, 4)—have low technological readiness but have transformational potential in the longer term if established. Moreover, different strategies will suit different locations, geographies, climates, and so on.

**Figure 5.**
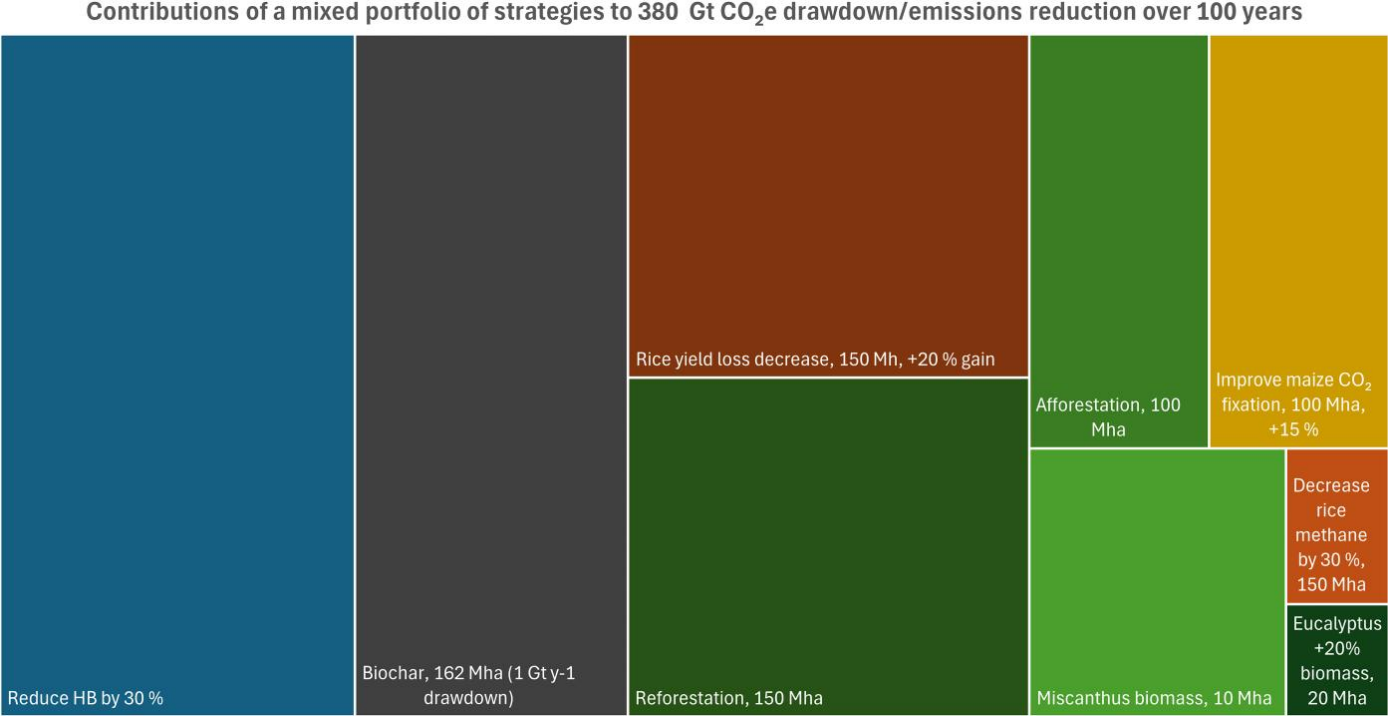
A mixed portfolio of bio-based carbon drawdown strategies could deliver 420 gt CO_2_e drawdown over 100 years. Combining the nature-based and synthetic biology carbon drawdown strategies explored in Tables 2, 3, and 4 has the potential to deliver ∼380 Gt CO_2_e drawdown over a 100-year horizon. Reducing our reliance on the HB process for nitrogen fertilizers and implementation of biochar have the largest individual contributions, with the potential to deliver Gt per annum CO2e impact. A reduction of yield losses by 20% in the global rice paddy would deliver similar sequestration potential to reforestation over 150 mha. Improved CO_2_ fixation across the USA, China, and Brazil maize crops would deliver a similar sequestration potential to afforestation. Miscanthus is notable for its large impact over an area that is 1/10th the area required for other large sequestration opportunities. Strategies with smaller sequestration/reduction potential detailed in Tables 2, 3, and 4 are treated as follows for this figure: (i) For biochar, Australian wheat stover and the conversion of burning agricultural residue to biochar are incorporated into the modeled 1 Gt biochar drawdown figure. The latter would deliver∼1/3 of the required biomass to achieve 1 Gt/annum drawdown. (ii) Improved CO_2_ fixation in USA maize is incorporated into the increased CO_2_ maize fixation across the three largest maize-producing countries (USA, China, and Brazil). (iii) Decreased yield loss at pilot scale is incorporated into the global rice crop number (iv) Global forestry increases of +20% are not included due to the timescale required to develop engineering strategies and because successful yield increases across different tree species are outside the 100-year horizon; poplar plantations are excluded due to relatively small drawdown; and Eucalyptus plantations are included due to relative feasibility and large drawdown. (v) Sporopollenin is omitted for both *Miscanthus* due to its uncertain value proposition, and for maize because it is likely that soil carbon storage saturation would occur relatively early in the 100-year event horizon, so it would not deliver the modeled 100-year contribution.

It is important to consider where effort is best invested to deliver impactful outcomes. Nonetheless, even where individual technologies have relatively moderate potential, they can contribute to ensemble solutions. These observations align with the well-recognised understanding that a mixed portfolio of technologies is necessary to achieve carbon drawdown goals (FAO 2022a, 2022b, 2023; IPCC 2022a, 2022b). When all the strategies we examined that could provide a meaningful contribution are combined, assuming deployment over a 100-year timeframe at the largest scale explored, they represent a 380 Gt CO_2_e impact potential (Fig. 5). Engineering to decrease yield losses in the global rice paddy, improve CO_2_ fixation in the USA/Brazil/China maize crop, and engineering a 20% increase in eucalyptus biomass are all targets with high potential and could deliver 120 Gt of this impact. Synthetic biology solutions to decrease CH_4_ emissions from rice and our dependency on the HB process could contribute to another ∼140 Gt CO_2_e impact. Reducing the HB process and the implementation of biochar have the largest individual contributions, with the potential to deliver Gt per annum CO_2_e impact.

Engineered alternatives to the HB process represent one of the most ambitious, high-reward strategies in the plant synthetic biology toolkit for climate mitigation. To maximize reductions in synthetic nitrogen use, the implementation of both short-term solutions, such as increasing the deployment of leguminous cover crops (Oliveira-Filho et al. 2025) and engineered N-fixing microbiomes, and long-term solutions, such as engineering nonlegume plants to fix N, will be needed.

Many of the strategies examined in the previous sections are compatible and can be layered across space and/or time to enhance net sequestration and storage durability while diversifying risk. For example, photosynthetic improvements in maize could be paired with sporopollenin expression, decreases in CH_4_ emissions could be paired with decreases in yield losses, and *Miscanthus* could be coupled with biochar pathways.

As noted, storage durability is also important for long-term impact in carbon sequestration strategies. The generation of biochar is a good immediate solution to increase the long-term storage of photosynthetically fixed carbon and offers numerous co-benefits, but will also require careful assessment of potential ecological impacts (Dong et al. 2025). Resistant biopolymers offer an exciting opportunity to increase durability and soil carbon in the right contexts, as long as yields are not significantly impacted and sufficient deployment biomass/area can be achieved. Cross-sectoral integration—for instance, incorporating crop biomass into bio-based materials and linking to construction industries—could decrease emissions from other sectors, contribute to biological drawdown, and provide pathways to durable carbon storage with a longer-term impact.

**Figure 6.**
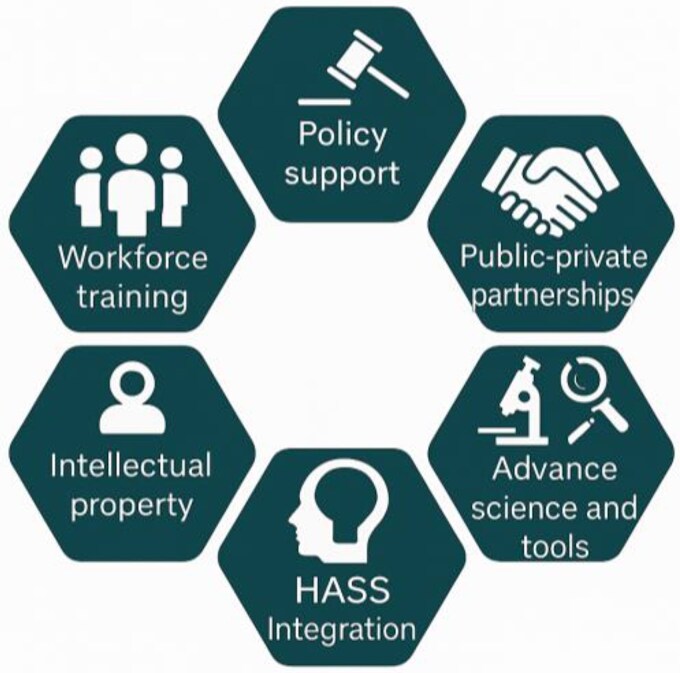
Broader considerations for enabling impact from plant synthetic biology interventions. In addition to technical and economic factors, successful implementation of synbio-based climate solutions in agriculture requires attention to broader enablers of real-world impact. Key considerations include: (i) *Workforce training*—building problem-oriented, engineering-informed capacity in plant science; (ii) *Policy support*—ensuring regulatory and economic frameworks incentivize sustainable technology development; (iii) *Public–private partnerships*—leveraging cross-sector collaboration to accelerate translation and scale; (iv) *HASS integration*—embedding understanding of social, ethical, cultural, and behavioral dynamics in technology design and deployment; and (v) *Intellectual property*—creating fair, realistic models for IP ownership and commercialization. Figure created using ChatGPT.

Technological development does not occur in a vacuum, and an integrated interdisciplinary approach is required to bring about real word solutions (Gray et al. 2018; Wurtzel et al. 2019; Rhee et al. 2025). In conjunction with pursuing high-potential technical targets, there are numerous other philosophical, sociological, economic, market, regulatory, workforce, and policy-based factors to be considered. Some of these considerations are embedded in the post-Fermi evaluative criteria explored in Box 1. Importantly, for the climate change objectives considered here, LCA is critical to identify unanticipated offsets and emissions sources. Box 3 explores some of the broader considerations for achieving rapid and impactful implementation of synthetic biology strategies for plant agriculture-based climate change solutions (summarized in Fig. 6).

Box 3. Addressing some of the broader considerations Biotechnology solutions can only be effective in the long term if there is a realistic pathway to impact, including a social license to operate. Some key considerations are:

**Workforce training.** In modern plant synthetic biology remains a limitation. As Patron (2020) notes, reaching the full potential will require conscious adjustments to the skillsets and mind sets of plant scientists. Particularly important is training in problem-orientated approaches, which are common in engineering disciplines but less so in the sciences. An understanding of how to engage with and communicate to the broader public around synthetic biology technologies and their implementation is also critical (Carter et al. 2021; Dixson et al. 2025).

**Policy support.** The cost of many solutions, including development, testing, and regulatory approval, is significant. Compared to the fossil resource industry, large-scale subsidies are limited for competing replacement technologies. Outside of carbon taxes applied in several countries, the establishment of sustainable technologies must overcome this additional economic incentive handicap. Agile regulatory policy is also required to respond to emerging technologies. The knock-on effects of policy interventions must also be assessed.

**Public–private partnerships.** Expanding efforts to collaborate across academia, industry, and government research has great potential to speed up innovations and their translation into real word applications. Speed, agility, and more predictable funding can be achieved through the private sector and joint academia-industry projects.

**Intellectual property.** Complexities around intellectual property (IP) ownership and exploitation need to be addressed. The risks associated with monetarizing IP are often under-recognized by academic organizations, resulting in over-valuation and unreasonable benefit sharing demands. Good models for technology transfer should be identified and implemented.

**Advancing fundamental science and tools.** Much fundamental knowledge of how plants regulate tradeoffs between growth and ecological adaptation and mediate dynamic interactions with other organisms and the abiotic environment has yet to be generated within and especially outside established model systems. A better understanding of the effects of combined stressors, trait interconnectedness, and of pleiotropic outcomes is also required. Better predictive AI and LLM tools are also needed to improve the accuracy of gene function annotations and better exploit the vast genome repositories now available.

**HASS integration.** To build pathways to impact, it is necessary to understand the broader social, cultural, environmental, ethical, legal, institutional, and political considerations around the desired technologies and the desired impacts. In particular, understanding how peoples’ beliefs and values influence their responses to synthetic biology technologies is extremely important for understanding the feasibility of implementation (Mankad et al. 2021).

Our climate is rapidly changing, as are many of the factors contributing to the evaluative criteria outlined in Fig. 1/Box 1 and the broader considerations outlined in Fig. 6/Box 3. New information and considerations that may affect the cost–benefit analysis of any given solution will also emerge over time. Consequently, solutions that appear attractive in 2025 may be less so in a decade (or even half a decade), and vice-versa. This necessitates regular re-evaluation and re-prioritization of strategies. We are the custodians of this very beautiful and unique (at least, to our knowledge) planet that we live on. The impact of human-driven climate change can now be felt worldwide (IPCC 2023). As a self-aware species, it is our responsibility to change behaviors harmful to planetary and human health and seek to ensure a safe and vibrant environment for future generations. While this task seems mighty indeed given the magnitude of the problem, the scientific tools to effect change are now at our disposal. A realistic climate plan requires both mature, immediately deployable solutions and longer-term innovation pipelines. The question of whether there is enough time may seem paralyzing, but lessons of the past suggest we have reason to be positive: “*We always overestimate the change that will occur in the next two years and underestimate the change that will occur in the next ten. Don't let yourself be lulled into inaction.*”—Bill Gates

Advances Box

- Plant synthetic biology has matured, with access to robust, predictable, and tunable genetic components and circuitry that allow rapid, precise, spatiotemporally resolved engineering of complex traits in crop plants and key microbiome species
- Advanced integrated ‘omics technologies allow us to sensitively and specifically examine metabolism, identify targets, and quantify intervention effects.
- Greater understanding of the complex interactions between plants and their environment (both biotic and abiotic) provides critical insights for developing solutions.
- Implementation of quantitative approaches, such as Fermi analysis, allow early-stage benchmarking and prioritization of climate-relevant synthetic biology interventions.
- Rapidly advancing AI technologies will accelerate data integration and improve the identification and prioritization of meaningful targets Do we have enough time?—and, how can we accurately predict the time requirements for generation and implementation of different plant synthetic biology solutions?

Outstanding Questions Box

- How can Fermi analysis, TEA and LCA be integrated into R&D pipelines to prioritize impactful and feasible interventions?
- How can we further accelerate the design, generation, and testing of engineered plants?
- How can synthetic biology traits be engineered for durability and stable performance in different field conditions and over many generations?
- What strategies (biological and nonbiological, engineered and nonengineered) can mitigate yield penalties and other tradeoffs when crops are engineered for increased carbon sequestration, reduced emissions, and/or carbon storage?
- What governance, regulatory, and socio-economic frameworks are needed to accelerate adoption of climate-relevant plant engineering?

## Supplementary Material

kiaf410_Supplementary_Data

## Data Availability

All data are incorporated into the article and its online supplementary material.

## Footnotes

1 Gartner's hype cycle explores the maturity, adoption and social application of specific technologies following an innovation trigger. It characterizes the over-enthusiasm (“hype”) that commonly happens, along with subsequent phases of technology response. It can be graphicly represented with a sharp “peak of inflated expectations” followed by a “trough of disillusionment”, then a gentle “slope of enlightenment” and finally a “plateau of productivity” (Linden and Fenn 2003).

## Supplementary data

The following materials are available in the online version of this article.

**Supplementary File.** Fermi Analysis. Includes Approach as well as Fermi Estimations, Interpretation, and Notes for all scenarios explored. Supplementary tables are embedded in this document.

**Supplementary Table S1.** Estimated carbon fixation in global croplands based on Gross Primary Production (GPP) and Net Primary Production (NPP).

**Supplementary Table S2.** Assumptions used to estimate the carbon sequestration potential of engineered sporopollenin deposition in maize roots.

**Supplementary Table S3.** Assumptions used to estimate the carbon sequestration potential of engineered sporopollenin deposition in Miscanthus in Europe.

**Supplementary Table S4.** Sporopollenin mass, carbon stored, CO_2_-equivalent, and total CO_2_ sequestered in different tissues of US maize and European Miscanthus.

**Supplementary Table S5.** Assumptions used to estimate the additional carbon drawdown potential from expanded Miscanthus cultivation.

**Supplementary Table S6.** Assumptions used to estimate the carbon sequestration potential improved CO_2_ fixation in US maize.

**Supplementary Table S7.** Estimated carbon sequestration potential from improved photosynthetic CO_2_ fixation in U.S. field corn (maize).

**Supplementary Table S8.** Assumptions used to estimate the carbon mitigation potential from increasing per-hectare yield of rice.

**Supplementary Table S9.** Assumptions used to estimate carbon drawdown potential from genetically engineered poplar with reduced photorespiration.

**Supplementary Table S10.** Assumptions and calculations for 100-year additional CO_2_ sequestration from engineered Eucalyptus plantations.

**Supplementary Table S11.** Assumptions and calculations for 100-year additional CO_2_ sequestration if the entire global forestry plantation was engineered.

**Supplementary Table S12.** Biochar-Based CO_2_e Drawdown from Australian Wheat Stover.

**Supplementary Table S13.** Assumptions used to estimate the carbon drawdown potential of biochar-based sequestration.

**Supplementary Table S14.** Assumptions used to estimate the carbon drawdown potential of reforestation.

**Supplementary Table S15.** Assumptions used to estimate the carbon drawdown potential of afforestation.

**Supplementary Table S16.** Estimated CO_2_e mitigation potential from reducing methane emissions in global paddy rice cultivation.

**Supplementary Table S17.** Estimated climate mitigation potential of converting agricultural residue burning to biochar production.

**Supplementary Table S18.** Estimated mitigation potential from replacing the Haber–Bosch process and fertilizer-associated emissions with synthetic biology-derived nitrogen fixation or other low-emission alternatives.

## References

[kiaf410-B1] Alexandrov K, Vickers CE. *In vivo* protein-based biosensors: seeing metabolism in real time. Trends Biotechnol. 2023:41(1):19–26. 10.1016/j.tibtech.2022.07.002 35918219

[kiaf410-B2] Amthor JS, Bar-Even A, Hanson AD, Millar AH, Stitt M, Sweetlove LJ, Tyerman SD. Engineering strategies to boost crop productivity by cutting respiratory carbon loss. Plant Cell. 2019:31(2):297–314. 10.1105/tpc.18.00743 30670486 PMC6447004

[kiaf410-B3] Andres J, Blomeier T, Zurbriggen MD. Synthetic switches and regulatory circuits in plants. Plant Physiol. 2019:179(3):862–884. 10.1104/pp.18.01362 30692218 PMC6393786

[kiaf410-B4] Atalay FE, Culum AA, Kaya H, Gokturk G, Yigit E. Different plant sporopollenin exine capsules and their multifunctional usage. ACS Appl Bio Mater. 2022:5(3):1348–1360. 10.1021/acsabm.2c00071 PMC8941510 35201750

[kiaf410-B5] Atkinson AW, Gunning BES, John PCL. Sporopollenin in the cell wall of Chlorella and other algae: ultrastructure, chemistry, and incorporation of 14C-acetate, studied in synchronous cultures. Planta. 1972:107(1):1–32. 10.1007/BF00398011 24477346

[kiaf410-B6] Bar-Even A . Daring metabolic designs for enhanced plant carbon fixation. Plant Sci. 2018:273:71–83. 10.1016/j.plantsci.2017.12.007 29907311

[kiaf410-B7] Bar-Even A, Noor E, Savir Y, Liebermeister W, Davidi D, Tawfik DS, Milo R. The moderately efficient enzyme: evolutionary and physicochemical trends shaping enzyme parameters. Biochemistry. 2011:50(21):4402–4410. 10.1021/bi2002289 21506553

[kiaf410-B8] Barlow J, Kane D, Hottle R, Mehra M, Toensmeier E, Frischmann C. Biochar Production. Project Drawdown; 2024 [accessed 2024/01/08]. https://drawdown.org/solutions/biochar-production

[kiaf410-B9] Bastin J-F, Finegold Y, Garcia C, Mollicone D, Rezende M, Routh D, Zohner CM, Crowther TW. The global tree restoration potential. Science. 2019:365(6448):76–79. 10.1126/science.aax0848 31273120

[kiaf410-B10] Bathe U, Leong BJ, Van Gelder K, Barbier GG, Henry CS, Amthor JS, Hanson AD. Respiratory energy demands and scope for demand expansion and destruction. Plant Physiol. 2023:191(4):2093–2103. 10.1093/plphys/kiac493 36271857 PMC10069906

[kiaf410-B11] Beaulieu C, Sidibé A, Jabloune R, Simao-Beaunoir AM, Lerat S, Monga E, Bernards MA. Physical, chemical and proteomic evidence of potato suberin degradation by the plant pathogenic bacterium Streptomyces scabiei. Microbes Environ. 2016:31(4):427–434. 10.1264/jsme2.ME16110 27853060 PMC5158115

[kiaf410-B12] Belt K, Obe D, Wilson MA, Millar AH, Bathe U. Harnessing mass spectrometry-based proteomics for continuous directed evolution. bioRxiv 2025.03.03.641153. 10.1101/2025.03.03.641153 05 march 2025, preprint: not peer reviewed. PMC12765272 41492657

[kiaf410-B13] Bick MJ, Greisen PJ, Morey KJ, Antunes MS, La D, Sankaran B, Reymond L, Johnsson K, Medford JI, Baker D. Computational design of environmental sensors for the potent opioid fentanyl. eLife. 2017:6:e28909. 10.7554/eLife.28909 28925919 PMC5655540

[kiaf410-B14] Boo A, Toth T, Yu Q, Pfotenhauer A, Fields BD, Lenaghan SC, Stewart CN, Voigt CA. Synthetic microbe-to-plant communication channels. Nat Commun. 2024:15(1):1817. 10.1038/s41467-024-45897-6 38418817 PMC10901793

[kiaf410-B15] Borowsky AT, Bailey-Serres J. Rewiring gene circuitry for plant improvement. Nat Genet. 2024:56(8):1574–1582. 10.1038/s41588-024-01806-7 39075207

[kiaf410-B16] Bouwmeester H, Zerbe P, Peters RJ, Wang K, Dong L. The role of isoprenoids in the chemical interaction between plants and other organisms in their rhizosphere. aBIOTECH. 2025. 10.1007/s42994-025-00225-4 PMC12647503 41312096

[kiaf410-B17] Brophy JAN, Magallon KJ, Duan L, Zhong V, Ramachandran P, Kniazev K, Dinneny JR. Synthetic genetic circuits as a means of reprogramming plant roots. Science. 2022:377(6607):747–751. 10.1126/science.abo4326 35951698

[kiaf410-B18] Brunner C, Hausfather Z, Knutti R. Durability of carbon dioxide removal is critical for Paris climate goals. Commun Earth Environ. 2024:5(1):645. 10.1038/s43247-024-01808-7

[kiaf410-B19] Burén S, Young EM, Sweeny EA, Lopez-Torrejón G, Veldhuizen M, Voigt CA, Rubio LM. Formation of nitrogenase NifDK tetramers in the mitochondria of Saccharomyces cerevisiae. ACS Synth Biol. 2017:6(6):1043–1055. 10.1021/acssynbio.6b00371 28221768 PMC5477005

[kiaf410-B20] Bwire D, Saito H, Sidle RC, Nishiwaki J. Water management and hydrological characteristics of paddy-rice fields under alternate wetting and drying irrigation practice as climate smart practice: a review. Agronomy. 2024:14(7):1421. 10.3390/agronomy14071421

[kiaf410-B21] Carrijo DR, Lundy ME, Linquist BA. Rice yields and water use under alternate wetting and drying irrigation: a meta-analysis. Field Crops Res. 2017:203:173–180. 10.1016/j.fcr.2016.12.002

[kiaf410-B22] Carter L, Mankad A, Hobman EV, Porter NB. Playing God and tampering with nature: popular labels for real concerns in synthetic biology. Transgenic Res. 2021:30(2):155–167. 10.1007/s11248-021-00233-2 33502671

[kiaf410-B23] Center for Climate and Energy Solutions . Global emissions. Center for Climate and Energy Solutions; 2024 [accessed 2025 Mar 17]. https://www.c2es.org/content/international-emissions/

[kiaf410-B24] Chakraborty S, Venkataraman M, Infante V, Pfleger BF, Ané J-M. Scripting a new dialogue between diazotrophs and crops. Trends Microbiol. 2024:32(6):577–589. 10.1016/j.tim.2023.08.007 37770375 PMC10950843

[kiaf410-B25] Chen L, Msigwa G, Yang M, Osman AI, Fawzy S, Rooney DW, Yap P-S. Strategies to achieve a carbon neutral society: a review. Environ Chem Lett. 2022:20(4):2277–2310. 10.1007/s10311-022-01435-8 35431715 PMC8992416

[kiaf410-B26] Cifuentes García R, Galán G, Martín M. Multiscale analysis for the valorization of biomass via pellets production towards energy security. J Clean Prod. 2024:461:142663. 10.1016/j.jclepro.2024.142663

[kiaf410-B27] ClimateWatch . Historical GHG Emissions. ClimateWatch; 2025 [accessed 2025 Mar 17]. https://www.climatewatchdata.org/

[kiaf410-B28] Cornelissen G, Sørmo E, de la Rosa RKA, Ladd B. Flame curtain kilns produce biochar from dry biomass with minimal methane emissions. Sci Total Environ. 2023:903:166547. 10.1016/j.scitotenv.2023.166547 37640066

[kiaf410-B29] Crawford DF, O’Connor MH, Jovanovic T, Herr A, Raison RJ, O’Connell DA, Baynes T. A spatial assessment of potential biomass for bioenergy in Australia in 2010, and possible expansion by 2030 and 2050. Glob Change Biol Bioenergy. 2016:8(4):707–722. 10.1111/gcbb.12295

[kiaf410-B30] Crippa M, Solazzo E, Guizzardi D, Monforti-Ferrario F, Tubiello FN, Leip A. Food systems are responsible for a third of global anthropogenic GHG emissions. Nat Food. 2021:2(3):198–209. 10.1038/s43016-021-00225-9 37117443

[kiaf410-B31] de Lange O, Klavins E, Nemhauser J. Synthetic genetic circuits in crop plants. Curr Opin Biotechnol. 2018:49:16–22. 10.1016/j.copbio.2017.07.003 28772191 PMC6007868

[kiaf410-B32] Dixson HGW, Waldby C, Raman S, Mackenzie A, Carter L. Tragic Flaws and Practical Wisdom: public reasoning behind preferences for different genetic technologies. Public Underst Sci. 2025:09636625251333316. 10.1177/09636625251333316 PMC12535613 40340608

[kiaf410-B33] Dohleman FG, Heaton EA, Arundale RA, Long SP. Seasonal dynamics of above- and below-ground biomass and nitrogen partitioning in Miscanthus × giganteus and Panicum virgatum across three growing seasons. Glob Change Biol Bioenergy. 2012:4(5):534–544. 10.1111/j.1757-1707.2011.01153.x

[kiaf410-B34] Dong M, Jiang M, He L, Zhang Z, Gustave W, Vithanage M, Niazi NK, Chen B, Zhang X, Wang H, et al Challenges in safe environmental applications of biochar: identifying risks and unintended consequence. Biochar. 2025:7(1):12. 10.1007/s42773-024-00412-4

[kiaf410-B35] Dronsella B, Orsi E, Schulz-Mirbach H, Benito-Vaquerizo S, Yilmaz S, Glatter T, Bar-Even A, Erb TJ, Claassens NJ. One-carbon fixation via the synthetic reductive glycine pathway exceeds yield of the Calvin cycle. Nat Microbiol. 2025:10(3):646–653. 10.1038/s41564-025-01941-9 40016510 PMC11879842

[kiaf410-B36] Du Y, Lu Y, Guo S, Wang R, Song X, Ju X. Enhanced efficiency nitrogen fertilizers (EENFs) can reduce nitrous oxide emissions and maintain high grain yields in a rain-fed spring maize cropping system. Field Crops Res. 2024:312:109408. 10.1016/j.fcr.2024.109408

[kiaf410-B37] Eckardt NA, Ainsworth EA, Bahuguna RN, Broadley MR, Busch W, Carpita NC, Castrillo G, Chory J, DeHaan LR, Duarte CM, et al Climate change challenges, plant science solutions. Plant Cell. 2023:35(1):24–66. 10.1093/plcell/koac303 36222573 PMC9806663

[kiaf410-B38] Faggiani Dias D, Hanna R, Sachnik J, Xu Y, Gilbert J, Busch W, Victor DG. Removing atmospheric CO2 through mass scaleup of crops with enhanced root systems. Environ Res Lett. 2025:20(5):054004. 10.1088/1748-9326/adc31b

[kiaf410-B39] FAO . Global forest resources assessment 2020. Rome (Italy): Food and Agriculture Organization of the United Nations; 2020. https://openknowledge.fao.org/server/api/core/bitstreams/9f24d451-2e56-4ae2-8a4a-1bc511f5e60e/content

[kiaf410-B40] FAO . FAOSTAT: land use statistics. Rome (Italy): Food and Agriculture Organization of the United Nations; 2021. https://www.fao.org/statistics/highlights-archive/highlights-detail/land-statistics-2001-2022.-global--regional-and-country-trends/en

[kiaf410-B41] FAO . FAO strategy on climate change 2022–2031. Rome (Italy): Food and Agriculture Organization of the United Nations; 2022a. https://www.fao.org/climate-change/en/

[kiaf410-B42] FAO . FAOSTAT: climate change—agrifood systems emissions. Rome (Italy): Food and Agriculture Organization of the United Nations; 2022b. https://openknowledge.fao.org/items/00ede01a-d144-404c-9866-76bb8f25220c

[kiaf410-B43] FAO . FAO food balance sheets (2010–2022). Rome (Italy): Food and Agriculture Organization of the United Nations (FAO); 2023. https://www.fao.org/statistics/highlights-archive/highlights-detail/food-balance-sheets-2010-2022-global-regional-and-country-trends/en

[kiaf410-B44] Fernie AR, Bachem CWB, Helariutta Y, Neuhaus HE, Prat S, Ruan Y-L, Stitt M, Sweetlove LJ, Tegeder M, Wahl V, et al Synchronization of developmental, molecular and metabolic aspects of source–sink interactions. Nat Plants. 2020:6(2):55–66. 10.1038/s41477-020-0590-x 32042154

[kiaf410-B45] Foley JA, Ramankutty N, Brauman KA, Cassidy ES, Gerber JS, Johnston M, Mueller ND, O’Connell C, Ray DK, West PC. Solutions for a cultivated planet. Nature. 2011:478(7369):337–342. 10.1038/nature10452 21993620

[kiaf410-B46] Frederick E. Designing plants that don't decay. Boston: MIT; 2020 [accessed 2025 Mar 19]. https://wi.mit.edu/news/designing-plants-dont-decay

[kiaf410-B47] Friedlingstein P, O'Sullivan M, Jones MW, Andrew RM, Hauck J, Landschützer P, Le Quéré C, Li H, Luijkx IT, et al Global carbon budget 2024. Earth Syst Sci Data. 2025:17(3):965–1039. 10.5194/essd-17-965-2025

[kiaf410-B48] Fuss S, Lamb WF, Callaghan MW, Hilaire J, Creutzig F, Amann T, Beringer T, de Oliveira Garcia W, Hartmann J, Khanna T, et al Negative emissions—part 2: costs, potentials and side effects. Environ Res Lett. 2018:13(6):063002. 10.1088/1748-9326/aabf9f

[kiaf410-B49] Gagalova KK, Warren RL, Coombe L, Wong J, Nip KM, Yuen MMS, Whitehill JGA, Celedon JM, Ritland C, Taylor GA, et al Spruce giga-genomes: structurally similar yet distinctive with differentially expanding gene families and rapidly evolving genes. Plant J. 2022:111(5):1469–1485. 10.1111/tpj.15889 35789009

[kiaf410-B50] Galindo-Castañeda T, Brown KM, Lynch JP. Reduced root cortical burden improves growth and grain yield under low phosphorus availability in maize. Plant Cell Environ. 2018:41(7):1579–1592. 10.1111/pce.13197 29574982

[kiaf410-B51] Gamaralalage D, Rodgers S, Gill A, Meredith W, Bott T, West H, Alce J, Snape C, McKechnie J. Biowaste to biochar: a techno-economic and life cycle assessment of biochar production from food-waste digestate and its agricultural field application. Biochar. 2025:7(1):50. 10.1007/s42773-025-00456-0 40078517 PMC11893672

[kiaf410-B52] Garcia A, Gaju O, Bowerman AF, Buck SA, Evans JR, Furbank RT, Gilliham M, Millar AH, Pogson BJ, Reynolds MP, et al Enhancing crop yields through improvements in the efficiency of photosynthesis and respiration. New Phytol. 2023:237(1):60–77. 10.1111/nph.18545 36251512 PMC10100352

[kiaf410-B53] Ge M, Friedrich J, Vigna L. Where do emissions come from? 4 charts explain greenhouse gas emissions by sector. Washington (DC): World Resources Institute; 2024. https://www.wri.org/insights/4-charts-explain-greenhouse-gas-emissions-countries-and-sectors#:∼:text=Agriculture is the second highest,3.4%25 of the global total

[kiaf410-B54] Glinkerman CM, Lin S, Ni J, Li F-S, Zhao X, Weng J-K. Sporopollenin-inspired design and synthesis of robust polymeric materials. Commun Chem. 2022:5(1):110. 10.1038/s42004-022-00729-w 36697794 PMC9814627

[kiaf410-B55] Good BH, Chapman RL. The ultrastructure of Phycopeltis (Chroolepidaceae: Chlorophyta). I. Sporopollenin in the cell walls. Am J Bot. 1978:65(1):27–33. 10.1002/j.1537-2197.1978.tb10830.x

[kiaf410-B56] Gowda P, Steiner JL, Olson C, Boggess M, Farrigan T, Grusak MA. Agriculture and rural communities. In: Reidmiller DR, Avery CW, Easterling DR, Kunkel KE, Lewis KLM, Maycock TK, Stewar BC, editors. Impacts, risks, and adaptation in the United States: Fourth National Climate Assessment, Vol. II. Washington (DC, USA): U.S. Global Change Research Program; 2018. p. 391–437.

[kiaf410-B57] GRAIN . Land grabbing for biofuels must stop. Barcelona (Spain): GRAIN; 2013. https://grain.org/en/article/4653-land-grabbing-for-biofuels-must-stop

[kiaf410-B58] Grasso M . Legitimacy and procedural justice: how might stratospheric aerosol injection function in the public interest? Humanit Soc Sci Commun. 2022:9(1):187. 10.1057/s41599-022-01213-5

[kiaf410-B59] Gray P, Meek S, Griffiths P, Trapani J, Small I, Vickers C, Wood R. Synthetic biology in Australia: an outlook to 2030. Melbourne (Victoria): Australian Council of Learned Academies; 2018. https://acola.org/hs3-synthetic-biology-australia/

[kiaf410-B60] Griscom BW, Adams J, Ellis PW, Houghton RA, Lomax G, Miteva DA, Schlesinger WH, Shoch D, Siikamäki JV, Smith P, et al Natural climate solutions. Proc Natl Acad Sci. 2017:114(44):11645–11650. 10.1073/pnas.1710465114 29078344 PMC5676916

[kiaf410-B61] Guilford WJ, Schneider DM, Labovitz J, Opella SJ. High resolution solid state 13C NMR spectroscopy of sporopollenins from different plant taxa. Plant Physiol. 1988:86(1):134–136. 10.1104/pp.86.1.134 16665854 PMC1054442

[kiaf410-B62] Guo K, Yang J, Yu N, Luo L, Wang E. Biological nitrogen fixation in cereal crops: progress, strategies, and perspectives. Plant Commun. 2023:4(2):100499. 10.1016/j.xplc.2022.100499 36447432 PMC10030364

[kiaf410-B63] Harsono SS, Grundman P, Lau LH, Hansen A, Salleh MAM, Meyer-Aurich A, Idris A, Ghazi TIM. Energy balances, greenhouse gas emissions and economics of biochar production from palm oil empty fruit bunches. Resour Conserv Recycl. 2013:77:108–115. 10.1016/j.resconrec.2013.04.005

[kiaf410-B64] Haskett TL, Paramasivan P, Mendes MD, Green P, Geddes BA, Knights HE, Jorrin B, Ryu M-H, Brett P, Voigt CA, et al Engineered plant control of associative nitrogen fixation. Proc Natl Acad Sci. 2022:119(16):e2117465119. 10.1073/pnas.2117465119 35412890 PMC9169844

[kiaf410-B65] Hassan MU, Aamer M, Mahmood A, Awan MI, Barbanti L, Seleiman MF, Bakhsh G, Alkharabsheh HM, Babur E, Shao J, et al Management strategies to mitigate N_2_O emissions in agriculture. Life (Basel). 2022:12:439. 10.3390/life12030439 35330190 PMC8949344

[kiaf410-B66] Heredia MC, Kant J, Prodhan MA, Dixit S, Wissuwa M. Breeding rice for a changing climate by improving adaptations to water saving technologies. Theor Appl Genet. 2022:135(1):17–33. 10.1007/s00122-021-03899-8 34218290

[kiaf410-B67] Hosseiniyan Khatibi SM, Dimaano NG, Veliz E, Sundaresan V, Ali J. Exploring and exploiting the rice phytobiome to tackle climate change challenges. Plant Commun. 2024:5(12):101078. 10.1016/j.xplc.2024.101078 39233440 PMC11671768

[kiaf410-B68] Howell KR, Shrestha P, Dodd IC. Alternate wetting and drying irrigation maintained rice yields despite half the irrigation volume, but is currently unlikely to be adopted by smallholder lowland rice farmers in Nepal. Food Energy Secur. 2015:4(2):144–157. 10.1002/fes3.58 27610231 PMC4998133

[kiaf410-B69] Huang AC, Osbourn A. Plant terpenes that mediate below-ground interactions: prospects for bioengineering terpenoids for plant protection. Pest Manag Sci. 2019:75(9):2368–2377. 10.1002/ps.5410 30884099 PMC6690754

[kiaf410-B70] Huisman R, Geurts R. A roadmap toward engineered nitrogen-fixing nodule symbiosis. Plant Commun. 2020:1(1):100019. 10.1016/j.xplc.2019.100019 33404552 PMC7748023

[kiaf410-B71] Hunt JM, Philp RP, Kvenvolden KA. Early developments in petroleum geochemistry. Org Geochem. 2002:33(9):1025–1052. 10.1016/S0146-6380(02)00056-6

[kiaf410-B72] IPCC . Climate change and land: an IPCC special report on climate change, desertification, land degradation, sustainable land management, food security, and greenhouse gas fluxes in terrestrial ecosystems. Geneva (Switzerland): International Panel on Climate Change; 2019. https://www.ipcc.ch/srccl/#

[kiaf410-B73] IPCC . Climate change 2021: the physical science basis. In: Contribution of working group I to the sixth assessment report of the Intergovernmental Panel on Climate Change. Geneva (Switzerland): Intergovernmental Panel on Climate Change, CU Press; 2021. p. 1–2409. https://www.ipcc.ch/report/ar6/wg1/

[kiaf410-B74] IPCC . Climate change 2022: mitigation of climate change. In: Contribution of working group III to the sixth assessment report. Geneva (Switzerland): Intergovernmental Panel on Climate Change, CU Press; 2022a. p. 1–2029. https://www.ipcc.ch/report/ar6/wg3/

[kiaf410-B75] IPCC . Chapter 3: Mitigation pathways compatible with long-term goals. In: Climate change 2022: mitigation of climate change. Contribution of working group III to the sixth assessment report of the intergovernmental panel on climate change. Geneva (Switzerland): Intergovernmental Panel on Climate Change; 2022b. p. 295–408. https://www.ipcc.ch/report/ar6/wg3/chapter/chapter-3/

[kiaf410-B76] IPCC . Climate change 2023: Synthesis report. In: Contribution of working groups I, II and III to the sixth assessment report of the Intergovernmental Panel on Climate Change. Geneva (Switzerland): Intergovernmental Panel on Climate Change; 2023. p. 1–115 https://www.ipcc.ch/report/sixth-assessment-report-cycle/.

[kiaf410-B77] IRENA . Global energy transformation: a roadmap to 2050. 2019 ed. Abu Dhabi: International Renewable Energy Agency; 2019. https://www.irena.org/apps/DigitalArticles/-/media/652AE07BBAAC407ABD1D45F6BBA8494B.ashx

[kiaf410-B78] Irvine PJ, Kravitz B, Lawrence MG, Muri H. An overview of the earth system science of solar geoengineering. Wiley Interdiscip Rev Clim Change. 2016:7:815–833. 10.1002/wcc.423

[kiaf410-B79] Irvine PJ, Ridgwell A, Lunt DJ. Climatic effects of surface albedo geoengineering. J Geophys Res. 2011:116:24112. 10.1029/2011JD016281

[kiaf410-B80] ISO . ISO 14040:2006/Amd 1:2020 environmental management—life cycle assessment—principles and framework. Geneva (Switzerland): International Organization for Standardization; 2020. https://www.iso.org/standard/76121.html

[kiaf410-B81] IWGIA . Land grabbing, investments & indigenous peoples’ rights to land and natural resources. Copenhagen (Denmark): International Work Group for Indigenous Affairs; 2015. https://iwgia.org/images/publications/new-publications/land-grabbing-indigenous-peoples-rights.compressed.pdf

[kiaf410-B82] Jackson DA, Symons RH, Berg P. Biochemical method for inserting new genetic information into DNA of Simian Virus 40: circular SV40 DNA molecules containing lambda phage genes and the galactose operon of *Escherichia coli*. Proc Natl Acad Sci. 1972:69(10):2904–2909. 10.1073/pnas.69.10.2904 4342968 PMC389671

[kiaf410-B83] Jardine KJ, Gallo L, Roth M, Upadhyaya S, Northen T, Kosina S, Tcherkez G, Eudes A, Domigues T, Greule M, et al The ‘photosynthetic C1 pathway’ links carbon assimilation and growth in California poplar. Commun Biol. 2024:7(1):1469. 10.1038/s42003-024-07142-0 39516667 PMC11549359

[kiaf410-B84] Jez JM, Lee SG, Sherp AM. The next green movement: plant biology for the environment and sustainability. Science. 2016:353(6305):1241–1244. 10.1126/science.aag1698 27634525

[kiaf410-B85] Jhu M-Y, Oldroyd GED. Dancing to a different tune, can we switch from chemical to biological nitrogen fixation for sustainable food security? PLoS Biol. 2023:21(3):e3001982. 10.1371/journal.pbio.3001982 36917569 PMC10013914

[kiaf410-B86] Jia X, Liu P, Lynch JP. Greater lateral root branching density in maize improves phosphorus acquisition from low phosphorus soil. J Exp Bot. 2018:69(20):4961–4970. 10.1093/jxb/ery252 30295904 PMC6137997

[kiaf410-B87] Johnston E, Okada S, Gregg CM, Warden AC, Rolland V, Gillespie V, Byrne K, Colgrave ML, Eamens AL, Allen RS, et al The structural components of the Azotobacter vinelandii iron-only nitrogenase, AnfDKG, form a protein complex within the plant mitochondrial matrix. Plant Mol Biol. 2023:112(4–5):279–291. 10.1007/s11103-023-01363-3 37326800 PMC10352409

[kiaf410-B88] Jones MW, Peters GP, Gasser T, Andrew RM, Schwingshackl C, Gütschow J, Houghton RA, Friedlingstein P, Pongratz J, Le Quéré C. National contributions to climate change due to historical emissions of carbon dioxide, methane and nitrous oxide. Brussels (Belgium): European Commission, Zenodo: the EU Open Research Repository; 2023a. 10.5281/zenodo.7076346 PMC10060593 36991071

[kiaf410-B89] Jones MW, Peters GP, Gasser T, Andrew RM, Schwingshackl C, Gütschow J, Houghton RA, Friedlingstein P, Pongratz J, Le Quéré C. National contributions to climate change due to historical emissions of carbon dioxide, methane, and nitrous oxide since 1850. Sci Data. 2023b:10(1):155. 10.1038/s41597-023-02041-1 36991071 PMC10060593

[kiaf410-B90] Joshi J, Amthor JS, McCarty DR, Messina CD, Wilson MA, Millar AH, Hanson AD. Why cutting respiratory CO2 loss from crops is possible, practicable, and prudential. Modern Agriculture. 2023:1(1):16–26. 10.1002/moda.1

[kiaf410-B91] Kassaw TK, Xu W, Zalewski CS, Kiwimagi K, Weiss R, Antunes MS, Prasad A, Medford JI. Genetic toggle switch in plants. ACS Synth Bio. 2025:14(6):1988–2001. 40387045 10.1021/acssynbio.4c00777

[kiaf410-B92] Khaipho-Burch M, Cooper M, Crossa J, Nd L, Holland J, Lewis R, McCouch S, Murray SC, Rabbi I, Ronald P, et al Genetic modification can improve crop yields—but stop overselling it. Nature. 2023:621(7979):470–473. 10.1038/d41586-023-02895-w 37773222 PMC11550184

[kiaf410-B93] Khakhar A, Leydon AR, Lemmex AC, Klavins E, Nemhauser JL. Synthetic hormone-responsive transcription factors can monitor and re-program plant development. eLife. 2018:7:e34702. 10.7554/eLife.34702 29714687 PMC5976440

[kiaf410-B94] Khanday I, Skinner D, Yang B, Mercier R, Sundaresan V. A male-expressed rice embryogenic trigger redirected for asexual propagation through seeds. Nature. 2019:565(7737):91–95. 10.1038/s41586-018-0785-8 30542157

[kiaf410-B95] Kitaoka N, Lu X, Yang B, Peters RJ. The application of synthetic biology to elucidation of plant mono-, sesqui-, and diterpenoid metabolism. Mol Plant. 2015:8(1):6–16. 10.1016/j.molp.2014.12.002 25578268 PMC5120878

[kiaf410-B96] Kobos PH, Drennen TE, Outkin AV, Webb EK, Paap SM, Wiryadinata S, Sandia National Laboratories. Techno-economic analysis: best practices and assessment tools. Livermore (CA): Sandia National Lab. (SNL-CA); Albuquerque (NM): Sandia National Lab. (SNL-NM); Geneva (NY): Hobart and William Smith Colleges; 2020. https://www.osti.gov/biblio/1738878

[kiaf410-B97] Kocaoglan EG, Radhakrishnan D, Nakayama N. Synthetic developmental biology: molecular tools to re-design plant shoots and roots. J Exp Bot. 2023:74(13):3864–3876. 10.1093/jxb/erad169 37155965 PMC10826796

[kiaf410-B98] Kong C, Yang Y, Qi T, Zhang S. Predictive genetic circuit design for phenotype reprogramming in plants. Nat Commun. 2025:16(1):715. 10.1038/s41467-025-56042-2 39820378 PMC11739397

[kiaf410-B99] Kozaeva E, Eida AA, Gunady EF, Dangl JL, Conway JM, Brophy JAN. Roots of synthetic ecology: microbes that foster plant resilience in the changing climate. Curr Opin Biotechnol. 2024:88:103172. 10.1016/j.copbio.2024.103172 39029405

[kiaf410-B100] Kubis A, Bar-Even A. Synthetic biology approaches for improving photosynthesis. J Exp Bot. 2019:70(5):1425–1433. 10.1093/jxb/erz029 30715460 PMC6432428

[kiaf410-B101] Kwok R . Five hard truths for synthetic biology. Nature. 2010:463(7279):288–290. 10.1038/463288a 20090726

[kiaf410-B102] Kwon Y, Jin Y, Lee J-H, Sun C, Ryu C-M. Rice rhizobiome engineering for climate change mitigation. Trends Plant Sci. 2024:29(12):1299–1309. 10.1016/j.tplants.2024.06.006 39019767

[kiaf410-B103] Lai J, Kooijmans LMJ, Sun W, Lombardozzi D, Campbell JE, Gu L, Luo Y, Kuai L, Sun Y. Terrestrial photosynthesis inferred from plant carbonyl sulfide uptake. Nature. 2024:634(8035):855–861. 10.1038/s41586-024-08050-3 39415019

[kiaf410-B104] Lark TJ, Hendricks NP, Smith A, Pates N, Spawn-Lee SA, Bougie M, Booth EG, Kucharik CJ, Gibbs HK. Environmental outcomes of the US Renewable Fuel Standard. Proc Natl Acad Sci. 2022:119(9):e2101084119. 10.1073/pnas.2101084119 35165202 PMC8892349

[kiaf410-B105] Lehmann J, Joseph S. Biochar for environmental management: science, technology and implementation. 3rd ed. London: Taylor & Francis; 2024.

[kiaf410-B106] Lehne J, Preston F. Making concrete change: innovation in low-carbon cement and concrete. London (UK): Chatham House, The Royal Institute of International Affairs; 2018. https://www.chathamhouse.org/2018/06/making-concrete-change-innovation-low-carbon-cement-and-concrete

[kiaf410-B107] Leon A, Izumi T. Impacts of alternate wetting and drying on rice farmers’ profits and life cycle greenhouse gas emissions in An Giang Province in Vietnam. J Clean Prod. 2022:354:131621. 10.1016/j.jclepro.2022.131621

[kiaf410-B108] Lew TTS, Sarojam R, Jang IC, Park BS, Naqvi NI, Wong MH, Singh GP, Ram RJ, Shoseyov O, Saito K, et al Species-independent analytical tools for next-generation agriculture. Nat Plants. 2020:6(12):1408–1417. 10.1038/s41477-020-00808-7 33257857

[kiaf410-B109] Lewandowski I, Clifton-Brown J, Trindade LM, van der Linden GC, Schwarz KU, Muller-Samann K, Anisimov A, Chen CL, Dolstra O, Donnison IS, et al Progress on optimizing miscanthus biomass production for the European bioeconomy: results of the EU FP7 project OPTIMISC. Front Plant Sci. 2016:7:1620. 10.3389/fpls.2016.01620 27917177 PMC5114296

[kiaf410-B110] Li F-S, Phyo P, Jacobowitz J, Hong M, Weng J-K. The molecular structure of plant sporopollenin. Nat Plants. 2019:5(1):41–46. 10.1038/s41477-018-0330-7 30559416

[kiaf410-B111] Linden A, Fenn J. Understanding Gartner's Hype cycles. New York (NY): Gartner Research; 2003. http://ask-force.org/web/Discourse/Linden-HypeCycle-2003.pdf

[kiaf410-B112] Linquist BA, van Groenigen KJ, Adviento-Borbe MA, Pittelkow CM, van Kessel C. An agronomic assessment of greenhouse gas emissions from major cereal crops. Glob Change Biol. 2012:18(1):194–209. 10.1111/j.1365-2486.2011.02502.x

[kiaf410-B113] Liu D, He J, Li Q, Zhang X, Wang Y, Sun Q, Wang W, Zhang M, Wang Y, Xu H, et al A WRKY transcription factor confers broad-spectrum resistance to biotic stresses and yield stability in rice. Proc Natl Acad Sci. 2025:122(10):e2411164122. 10.1073/pnas.2411164122 40042898 PMC11912400

[kiaf410-B114] Liu Y, Yuan G, Hassan MM, Abraham PE, Mitchell JC, Jacobson D, Tuskan GA, Khakhar A, Medford J, Zhao C, et al Biological and molecular components for genetically engineering biosensors in plants. Biodes Res. 2022:2022:9863496. 10.34133/2022/9863496 37850147 PMC10521658

[kiaf410-B115] Living Carbon Team . Photosynthesis enhanced trees grow faster and capture more carbon. 2022 [accessed 2025 Mar 22]. https://www.livingcarbon.com/post/photosynthesis-enhanced-trees-grow-faster-and-capture-more-carbon

[kiaf410-B116] Lloyd JPB, Ly F, Gong P, Pflueger J, Swain T, Pflueger C, Fourie E, Khan MA, Kidd BN, Lister R. Synthetic memory circuits for stable cell reprogramming in plants. Nat Biotechnol. 2022:40(12):1862–1872. 10.1038/s41587-022-01383-2 35788565

[kiaf410-B117] Lovell-Read FA, Parnell S, Cunniffe NJ, Thompson RN. Using ‘sentinel’ plants to improve early detection of invasive plant pathogens. PLoS Comput Biol. 2023:19(2):e1010884. 10.1371/journal.pcbi.1010884 36730434 PMC9928126

[kiaf410-B118] Luo L, Mei H, Yu X, Xia H, Chen L, Liu H, Zhang A, Xu K, Wei H, Liu G, et al Water-saving and drought-resistance rice: from the concept to practice and theory. Mol Breed. 2019:39(10–11):145. 10.1007/s11032-019-1057-5

[kiaf410-B119] Lyons TW, Reinhard CT, Planavsky NJ. The rise of oxygen in Earth's early ocean and atmosphere. Nature. 2014:506(7488):307–315. 10.1038/nature13068 24553238

[kiaf410-B120] Mackenzie G, Boa AN, Diego-Taboada A, Atkin SL, Sathyapalan T. Sporopollenin, The least known yet toughest natural biopolymer. Front Mater. 2015:2:66. 10.3389/fmats.2015.00066

[kiaf410-B121] MacMartin DG, Ricke KL, Keith DW. Solar geoengineering as part of an overall strategy for meeting the 1.5 C Paris target. Philos Trans R Soc A: Math Phys Eng Sci. 2018:376(2119):20160454. 10.1098/rsta.2016.0454 PMC5897825 29610384

[kiaf410-B122] Mankad A, Hobman EV, Carter L. Effects of knowledge and emotion on support for novel synthetic biology applications. Conserv Biol. 2021:35(2):623–633. 10.1111/cobi.13637 33022794

[kiaf410-B123] Martinez-Feria R, Simmonds MB, Ozaydin B, Lewis S, Schwartz A, Pluchino A, McKellar M, Gottlieb SS, Kayatsky T, Vital R, et al Genetic remodeling of soil diazotrophs enables partial replacement of synthetic nitrogen fertilizer with biological nitrogen fixation in maize. Sci Rep. 2024:14:27754. 10.1038/s41598-024-78243-3 39532958 PMC11557888

[kiaf410-B124] Maruthi YA, Ramakrishna S. Sporopollenin—invincible biopolymer for sustainable biomedical applications. Int J Biol Macromol. 2022:222:2957–2965. 10.1016/j.ijbiomac.2022.10.071 36244536

[kiaf410-B125] McCalmont JP, Hastings A, McNamara NP, Richter GM, Robson P, Donnison IS, Clifton-Brown J. Environmental costs and benefits of growing Miscanthus for bioenergy in the UK. Glob Change Biol Bioenergy. 2017:9(3):489–507. 10.1111/gcbb.12294 28331551 PMC5340280

[kiaf410-B126] McGurrin A, Maguire J, Tiwari BK, Garcia-Vaquero M. Anti-methanogenic potential of seaweeds and seaweed-derived compounds in ruminant feed: current perspectives, risks and future prospects. J Anim Sci Biotechnol. 2023:14(1):145. 10.1186/s40104-023-00946-w 38041152 PMC10693045

[kiaf410-B127] Medford J, Morey K, Kassaw T, Antunes M. Synthetic desalination genetic circuit in plants. US11692199B2. Alexandria (VA): US Patent Office; 2023.

[kiaf410-B128] Mendez-Millan M, Dignac MF, Rumpel C, Rasse DP, Derenne S. Molecular dynamics of shoot vs. root biomarkers in an agricultural soil estimated by natural abundance 13C labelling. Soil Biol Biochem. 2010:42(2):169–177. 10.1016/j.soilbio.2009.10.010

[kiaf410-B129] Michaux SP . Estimation of the quantity of metals to phase out fossil fuels in a full system replacement, compared to mineral resources. Geol Surv Finl Bull Special Issue. 2024:416:1–293. https://media.voog.com/0000/0051/5165/files/bt_416.pdf

[kiaf410-B130] Millar N, Robertson GP, Grace PR, Gehl RJ, Hoben JP. Nitrogen fertilizer management for nitrous oxide (N_2_O) mitigation in intensive corn (Maize) production: an emissions reduction protocol for US Midwest agriculture. Mitig Adapt Strateg Glob Change. 2010:15(2):185–204. 10.1007/s11027-010-9212-7

[kiaf410-B131] Mills L . Diamonds are expensive: lessons learned about applying synthetic biology for 1000+ year carbon dioxide removal. Hayward (CA): Living Carbon; 2023. https://www.livingcarbon.com/post/diamonds-are-expensive-lessons-learned-about-applying-synthetic-biology-for-1000-year-carbon-dioxide-removal

[kiaf410-B132] Mitchell D . A note on rising food prices. Washington (DC): World Bank; 2008. https://hdl.handle.net/10986/6820

[kiaf410-B133] Morey K, Khakhar A. Exploring the frontier of rapid prototyping technologies for plant synthetic biology and what could lie beyond. New Phytol. 2024:242(3):903–908. 10.1111/nph.19650 38426415

[kiaf410-B134] Nakabayashi R, Saito K. Higher dimensional metabolomics using stable isotope labeling for identifying the missing specialized metabolism in plants. Curr Opin Plant Biol. 2020:55:84–92. 10.1016/j.pbi.2020.02.009 32388402

[kiaf410-B135] National Academies of Sciences Engineering, and Medicine . Negative emissions technologies and reliable sequestration: a research agenda. Washington (DC): The National Academies Press; 2019. 10.17226/25259 31120708

[kiaf410-B136] Ng TL, Eheart JW, Cai X, Miguez F. Modeling Miscanthus in the soil and water assessment tool (SWAT) to simulate its water quality effects as a bioenergy crop. Environ Sci Technol. 2010:44(18):7138–7144. 10.1021/es9039677 20681575

[kiaf410-B137] OECD/FAO . OECD-FAO Agricultural outlook 2023–2032. Paris (OECD) and Rome (FAO): Organisation for Economic Cooperation and Development and Food and Agriculture Organisation of the United Nations; 2023. 10.1787/08801ab7-en

[kiaf410-B138] Oliveira-Filho ER, Campos-Silva R, Hanson AD. Running Fermi calculations as a superpower to gauge reality. Plant Physiol. 2025:198:kiae347. 10.1093/plphys/kiae347 38876095

[kiaf410-B139] Ontl TA, Schulte LA. Soil carbon storage. Nat Educ Knowl. 2012:3:35. https://www.nature.com/scitable/knowledge/library/soil-carbon-storage-84223790/

[kiaf410-B140] Organization UNEPaFaA . Global nitrous oxide assessment. Nairobi: Food and Agriculture Organization of the United Nations, FaAOotU Nations; 2024. 10.59117/20.500.11822/46562

[kiaf410-B141] Owen C, Patron NJ, Huang A, Osbourn A. Harnessing plant metabolic diversity. Curr Opin Chem Biol. 2017:40:24–30. 10.1016/j.cbpa.2017.04.015 28527344 PMC5693780

[kiaf410-B142] Pan Y, Birdsey RA, Fang J, Houghton R, Kauppi PE, Kurz WA, Phillips OL, Shvidenko A, Lewis SL, Canadell JG, et al A large and persistent carbon sink in the world's forests. Science. 2011:333(6045):988–993. 10.1126/science.1201609 21764754

[kiaf410-B143] Patel DP, Das A, Munda GC, Ghosh PK, Bordoloi JS, Kumar M. Evaluation of yield and physiological attributes of high-yielding rice varieties under aerobic and flood-irrigated management practices in mid-hills ecosystem. Agric Water Manag. 2010:97(9):1269–1276. 10.1016/j.agwat.2010.02.018

[kiaf410-B144] Patel MR, Panwar NL. Development, process optimization and assessment of sustainable mobile biochar kiln for agricultural use. J Clean Prod. 2024:477:143866. 10.1016/j.jclepro.2024.143866

[kiaf410-B145] Patron NJ . Beyond natural: synthetic expansions of botanical form and function. New Phytol. 2020:227(2):295–310. 10.1111/nph.16562 32239523 PMC7383487

[kiaf410-B146] Paustian K, Lehmann J, Ogle S, Reay D, Robertson GP, Smith P. Climate-smart soils. Nature. 2016:532(7597):49–57. 10.1038/nature17174 27078564

[kiaf410-B147] Peters JF, Iribarren D, Dufour J. Biomass pyrolysis for biochar or energy applications? A life cycle assessment. Environ Sci Technol. 2015:49(8):5195–5202. 10.1021/es5060786 25830564

[kiaf410-B148] Poddar TK, Scown CD. Technoeconomic analysis for near-term scale-up of bioprocesses. Curr Opin Biotechnol. 2025:92:103258. 10.1016/j.copbio.2025.103258 39837197

[kiaf410-B149] Poore J, Nemecek T. Reducing food's environmental impacts through producers and consumers. Science. 2018:360(6392):987–992. 10.1126/science.aaq0216 29853680

[kiaf410-B150] Pouvreau B, Vanhercke T, Singh S. From plant metabolic engineering to plant synthetic biology: the evolution of the design/build/test/learn cycle. Plant Sci. 2018:273:3–12. 10.1016/j.plantsci.2018.03.035 29907306

[kiaf410-B151] Prywes N, Phillips NR, Oltrogge LM, Lindner S, Taylor-Kearney LJ, Tsai Y-CC, de Pins B, Cowan AE, Chang HA, Wang RZ, et al A map of the rubisco biochemical landscape. Nature. 2025:638(8051):823–828. 10.1038/s41586-024-08455-0 39843747 PMC11839469

[kiaf410-B152] Qin K, Ye X, Luo S, Fernie AR, Zhang Y. Engineering carbon assimilation in plants. J Integr Plant Biol. 2025:67:926–948. 10.1111/jipb.13825 39783795

[kiaf410-B153] Rabin KR . UF researcher investigating ‘plant diamond’ as a carbon removal powerhouse. 2023 [accessed 2025 Mar 20]. https://blogs.ifas.ufl.edu/news/2023/12/04/energy-earthshot-kirst/

[kiaf410-B154] Rhee SY, Anstett DN, Cahoon EB, Covarrubias-Robles AA, Danquah E, Dudareva N, Ezura H, Gilbert KJ, Gutiérrez RA, Heck M, et al Resilient plants, sustainable future. Trends Plant Sci. 2025:30:382–388. 10.1016/j.tplants.2024.11.001 39643496

[kiaf410-B155] Rim EY, Garrett OD, Howard AJ, Shim Y, Li Y, Van Dyke JE, Packer RC, Ho N, Jain R, Stewart V, et al Directed evolution of a plant immune receptor for broad spectrum effector recognition. bioRxiv 2024.09.30.614878. 10.1101/2024.09.30.614878 01 October 2024; preprint: not peer reviewed.

[kiaf410-B156] Ritchie H, Rosado P, Roser M. Sector by sector: where do global greenhouse gas emissions come from? 2020 [accessed 2025 March 25]. https://ourworldindata.org/ghg-emissions-by-sector

[kiaf410-B157] Ritchie H, Rosado P, Roser M. Meat and dairy production. Our World in Data; 2023 [accessed 2025 Mar 18]. https://ourworldindata.org/meat-production

[kiaf410-B158] Rogers C, Oldroyd GED. Synthetic biology approaches to engineering the nitrogen symbiosis in cereals. J Exp Bot. 2014:65(8):1939–1946. 10.1093/jxb/eru098 24687978

[kiaf410-B159] Rosenzweig C, Mbow C, Barioni LG, Benton TG, Herrero M, Krishnapillai M, Liwenga ET, Pradhan P, Rivera-Ferre MG, Sapkota T, et al Climate change responses benefit from a global food system approach. Nat Food. 2020:1(2):94–97. 10.1038/s43016-020-0031-z 37128000

[kiaf410-B160] Royal Society . Ammonia: zero-carbon fertiliser, fuel and energy store: policy briefing. London (UK): Royal Society; 2020.

[kiaf410-B161] Ryu MH, Zhang J, Toth T, Khokhani D, Geddes BA, Mus F, Garcia-Costas A, Peters JW, Poole PS, Ané JM, et al Control of nitrogen fixation in bacteria that associate with cereals. Nat Microbiol. 2020:5(2):314–330. 10.1038/s41564-019-0631-2 31844298 PMC8634771

[kiaf410-B162] Sahoo K, Upadhyay A, Runge T, Bergman R, Puettmann M, Bilek E. Life-cycle assessment and techno-economic analysis of biochar produced from forest residues using portable systems. Int J Life Cycle Assess. 2021:26(1):189–213. 10.1007/s11367-020-01830-9

[kiaf410-B163] Sainsbury F, Lomonossoff GP. Transient expressions of synthetic biology in plants. Curr Opin Plant Biol. 2014:19:1–7. 10.1016/j.pbi.2014.02.003 24631883 PMC4070481

[kiaf410-B164] Sanei H, Rudra A, Przyswitt ZMM, Kousted S, Sindlev MB, Zheng X, Nielsen SB, Petersen HI. Assessing biochar's permanence: an inertinite benchmark. Int J Coal Geol. 2024:281:104409. 10.1016/j.coal.2023.104409

[kiaf410-B165] Schaffer S, Pröll T, Al Afif R, Pfeifer C. A mass- and energy balance-based process modelling study for the pyrolysis of cotton stalks with char utilization for sustainable soil enhancement and carbon storage. Biomass Bioenergy. 2019:120:281–290. 10.1016/j.biombioe.2018.11.019

[kiaf410-B166] Schmidt HP, Bucheli T, Kammann C, Glaser B, Abiven S, Leifeld J, Soja G, Hagemann N. European Biochar certificate—guidelines for a sustainable production of biochar 2021–2024. Frick, Switzerland: Carbon Standards International (CSI); 2024.

[kiaf410-B167] Schramski JR, Gattie DK, Brown JH. Human domination of the biosphere: rapid discharge of the earth-space battery foretells the future of humankind. Proc Natl Acad Sci. 2015:112(31):9511–9517. 10.1073/pnas.1508353112 26178196 PMC4534254

[kiaf410-B168] Schulz-Mirbach H, Wichmann P, Satanowski A, Meusel H, Wu T, Nattermann M, Burgener S, Paczia N, Bar-Even A, Erb TJ. New-to-nature CO_2_-dependent acetyl-CoA assimilation enabled by an engineered B_12_-dependent acyl-CoA mutase. Nat Commun. 2024:15(1):10235. 10.1038/s41467-024-53762-9 39592584 PMC11599936

[kiaf410-B169] Sciarresi C, Thies A, Topp C, Eudy D, Kovar JL, Trifunovic S, Dixon PM, Archontoulis SV. Breeding for high maize yields indirectly boosting root carbon in the US corn belt since the 1980s. Field Crops Res. 2025:323:109774. 10.1016/j.fcr.2025.109774

[kiaf410-B170] Sen S . Geoengineering cities with reflective and pervious surfaces. In: Geoengineering and climate change. Beverly (MA): Scrivener Publishing LLC; 2025. p. 231–245. 10.1002/9781394204847.ch14

[kiaf410-B171] Sha G, Sun P, Kong X, Han X, Sun Q, Fouillen L, Zhao J, Li Y, Yang L, Wang Y, et al Genome editing of a rice CDP-DAG synthase confers multipathogen resistance. Nature. 2023:618(7967):1017–1023. 10.1038/s41586-023-06205-2 37316672 PMC11575942

[kiaf410-B172] Shaw G . The chemistry of sporopollenin. In: Brooks J, Grant PR, Muir M, Van Gijzel P, Shaw G, editors. Sporopollenin. Cambridge (MA): Academic Press; 1971. p. 305–350. 10.1016/B978-0-12-135750-4.50017-1

[kiaf410-B173] Shepherd JG . Geoengineering the climate: science, governance and uncertainty. London (UK): The Royal Society; 2009. https://royalsociety.org/news-resources/publications/2009/geoengineering-climate/

[kiaf410-B174] Shi L-D, Ercoli MF, Kim J, de Araujo Junior AT, Soni S, Weitz TS, Shigenaga AM, Dukovski I, Sachdeva R, Turumtay H, et al Reduced methane emissions in transgenic rice genotypes are associated with altered rhizosphere microbial hydrogen cycling. bioRxiv 2024.10.07.617079. 10.1101/2024.10.07.617079, 07 October 2024, preprint: not peer reviewed PMC12946211 41582156

[kiaf410-B175] Skrzypczak D, Gorazda K, Mikula K, Mironiuk M, Kominko H, Sawska K, Evrard D, Trzaska K, Moustakas K, Chojnacka K. Towards carbon neutrality: enhancing CO2 sequestration by plants to reduce carbon footprint. Sci Total Environ. 2025:966:178763. 10.1016/j.scitotenv.2025.178763 39922011

[kiaf410-B176] Smith P, Reay D, Smith J. Agricultural methane emissions and the potential formitigation. Philos Trans A Math Phys Eng Sci. 2021:379(2210):20200451. 10.1098/rsta.2020.0451 34565225

[kiaf410-B177] Sohi SP, Krull E, Lopez-Capel E, Bol R. A review of biochar and its use and function in soil. In: Advances in agronomy. 105. Cambridge (MA): Academic Press; 2010. p. 47–82.

[kiaf410-B179] South PF, Cavanagh AP, Liu HW, Ort DR. Synthetic glycolate metabolism pathways stimulate crop growth and productivity in the field. Science. 2019a:363(6422):eaat9077. 10.1126/science.aat9077 30606819 PMC7745124

[kiaf410-B178] South PF, Cavanagh AP, Liu HW, Ort DR. Erratum for the Research Article “Synthetic glycolate metabolism pathways stimulate crop growth and productivity in the field” by P. F. South, A. P. Cavanagh, H. W. Liu, D. R. Ort. Science. 2019b:365(6452):eaay8818. 10.1126/science.aay8818 31371580

[kiaf410-B180] Summons RE, Lincoln SA. Biomarkers: informative molecules for studies in geobiology. In: Fundamentals of geobiology. Oxford (UK): Blackwell Publishing Ltd; 2012. p. 269–296. 10.1002/9781118280874.ch15

[kiaf410-B181] Tao Y, Chiu L-W, Hoyle JW, Dewhirst RA, Richey C, Rasmussen K, Du J, Mellor P, Kuiper J, Tucker D, et al Enhanced photosynthetic efficiency for increased carbon assimilation and woody biomass production in engineered hybrid poplar. Forests. 2023:14(4):827. 10.3390/f14040827

[kiaf410-B182] Tian H, Pan N, Thompson RL, Canadell JG, Suntharalingam P, Regnier P, Davidson EA, Prather M, Ciais P, Muntean M, et al Global nitrous oxide budget (1980–2020). Earth Syst Sci Data. 2024:16:2543–2604. 10.5194/essd-16-2543-2024

[kiaf410-B183] Tiedge K, Muchlinski A, Zerbe P. Genomics-enabled analysis of specialized metabolism in bioenergy crops: current progress and challenges. Synth Biol (Oxf). 2020:5(1):ysaa005. 10.1093/synbio/ysaa005 32995549 PMC7445794

[kiaf410-B184] United Nations Department of Economic and Social Affairs, Population Division . World population prospects 2024: summary of results. New York (NY): United Nations; 2024. https://desapublications.un.org/publications/world-population-prospects-2024-summary-results?_gl=1*15ag4t0*_ga*MzIzNDcxMzI5LjE3NDg2NzUwMzY.*_ga_TK9BQL5X7Z*czE3NDg2OTYwMTQkbzIkZzAkdDE3NDg2OTYwMTQkajYwJGwwJGgw

[kiaf410-B185] Ursache R, De Jesus Vieira Teixeira C, Dénervaud Tendon V, Gully K, De Bellis D, Schmid-Siegert E, Grube Andersen T, Shekhar V, Calderon S, Pradervand S, et al GDSL-domain proteins have key roles in suberin polymerization and degradation. Nat Plants. 2021:7(3):353–364. 10.1038/s41477-021-00862-9 33686223 PMC7610369

[kiaf410-B186] Van Deynze A, Zamora P, Delaux P-M, Heitmann C, Jayaraman D, Rajasekar S, Graham D, Maeda J, Gibson D, Schwartz KD, et al Nitrogen fixation in a landrace of maize is supported by a mucilage-associated diazotrophic microbiota. PLoS Biol. 2018:16(8):e2006352. 10.1371/journal.pbio.2006352 30086128 PMC6080747

[kiaf410-B187] Van Gelder K, Oliveira-Filho ER, Messina CD, Venado RE, Wilker J, Rajasekar S, Ane JM, Amthor JS, Hanson AD. Running the numbers on plant synthetic biology solutions to global problems. Plant Sci. 2023:335:111815. 10.1016/j.plantsci.2023.111815 37543223

[kiaf410-B188] Vavitsas K, Crozet P, Vinde MH, Davies F, Lemaire SD, Vickers CE. The synthetic biology toolkit for photosynthetic microorganisms. Plant Physiol. 2019:181(1):14–27. 10.1104/pp.19.00345 31262955 PMC6716251

[kiaf410-B189] Vazquez-Vilar M, Selma S, Orzaez D. The design of synthetic gene circuits in plants: new components, old challenges. J Exp Bot. 2023:74(13):3791–3805. 10.1093/jxb/erad167 37204924 PMC10353530

[kiaf410-B190] Venado RE, Wilker J, Pankievicz VCS, Infante V, MacIntyre A, Wolf ESA, Vela S, Robbins F, Fernandes-Júnior PI, Vermerris W, et al Mucilage produced by aerial roots hosts diazotrophs that provide nitrogen in Sorghum bicolor. PLoS Biol. 2025:23(3):e3003037. 10.1371/journal.pbio.3003037 40029899 PMC12136154

[kiaf410-B191] Venkataraman M, Yñigez-Gutierrez A, Infante V, MacIntyre A, Fernandes-Júnior PI, Ané J-M, Pfleger B. Synthetic biology toolbox for nitrogen-fixing soil microbes. ACS Synth Biol. 2023:12(12):3623–3634. 10.1021/acssynbio.3c00414 37988619 PMC10754042

[kiaf410-B192] Verheijen F, Jeffery S, Bastos AC, van der Velde M, Diafas I. Biochar application to soils: a critical scientific review of effects on soil properties, processes and functions. Luxembourg: Office for the Official Publications of the European Communities; 2010. https://publications.jrc.ec.europa.eu/repository/handle/JRC57810

[kiaf410-B193] Vicente L, Peña D, Fernández D, Albarrán Á, Rato-Nunes JM, López-Piñeiro A. Alternate wetting and drying irrigation with field aged biochar may enhance water and rice productivity. Agron Sustain Dev. 2025:45(1):6. 10.1007/s13593-024-01000-3

[kiaf410-B194] Victoria AJ, Astbury MJ, McCormick AJ. Engineering highly productive cyanobacteria towards carbon negative emissions technologies. Curr Opin Biotechnol. 2024:87:103141. 10.1016/j.copbio.2024.103141 38735193

[kiaf410-B195] Volkman JK . Sterols and other triterpenoids: source specificity and evolution of biosynthetic pathways. Org Geochem. 2005:36(2):139–159. 10.1016/j.orggeochem.2004.06.013

[kiaf410-B196] Wellman CH, Osterloff PL, Mohiuddin U. Fragments of the earliest land plants. Nature. 2003:425(6955):282–285. 10.1038/nature01884 13679913

[kiaf410-B197] Weng JK, Lynch JH, Matos JO, Dudareva N. Adaptive mechanisms of plant specialized metabolism connecting chemistry to function. Nat Chem Biol. 2021:17(10):1037–1045. 10.1038/s41589-021-00822-6 34552220

[kiaf410-B198] Whitehill JGA, Bohlmann J. A molecular and genomic reference system for conifer defence against insects. Plant Cell Environ. 2019:42(10):2844–2859. 10.1111/pce.13571 31042808 PMC6852437

[kiaf410-B199] Wigley TML . A combined mitigation/geoengineering approach to climate stabilization. Science. 2006:314(5798):452–454. 10.1126/science.1131728 16973840

[kiaf410-B200] Wolf J, West TO, Le Page Y, Kyle GP, Zhang X, Collatz GJ, Imhoff ML. Biogenic carbon fluxes from global agricultural production and consumption. Global Biogeochem Cycles. 2015:29(10):1617–1639. 10.1002/2015GB005119

[kiaf410-B201] Woolf D, Amonette JE, Street-Perrott FA, Lehmann J, Joseph S. Sustainable biochar to mitigate global climate change. Nat Commun. 2010:1(1):56. 10.1038/ncomms1053 20975722 PMC2964457

[kiaf410-B202] WRI . Climatewatch: historical GHG emissions. Washington (DC): World Resources Institute; 2025. https://www.climatewatchdata.org/ghg-emissions?apcid=000000000000000000000000&breakBy=sector&chartType=line&end_year=2021&start_year=1990

[kiaf410-B204] WRI & WBCSD . Greenhouse gas protocol: a corporate accounting and reporting standard. New York (NY): WRI; and Geneva (Switzerland): World Resources Institute and World Business Council for Sustainable Development; 2024. https://ghgprotocol.org/

[kiaf410-B203] Wright RC, Nemhauser J. Plant synthetic biology: quantifying the “known unknowns” and discovering the “unknown unknowns”. Plant Physiol. 2019:179(3):885–893. 10.1104/pp.18.01222 30630870 PMC6393784

[kiaf410-B205] Wurtzel ET, Vickers CE, Hanson AD, Millar AH, Cooper M, Voss-Fels KP, Nikel PI, Erb TJ. Revolutionizing agriculture with synthetic biology. Nat Plants. 2019:5(12):1207–1210. 10.1038/s41477-019-0539-0 31740769

[kiaf410-B206] Yang X, Liu D, Lu H, Weston DJ, Chen J-G, Muchero W, Martin S, Liu Y, Hassan MM, Yuan G, et al Biological parts for plant biodesign to enhance land-based carbon dioxide removal. Biodes Res. 2021:2021:9798714. 10.34133/2021/9798714 37849951 PMC10521660

[kiaf410-B207] Yasmin F, Cowie AE, Zerbe P. Understanding the chemical language mediating maize immunity and environmental adaptation. New Phytol. 2024:243(6):2093–2101. 10.1111/nph.20000 39049575

[kiaf410-B208] Zabel RA, Morrell JJ. Chapter eight—chemical changes in wood caused by decay fungi. In: Zabel RA, Morrell JJ, editors. Wood microbiology. 2nd ed. San Diego: Academic Press; 2020. p. 215–244 10.1016/B978-0-12-819465-2.00008-5

[kiaf410-B209] Zeikus JG . Lignin metabolism and the carbon cycle: polymer biosynthesis, biodegradation, and environmental recalcitrance. Adv Microb Ecol. 1981:5:211–243. 10.1007/978-1-4615-8306-6_5

[kiaf410-B210] Zhan A, Lynch JP. Reduced frequency of lateral root branching improves N capture from low-N soils in maize. J Exp Bot. 2015:66(7):2055–2065. 10.1093/jxb/erv007 25680794 PMC4378636

[kiaf410-B211] Zhang X, Izaurralde RC, Arnold JG, Sammons NB, Manowitz DH, Thomson AM, Williams JR. Comment on “modeling miscanthus in the soil and water assessment tool (SWAT) to simulate its water quality effects as a bioenergy crop”. Environ Sci Technol. 2011:45(14):6211–6212. author reply 6213–6214. 10.1021/es201463x 21692538

[kiaf410-B212] Zhang X, Sun H, Bi J, Yang B, Zhang J, Wang C, Zhou S. Estimate greenhouse gas emissions from water-saving and drought-resistance rice paddies by deNitrification-deComposition model. Clean Technol Environ Policy. 2022:24(1):161–171. 10.1007/s10098-021-02094-z

[kiaf410-B213] Zhao Z, Fernie AR, Zhang Y. Engineering nitrogen and carbon fixation for next-generation plants. Curr Opin Plant Biol. 2025:85:102699. 10.1016/j.pbi.2025.102699 40056871

[kiaf410-B214] Zhong V, Archibald BN, Brophy JAN. Transcriptional and post-transcriptional controls for tuning gene expression in plants. Curr Opin Plant Biol. 2023:71:102315. 10.1016/j.pbi.2022.102315 36462457 PMC12061055

[kiaf410-B215] Ziegler J . Report of the special rapporteur on the right to food. Geneva (Switzerland): United Nations Human Rights Council; 2007. https://www.future-agricultures.org/wp-content/uploads/pdf-archive/Victoria Marin Jon Lovett and Joy Clancy.pdf

[kiaf410-B216] Zomer RJ, Bossio DA, Sommer R, Verchot LV. Global sequestration potential of increased organic carbon in cropland soils. Sci Rep. 2017:7(1):15554. 10.1038/s41598-017-15794-8 29138460 PMC5686149

[kiaf410-B217] Zub HW, Arnoult S, Brancourt-Hulmel M. Key traits for biomass production identified in different Miscanthus species at two harvest dates. Biomass Bioenergy. 2011:35(1):637–651. 10.1016/j.biombioe.2010.10.020

