# Supplementary material for: Harnessing plant agriculture to mitigate climate change: A framework to evaluate synthetic biology (and other) interventions
Source: Plant Physiol. 2025 Sep 26;199(3):kiaf410. doi: 10.1093/plphys/kiaf410 (PMC12651577; doi:10.1093/plphys/kiaf410)
Supplement: kiaf410_Supplementary_Data [file kiaf410_supplementary_data.pdf]

Supplementary File: Fermi Analysis

Harnessing Plant Agriculture to Mitigate Climate Change: A Framework to Evaluate Synthetic Biology (and other) Interventions

Vickers, Claudia E.[1,2] & Zerbe, Philipp[3]

[1] School of Biology and Environmental Science, ARC Centre of Excellence in Synthetic Biology, and Centre for Agriculture and the Bioeconomy, Queensland University of Technology, Brisbane, QLD, Australia 4001

[2]BioBuilt Solutions, Brisbane, QLD, Australia 4075

[3] Department of Plant Biology, College of Biological Sciences, University of California Davis, Davis, CA, USA

Contents

Fermi Analyses Methods 3

Fermi Estimations, Interpretation, & Notes 4

Global Cropland CO_2 Fixation 4

Assumptions & Calculations 4

Interpretation & Notes 4

Sporopollenin-Based Carbon Sequestration 5

Assumptions & Calculations: US Maize 5

Interpretation & Notes: US Maize 5

Sporopollenin-Based Carbon Sequestration in *Miscanthus* in Europe 6

Assumptions & Calculations for shoot-targeted accumulation 6

Interpretation & Notes – Sporopollenin in Miscanthus 6

Comparison with maize sporopollenin carbon storage potential 7

Assumptions & Calculations: Additional biomass carbon drawdown 7

Interpretation & Notes – additional carbon drawdown 8

Improved CO_2 fixation in US Maize 8

Assumptions & Calculations: 0.1 Mha deployment area 8

Interpretation & Notes: 0.1 Mha deployment area 9

Assumptions & Calculations for US Field Corn Crop 9

Interpretation & Notes: US Field Corn Crop 10

Decreasing per-hectare yield loss in rice 10

Assumptions & Calculations 10

Interpretation & Notes 11

Forestry Trees with decreased photorespiratory loss 11

Assumptions & Calculations: Poplar 12

Interpretation & Notes: Poplar 13

Assumptions & Calculations: Eucalyptus 13

Interpretation & Notes: Eucalyptus 14

Assumptions & Calculations: Global forestry plantation 14

Interpretation & Notes: Global forestry plantation 15

Biochar-Based Carbon Sequestration 15

Assumptions & Calculations: Australian wheat stover as a feedstock 15

Interpretation & Notes 16

Assumptions & Calculations: land required to draw down 1Gt CO_2e 16

Interpretation & Notes 17
Reforestation (large scale) 18
Assumptions & Calculations 18
Interpretation & Notes 18
Afforestation 19
Assumptions & Calculations 19
Interpretation & Notes 19
Decreasing global rice methane emissions 20
Assumptions & Calculations 20
Interpretation & Notes 20
Converting agricultural residue burning into biochar 21
Assumptions & Calculations 21
Interpretation and Notes 21
Engineering Alternatives to the Haber-Bosch Process 22
Assumptions & Calculations 22
Interpretation & Notes: 23
References 23

Fermi Analyses Methods

The sections below provide details for the assumptions that were made to run Fermi analysis and how decisions were made to settle on the selected numbers, including references from which data was sourced. Interpretations and notes are provided after each Ferm analysis. Generative AI (specifically, ChatGPT) was used to assist in developing the tables and notes. Information provided by ChatGPT was carefully reviewed and cross-checked, especially for references, since generative AI tends to hallucinate (generate fake references), particularly when asked for very specific outcomes. This necessitates cross-checking each reference and confirming both its validity and integrity as a source for the numbers extracted. References and associated commentary, as well as the interpretation and notes sections, were also reviewed and edited to ensure accuracy. When used carefully and with an understanding of its strengths and weaknesses, generative AI can dramatically accelerate this kind of analysis. Some more specialized generative AIs may be better at this kind of analysis.

Fermi Estimations, Interpretation, & Notes

Global Cropland CO_2 Fixation

Assumptions & Calculations

Table S1. Estimated carbon fixation in global croplands based on Gross Primary Production (GPP) and Net Primary Production (NPP). *CO_2 equivalent values were calculated using a standard molar conversion factor (1 Pg C = 3.67 Gt CO_2).*

Parameter	Estimate	Notes & Assumptions
**Global GPP**	157 Pg C/year	Global Gross Primary Production (GPP): 157 Pg C/year (Lai et al., 2024)
**Cropland % GPP**	20 %	Croplands assumed to contribute ~20% of global Gross Primary Production (GPP). **Land Area as a First-Order Proxy:** Croplands cover ~11–13% of the Earth's ice-free land surface (Ramankutty et al., 2008; FAO, 2021). While croplands don't have the highest productivity per unit area (compared to tropical forests, for instance), they are managed intensively and can sustain high seasonal productivity. This justifies using their global share as a first-order approximation. **Satellite-Derived GPP Estimates:** According to data from MODIS and other remote sensing platforms: Zhao & Running (2010) estimated global terrestrial GPP at ~123 Pg C/year, with croplands accounting for 18–20% of this, depending on the year and satellite inputs; Sitch et al. (2015) and other model-based estimates also support croplands contributing between 15–23% of global GPP. **Empirical:** A recent study by Du et al. (2022) used satellite-constrained light-use efficiency models to estimate cropland GPP at values roughly in line with 20% of total terrestrial productivity. This value also aligns with trends reported in the Global Carbon Project and Earth system model outputs.
**Cropland GPP**	31.4 Pg C/year	157 Pg C/year x 0.2
**Cropland NPP**	15.7 Pg C/year	Assuming ~50 % of fixed carbon is lost due to photorespiration (Amthor et al., 2019)
**Cropland GPP (CO_2e)**	115.2 Gt CO_2/year	Converted using 1 Pg C = 3.67 Gt CO_2, based on molar mass ratio.
**Cropland NPP (CO_2e)**	57.6 Gt CO_2/year	Represents potential net sequestration if carbon retained in biomass and soil.

Interpretation & Notes

Croplands contribute significantly to global carbon cycling, fixing an estimated 31.4 Pg C/year via photosynthesis, which translates to ~115 Gt CO_2/year. While most of this carbon is eventually returned to the atmosphere, Net Primary Production (NPP) reflects the portion potentially retained in plant biomass and soils. With NPP estimated at ~15.7 Pg C/year (~57.6 Gt CO_2/year), these values highlight the importance of optimizing cropland management not only for productivity but also for enhancing long-term carbon retention in agricultural systems.

Sporopollenin-Based Carbon Sequestration

Assumptions & Calculations: US Maize

Supplementary Table S2. Assumptions used to estimate the carbon sequestration potential of engineered sporopollenin deposition in maize roots. *The analysis is based on experimental data demonstrating the feasibility of enhancing sporopollenin biosynthesis in maize roots for durable carbon storage (Bathe et al., 2023).*

Assumption	Value Used	Source/Calculation
**Deployment area**	37 Mha	USDA (2025) estimates ~37-38 Mha of harvested maize area in the USA
**Sporopollenin accumulation rate**	0.5 t/ha/year	Bathe et al (2023): Estimated achievable accumulation in engineered maize roots
**Carbon content of sporopollenin**	60%	Based on elemental analysis of sporopollenin (Li et al., 2019)
**Carbon stored per hectare**	0.30 t C/ha/year	0.5 t/ha @ 60 % carbon content
**CO_2 equivalent per hectare**	1.10 t CO_2e/ha/year	0.3 t C/ha/year × 3.67 (universal chemical stoichiometry: 1 tonne of C = 3.67 tonnes of CO_2)
**Total potential mitigation**	40.7 Mt CO_2e/year	1.10 t CO_2e/ha × 37 million ha

Interpretation & Notes: US Maize

- **Novel carbon sequestration mechanism:** Engineering maize roots to biosynthesize and deposit sporopollenin—a highly recalcitrant biopolymer traditionally found in pollen walls—introduces a new pathway for long-term carbon storage within agricultural systems.
- **Exceptional chemical stability:** Sporopollenin is renowned for its resistance to chemical and biological degradation, persisting in soils for extended periods. Its incorporation into root tissues could significantly enhance the durability of sequestered carbon.
- **Integration with existing crops:** This strategy leverages maize, a globally cultivated staple, allowing for potential scalability without necessitating changes in land use or crop selection.
- **Potential for co-benefits:** Beyond carbon storage, sporopollenin deposition may confer additional advantages, such as improved root resilience to environmental stresses and pathogens, though these effects require further investigation.
- **Research and development considerations:** While promising, this approach is in the experimental phase. Key areas for future research include optimizing genetic pathways for sporopollenin synthesis in roots, assessing impacts on plant growth and yield, and evaluating long-term soil interactions.
- **Alignment with sustainable agriculture goals:** By enhancing the carbon sequestration capacity of crops without compromising productivity, this strategy supports broader objectives of sustainable agriculture and climate change mitigation.

Sporopollenin-Based Carbon Sequestration in *Miscanthus* in Europe

Assumptions & Calculations for shoot-targeted accumulation

Supplementary Table S3. Assumptions used to estimate the carbon sequestration potential of engineered sporopollenin deposition in Miscanthus in Europe. *Each assumption is supported by peer-reviewed literature or standard references and includes explanation and calculation logic.*

Assumption	Value used	Source/calculation
**Deployment Area**	10 Mha	Currently only 20,000 ha of miscanthus are planted; the potential area for Miscanthus planting in Europe is estimated to be around 17.5 Mha (Lewandowski et al., 2016). Target of 10 Mha selected for this analysis
**Above-Ground Biomass**	20 t/ha/yr	The estimated above-ground biomass of *Miscanthus* ranges from 15-25 t/ha in the autumn harvest, and 7 to 19 t/ha in the late winter or winter harvest (Zub et al., 2011). Under ideal conditions, yields can reach 23-38 Mg dry biomass ha^{-1} $year^{-1}$, while under poor conditions, they can be 14-15 Mg ha^{-1} $year^{-1}$ (Lewandowski et al., 2016). A conservative number of 20 t/ha/yr was chosen for this study
**Biopolymer Allocation**	10 %	Assumed (same as maize)
**Biopolymer Mass**	2.0 t/ha/yr	10% of total biomass
**Carbon content**	~60%	Carbon content of sporopollenin (Li et al., 2019)
**Carbon Stored**	1.2 t C/ha/yr	60% of biopolymer mass
**CO_2 Equivalent**	4.4 t CO_2e/ha/yr	Carbon × CO_2 conversion factor (3.67; standard chemical stoichiometry)
**Total CO_2 Sequestered**	4.4 Mt CO_2e/yr	Area × CO_2e/ha

Interpretation & Notes – Sporopollenin in Miscanthus

- **Moderate sequestration potential per hectare:** The analysis estimates that converting 10% of *Miscanthus* × *giganteus* shoot biomass to sporopollenin could sequester approximately 4.4 t CO_2e/ha/year. While this is less than some chemical or engineered solutions on a per-hectare basis, it is notable for a biological, plant-based strategy with long-duration storage potential.
- **Durability is a key strength:** Sporopollenin is among the most chemically resistant biopolymers found in nature. Its incorporation into shoot biomass and potential persistence in soil (particularly if incorporated through no-till harvesting or used in durable products) makes this strategy highly attractive for >1,000-year carbon permanence — comparable to biochar or mineralized CO_2.
- **Scalability is constrained but not implausible:** At 10 Mha of deployment, the model predicts sequestration of 44 Mt CO_2e/year, which is meaningful at national or sectoral levels (e.g. equivalent to ~10% of France's annual emissions). However, *Miscanthus* currently covers only ~20,000 ha in Europe. Scaling to 10 Mha would require major shifts in land use, subsidies, infrastructure, and grower adoption.
- **Integration with marginal land use is an opportunity:** Because Miscanthus can thrive on degraded or marginal lands, expansion could avoid direct conflict with food production. Co-benefits such as erosion control, biodiversity support, and soil improvement may strengthen its socio-ecological case.

- **Genetic and biotechnological feasibility remains a key challenge:** Engineering Miscanthus to divert 10% of shoot biomass to sporopollenin — a biopolymer typically restricted to pollen walls — is speculative and unproven at bench scale, let alone at field scale. Engineerability, metabolic burden, developmental regulation, and trait stability all require further investigation.
- **Monitoring and verification considerations:** Long-term carbon storage claims hinge on verifying sporopollenin accumulation and persistence. This may be technically complex in soil or plant residue matrices and would require standardized methodologies for carbon accounting and durability certification.

Comparison with maize sporopollenin carbon storage potential

Once the shoot sporopollenin carbon storage potential is calculated (see worked example in the manuscript proper), it is straightforward to calculate the root and total sporopollenin carbon storage potential. Extrapolating using further assumptions noted in Table 1 (Box 2, manuscript proper) provides the data presented in Supplementary Table S4 below.

Supplementary Table S4. Sporopollenin mass, carbon stored, CO_2 equivalent, and total CO_2 sequestered in different tissues of US maize and European Miscanthus*. These data presume 10 % of biomass as sporopollenin using the total US corn crop (37 Mha) and a potential crop of 10 Mha* Miscanthus. *Assumptions for US maize are from Bathe et al. (2023).*

**Crop**	**U.S. Maize**			**European *Miscanthus***		
**Tissue**	Shoot	Root	Total	Shoot	Root	Total
**Sporopollenin Mass (t/ha/yr)**	1.5	0.13	1.64	2	1	3
**Carbon Stored (t C/ha/yr)**	0.9	0.08	0.98	1.2	0.6	1.8
**CO_2 Equivalent (t CO_2e/ha/year)**	3.3	0.3	3.6	4.4	2.2	6.6
**Total CO_2 Sequestered (Mt CO_2e/year)**	122	11	133	44	22	66

Assumptions & Calculations: Additional biomass carbon drawdown

Supplementary Table S5. Assumptions used to estimate the additional carbon drawdown potential from expanded Miscanthus cultivation. *Excluding engineered sporopollenin. This estimate accounts for net new carbon accumulation resulting from conversion of low-biomass or degraded marginal lands to high-yielding perennial Miscanthus. Assumptions include typical biomass yield, standard carbon content, and the CO_2 equivalence factor. The analysis excludes soil carbon or indirect land use change effects and assumes full biomass retention or stabilization.*

**Assumption**	**Value Used**	**Source/Calculation**
**Deployment area**	10 Mha	Target area for scaled-up Miscanthus deployment (from current ~20,000 ha)
**Above-ground biomass yield**	20 t/ha/year	Typical value for established Miscanthus in temperate climates (Lewandowski et al., 2016)
**Carbon content of biomass**	45%	Standard carbon fraction of dry plant matter (IPCC, 2006)
**Carbon stored per hectare**	9.00 t C/ha/year	20 t/ha/year × 45% carbon content
**CO_2 equivalent per hectare**	33.03 t CO_2e/ha/year	9.0 t C/ha/year × 3.67 conversion factor
**Total potential drawdown**	330.3 Mt CO_2e/year	9.0 t CO_2e/ha/year × 10 million ha

Interpretation & Notes – additional carbon drawdown

- **Substantial per-hectare drawdown potential**: At 33 t CO_2e/ha/year, Miscanthus expansion offers one of the highest biomass-based sequestration rates evaluated
- **Deployment on marginal lands maximizes additionality**: The assumption that new Miscanthus plantings occur on low-productivity or abandoned land allows nearly all biomass to be considered net new carbon drawdown, with minimal displacement or leakage.
- **Requires stable carbon storage downstream**: This estimate presumes harvested biomass enters a stable carbon pool—e.g. biochar, building materials, or soil organic matter—rather than being combusted or decomposed rapidly.
- **Potential co-benefits include soil restoration and erosion control**: Miscanthus improves soil structure, water retention, and can rehabilitate degraded land, particularly in areas prone to erosion or nutrient loss.
- **Scalability depends on economic incentives and land-use policy**: Despite its high yield and low input requirements, Miscanthus adoption has been slow without strong policy support, highlighting the need for subsidies, carbon pricing, or bioproduct markets to drive uptake.

Improved CO_2 fixation in US Maize

This analysis explores improving CO_2 fixation through improving RUBISCO/photosynthesis or increasing the overall efficiency, for example by implementing a synthetic glycolate metabolism pathway (Ort et al., 2015; South et al., 2019; Wu et al., 2019). The US maize crop is used as an example crop species.

Assumptions & Calculations: 0.1 Mha deployment area

Supplementary Table S6. Assumptions used to estimate the carbon sequestration potential improved CO_2 fixation in US maize. *Each assumption is supported by peer-reviewed literature or standard references and includes explanation and calculation logic.*

Assumption	Value Used	Source(s) & Explanation
**Deployment area**	0.1 Mha	A modest-scale deployment scenario appropriate for early commercial phases; represents ~ 0.26% of the total U.S. maize cultivation area
**Yield gain from improved photosynthesis**	+15% biomass increase	Published range for potential for enhancing photosynthetic efficiency: 10–25% (Ort et al., 2015; South et al., 2019; Wu et al., 2019). Conservative estimate of 15% used.
**Baseline biomass yield**	15 t/ha/year	Typical value for maize; supported by USDA (2025) and Liu et al. (2020)
**Additional biomass**	2.25 t/ha/year	Additional biomass from photosynthetic improvement: 15% of 15 t/ha/year.
**Carbon content of biomass**	45%	Standard value for dry plant material per IPCC (2006)
**Carbon stored per hectare**	1.01 t C/ha/year	2.25 t/ha × 0.45.
**CO_2 equivalent per hectare**	3.7 t CO_2e/ha/year	1.01 t C × 3.67 conversion factor
**Total potential sequestration**	0.37 Mt CO_2e/year	3.7 t CO_2e/ha × 100,000 ha.

Interpretation & Notes: 0.1 Mha deployment area

- **Modest contribution at early deployment scale:** At 0.1 Mha (approximately 0.26% of U.S. maize area), improved CO_2 fixation contributes an estimated 0.37 Mt CO_2e/year—modest but measurable at early deployment scale. This is useful for demonstration of impact but not yet substantial in the context of national mitigation targets.
- **Significant scale-up potential:** If deployed across the entire U.S. maize crop (~35–38 Mha), the theoretical drawdown could exceed 130 Mt CO_2e/year, contingent on biomass fate and carbon retention.
- **Trait is yield-positive and stackable:** Improved CO_2 fixation can likely be deployed without yield penalty and is compatible with other engineered traits such as nitrogen-use efficiency, water-use efficiency, or stress resilience.
- **Carbon retention depends on biomass fate:** This estimate assumes the additional carbon is stored in a durable form (e.g. returned to soil, converted to biochar, or used in long-lived products). Rapid decomposition would limit sequestration durability.
- **Soil carbon benefits are not included:** The estimate does not account for changes in root biomass or rhizosphere carbon dynamics, which could add to overall carbon drawdown potential.
- **Key limitations are genetic complexity and regulatory hurdles:** Field validation, trait stability, and regulatory approval pathways remain critical barriers to full-scale deployment.

Assumptions & Calculations for US Field Corn Crop

Supplementary Table S7. Estimated carbon sequestration potential from improved photosynthetic CO_2 fixation in U.S. field corn (maize). *Assumptions are based on literature estimates of biomass gains, standard carbon content, and global stoichiometry. The 100-year drawdown assumes annual incremental biomass accumulation is retained or stabilized and represents the cumulative potential at national scale under sustained deployment. Field corn is assumed to represent 90% of total U.S. corn cultivation.*

Assumption	Value Used	Source(s) & Explanation
**Deployment area (U.S. field corn crop)**	34.3 Mha	94.1 million acres of corn planted in the US (USDA, 2023) × 90 % field corn (assumed) × 0.4047 (conversion factor from million acres to million hectares)
**Yield gain from improved photosynthesis**	+15% biomass increase	Published range for potential for enhancing photosynthetic efficiency: 10–25% (Ort et al., 2015; South et al., 2019; Wu et al., 2019). Conservative estimate of 15% used.
**Baseline biomass yield**	15 t/ha/year	Typical value for maize; supported by USDA (2025) and Liu et al. (2020)
**Additional biomass**	2.25 t/ha/year	Additional biomass from photosynthetic improvement: 15% of 15 t/ha/year.
**Carbon content of biomass**	45%	Standard value for dry plant material per IPCC (2006)
**Carbon stored per hectare**	1.01 t C/ha/year	2.25 t/ha × 0.45.
**CO_2e per hectare**	3.7 t CO_2e/ha/year	1.01 t C × 3.67 conversion factor
**Total potential sequestration**	127.5 Mt CO_2e/year	3.7 t CO_2e/ha × 34.27 million ha.
**100-Year CO_2e per hectare**	372 t CO_2e/ha	3.72 t CO_2e/ha/year × 100 years
**100-Year CO_2e Impact (Gt)**	12.75 Gt CO_2e	372 t CO_2e/ha × 34.27 million ha ÷ 1e9

Interpretation & Notes: US Field Corn Crop

- **Adjusted Deployment Area**: The difference in impact between the **pilot-scale deployment (0.1 Mha)** and **full-scale national deployment (34.27 Mha)** is profound: the total **annual impact increases by ~343x**, and the **100-year cumulative drawdown increases from ~0.04 Gt to 12.75 Gt CO_2e**.
- This dramatic difference illustrates the **nonlinear climate relevance** of deploying traits not only efficiently but **widely across staple crops** like maize.
- **Per-Hectare Efficiency Remains Constant:** Importantly, per-hectare carbon drawdown remains identical between the two scenarios—this reinforces the notion that trait efficiency is scalable, and that expanding deployment is a key driver of total impact.
- **Effect of mass-scale deployment:** National-scale estimate demonstrates that, when deployed across **major monoculture systems like U.S. field corn**, synthetic biology interventions **can contribute gigatonne-scale impact** under sustained carbon retention conditions.
- **Caveats:** These estimates assume: complete and uniform trait expression across all field corn hectares; that the additional biomass is stabilized in soil or long-lived products; no rebound effect (e.g. increased yield leading to higher emissions elsewhere)
- **Assumed stabilization:** These estimates assume that the additional biomass carbon is effectively stabilized in the soil or used in durable materials, and that the improved traits are consistently expressed across the entire field corn cultivation area.

Decreasing per-hectare yield loss in rice

Assumptions & Calculations

Supplementary Table S8. Assumptions used to estimate the carbon mitigation potential from increasing per-hectare yield of rice. *Yield gain assumptions are based on literature describing engineered disease resistance and stress resilience traits in rice. The analysis assumes the additional biomass results in net carbon gain, stored in durable form (e.g. retained in soil, biomass products, or replacing lower-yield systems).*

Assumption	Value Used	Source/Calculation
**Deployment area**	15 Mha	Global area of paddy rice under cultivation is ~160 Mha (FAO, 2024). A deployment of ~15 Mha (~10%) is used here to model near-term adoption of engineered yield-enhancing traits in key regions.
**Baseline above-ground biomass**	15 t/ha/year	Above-ground rice biomass yield is quite variable, at ~14–20 t/ha/year (Katsura et al., 2010; Tu et al., 2022; Zheng et al., 2023; Swain et al., 2024). Value of 15 t/ha used as conservative average.
**Relative yield gain**	+20%	Based on a range of engineered and edited trait examples (e.g. BBM1/MiMe asexual propagation system, Pik-1 for resistance to *Magnaporthe*, WRKY36 knockout, etc.) that confer 10–30% yield or loss avoidance gains (Khanday et al., 2019; Liu et al., 2025; Rim et al., 2025)
**Additional biomass**	2 t/ha/year	Additional biomass from improvement: 20% of 10 t/ha/year
**Carbon content of biomass**	45%	Standard carbon content for plant dry matter (IPCC, 2006).

**Carbon stored per hectare**	1.35 t C/ha/year	2.0 t/ha × 0.45
**CO_2 equivalent per hectare**	4.95 t CO_2e/ha/year	0.9 t C/ha/year × 3.67
**Total potential mitigation**	74.3 Mt CO_2e/year	3.3 t CO_2e/ha/year × 15,000,000 ha

Interpretation & Notes

- **Moderate mitigation potential at conservative scale:** At 15 Mha—less than 10% of global rice-growing area—yield-enhancing traits could mitigate approximately **74.3 Mt CO_2e/year**, assuming additional biomass results in net carbon retention.
- **Achievable gains with emerging technologies:** The analysis assumes a 20% biomass increase, supported by published results from traits that improve photosynthetic efficiency, pest resistance, or stress tolerance, e.g. WRKY36 knockout, BBM1/MiMe asexual seed system, Pik-1 disease resistance
- **Carbon benefit depends on fate of biomass:** For carbon mitigation to occur, the additional biomass must avoid rapid re-emission—for example by increasing soil organic matter, displacing lower-yielding systems, or entering durable material pools.
- **High food system compatibility:** Enhancing per-hectare productivity improves land-use efficiency without requiring crop switching, supporting both **climate mitigation and food security** in land-constrained regions.
- **Trait deployment challenges remain:** Genetic complexity, trait stacking, regulatory approval, and equitable access in smallholder systems are all critical to widespread adoption.
- **Indirect mitigation pathways are plausible but not included:** This analysis focuses only on the direct carbon value of additional biomass. Indirect benefits—such as land sparing, reduced methane from lower-flooded fields, or input-use efficiency—may further increase net impact.

Forestry Trees with decreased photorespiratory loss

For these Fermi analyses, we are exploring the additional CO2 drawdown potential of forestry species if the current plantations were replaced with faster-growing biomass-enhanced species. The Fermi question addressed here is: "How much more CO_2 is fixed by engineered forestry species with enhanced biomass over 100 years compared to conventional forestry species, assuming constant rotation and full plantation turnover?" These analyses are a little more complicated, as we have chosen to incorporate two key dynamics: (a) progressive replacement of existing plantations with engineered lines, and (b) age-dependent growth over (current) forestry rotation cycle. We assume no expansion of total plantation area, but that each newly planted tree includes the enhanced trait. To capture realistic deployment and carbon uptake dynamics, we model year-by-year carbon drawdown over a 100-year period. For each year y, the annual CO_2e gain (Mt) is calculated as the sum of contributions from plantation cohorts aged between 1 and the harvest year, each scaled by its age-adjusted carbon gain. The gain for age *a* is approximated as a linear ramp from 15% at year 1 to 100% at harvest year. The total annual CO_2 gain is:

$$CO_2e_y = \sum_{a=1}^{\min(y,R)} \left(\frac{A}{R}\right) \cdot f(a) \cdot G$$

where:
A = total plantation area (ha)
R = rotation length (years)
$f(a)$ = fractional maturity at age a
G = additional mature CO_2e gain (t CO_2e/ha/year)

Equation 1

The cumulative CO_2e gain (Gt) is the year-on-year sum of annual gains, converted from Mt to Gt. Under these assumptions, full deployment is reached by the harvest year of the first planting.

Assumptions & Calculations: Poplar

The Fermi question addressed here is: "How much more CO_2 is fixed by engineered poplar (~40% enhanced biomass) over 100 years compared to conventional poplar, assuming constant rotation and full plantation turnover?"

Supplementary Table S9. Assumptions used to estimate carbon drawdown potential from genetically engineered poplar with reduced photorespiration. *The table includes two scenarios: current global wood poplar plantations and hypothetical full replacement with enhanced poplar. Values are derived from peer-reviewed literature, company data, and standard chemical conversion factors.*

Assumption	Value Used	Notes / References
**Deployment area**	1.6 Mha	Estimated current global area of poplar plantations (FAO, 2020)
**Rotation length**	30 years	Assumes complete turnover of poplar stock over ~30 years
**Annual replanting**	$1/30^{th}$ total area	~53,000 ha/year assuming uniform replacement
**Above-ground biomass yield**	15 t/ha/year	Average mature yield of poplar (IPCC, 2006; Living_Carbon, 2022)
**Relative gain from engineered trait**	+40%	Tao et al. (2023): top-performing line had 35–53% more biomass; mid-range 40% used
**Additional biomass from enhancement**	6 t/ha/year	15 t/ha × 40% = 6 t/ha
**Carbon content of biomass**	45%	Standard dry biomass value (IPCC, 2006)
**CO_2 conversion factor**	3.67	Molecular conversion from C to CO_2
**Carbon stored per hectare**	2.7 t C/ha/year	6 t × 45%
**Age-dependent growth**	Linear from 15% to 100%	First-year trees assumed to sequester 15% of full rate, sequestration scales linearly to 100 % by Year 30
**CO_2e/ha/year, year 1 (gain)**	1.49 t CO_2e/ha/year	Reflects the early-stage growth phase and small area planted
**CO_2e/ha/year at full maturity (gain)**	9.91 t CO_2e/ha/year	2.7 t C × 3.67. This assumes 40% biomass increase over baseline of 15 t/ha/year
**Year 1 impact (gain)**	2.38 Mt CO_2e	Young trees, partial growth
**Year 30 impact (gain)**	15.85 Mt CO_2e	Full deployment reached
**100-year cumulative impact**	0.27 Gt CO_2e	Sum of year-by-year CO_2 gain. Includes age-structured ramp-up based on plantation replacement model (Equation 1)

Interpretation & Notes: Poplar

- **Incremental drawdown reflects deployment and growth timing**: This analysis estimates the *net additional CO_2 fixed* by engineered poplar with ~40% increased biomass productivity over conventional poplar, assuming gradual plantation turnover over 30 years. The model accounts for both the **annual replanting cycle** (1/30th of the global 1.6 Mha plantation) and the **age-dependent CO_2 gain** of growing trees.
- **Age-adjusted carbon gain moderates early-year impact**: Because trees grow slowly in their early years, newly planted cohorts contribute a smaller share of annual CO_2 fixation. A linear ramp was applied, with 15% of mature productivity in year 1 increasing to 100% by year 30. This reduces near-term annual CO_2e gain estimates compared to maturity-based projections.
- **No expansion of land use is assumed**: The analysis holds the total poplar plantation area constant and considers only the effect of substituting conventional trees with engineered lines. This makes the drawdown estimate conservative and focused on biological efficiency gains.
- **Cumulative 100-year gain is modest but scalable**: Under these assumptions, the additional drawdown over 100 years is estimated at **0.27 Gt CO_2e**, representing a meaningful but modest contribution to climate mitigation when applied at current global plantation scale. Larger impacts would require **land use expansion**, **integration into wood products**, or **deployment of similar traits in other species**.
- **Biomass fate not included**: The estimate reflects **gross additional carbon fixation only**. Downstream effects such as decomposition, bioenergy use, or long-term wood storage are not included but could significantly affect net sequestration outcomes.

Assumptions & Calculations: Eucalyptus

The Fermi question addressed here is: "How much more CO_2 is fixed by engineered Eucalyptus (*E. grandis* + *E. globulus*, ~20% enhanced biomass) over 100 years compared to conventional eucalyptus, assuming constant rotation and full plantation turnover?"

Supplementary Table S10. Assumptions and calculations for 100-year additional CO_2 sequestration from engineered Eucalyptus plantations. *The analysis models plantation turnover on a 15-year cycle with age-adjusted carbon accumulation. Results reflect only the incremental drawdown due to genetic enhancement.*

Assumption	Value Used	Source / Calculation Notes
**Deployment Area**	20 Mha	FAO (2020) Global Forest Resources Assessment
**Rotation Length**	15 years	Typical eucalyptus harvest age (Grattapaglia and Kirst, 2008)
**Annual Replanting**	1/15 of area	20 Mha / 15 = ~1.33 Mha/year
**Mature Biomass Yield**	18 t/ha/year	Average mature yield
**Relative Gain From Trait**	+20%	More conservative engineering assumption
**Additional Biomass**	3.60 t/ha/year	18 × 0.20
**Carbon Content**	45%	Standard for woody biomass (IPCC, 2006)
**Carbon Stored**	1.62 t C/ha/year	3.60 × 0.45
**Age-Based Growth Curve**	Linear (15% to 100%)	Used for year-wise ramp-up (Equation 1)
**CO_2e/ha/year (year 1) gain**	0.89 t CO_2e/ha/year	Reflects the early-stage growth phase and small area planted

**CO_2e/ha/year (full maturity)**	5.95 t CO_2e/ha	1.62 × 3.67
**Year 1 Gain**	17.84 Mt CO_2e	New trees at lowest productivity
**Year 15 Gain**	118.91 Mt CO_2e	Full deployment of engineered trait achieved
**100-Year Cumulative Gain**	6.22 Gt CO_2e	Summed annual gains. Includes age-structured ramp-up based on plantation replacement model

Interpretation & Notes: Eucalyptus

- **This analysis estimates the incremental CO_2 drawdown** resulting from deploying genetically engineered eucalyptus trees with a +20% increase in biomass productivity relative to conventional varieties.
- **A total deployment area of 20 Mha** is assumed, reflecting current global eucalyptus plantation estimates (primarily in tropical and subtropical regions).
- **A 15-year rotation period** is applied, consistent with global eucalyptus forestry practices. Carbon accumulation is modelled using an age-dependent linear ramp from 15% to 100% of mature productivity over the rotation cycle.
- **Year 1 gains are modest (~0.89 t CO_2e/ha/year)** due to the immaturity of newly planted trees, while full maturity gains reach ~5.95 t CO_2e/ha/year.
- **The estimated cumulative 100-year gain** from trait-based biomass enhancement is ~6.22 Gt CO_2e, assuming complete and sustained replacement of conventional trees without expanding land area.
- **This scenario isolates the genetic contribution to biomass** and does not account for other ecosystem or product-level C dynamics (e.g., harvested wood fate, soil C).
- As with prior models, no land area expansion is assumed

Assumptions & Calculations: Global forestry plantation

Supplementary Table S11. Assumptions and calculations for 100-year additional CO_2 sequestration if the entire global forestry plantation was engineered. *The analysis models plantation turnover on an average 15-year cycle with age-adjusted carbon accumulation. Results reflect only the incremental drawdown due to genetic enhancement.*

Assumption	Value Used	Source / Calculation Notes
**Deployment Area**	131 Mha	FAO (2020) Global Forest Resources Assessment
**Rotation Length**	15 years	Weighted estimate based on species composition
**Annual Replanting**	8.73 Mha/year	131 Mha / 15
**Mature Biomass Yield**	15 t/ha/year	Average mature yield (IPCC, 2006; FAO, 2020)
**Relative Gain From Trait**	+20%	More conservative enhancement estimate
**Additional Biomass**	3.00 t/ha/year	15 × 0.20
**Carbon Content**	45%	Standard for woody biomass (IPCC, 2006)
**Carbon Stored**	1.35 t C/ha/year	3.00 × 0.45
**Age-Based Growth Curve**	Linear (15% to 100%)	Used for year-wise ramp-up (Equation 1)
**CO_2e/ha/year (year 1) gain**	0.74 t CO_2e/ha/year	Reflects the early-stage growth phase and small area planted
**CO_2e/ha/year (full maturity) gain**	4.95 t CO_2e/ha	1.62 × 3.67
**Year 1 Gain**	6.49 Mt CO_2e	New trees at lowest productivity
**Year 15 Gain**	373.2 Mt CO_2e	Full deployment of engineered trait achieved
**100-Year Cumulative Gain**	33.97 Gt CO_2e	Summed annual gains. Includes age-structured ramp-up based on plantation replacement model

Interpretation & Notes: Global forestry plantation

- **This scenario models the global impact** of engineering all plantation forest species (131 Mha) for a +20% biomass gain, using a harmonized approach across diverse species and geographies.
- **A 15-year average rotation period** is used to reflect the global mean across faster-growing tropical hardwoods and slower-growing temperate conifers.
- **Baseline biomass productivity is set at 15 t/ha/year**, a weighted average derived from FAO and IPCC data. Engineered gains are modeled using the same age-dependent linear ramp approach as above.
- **Year 1 CO_2e gain is modest (~0.74 t CO_2e/ha/year)**, increasing to ~4.95 t CO_2e/ha/year at full maturity, due to young stand age in initial deployment.
- **Over 100 years**, as the global plantation estate is completely replaced by engineered high-biomass trees, the **cumulative additional drawdown is ~33.97 Gt CO_2e**.
- This analysis focuses strictly on **above-ground productivity gains due to genetic enhancement** and assumes no expansion in forested area. It does not account for harvested biomass use or substitution effects.

Biochar-Based Carbon Sequestration

Assumptions & Calculations: Australian wheat stover as a feedstock

Supplementary Table S12. Biochar-Based CO_2e Drawdown from Australian Wheat Stover. *Estimated carbon dioxide sequestration potential from sustainable harvesting of wheat stover in Australia for biochar production. Assumptions are based on average wheat stubble yields, standard pyrolysis conversion efficiencies, and long-term stable carbon fractions. Carbon content and stability assumptions follow IPCC guidelines and established biochar life cycle assessments. CO_2e estimates assume that biochar is applied to soil and not subsequently re-emitted to the atmosphere.*

Assumption	Value Used	Source/Calculation
**Australian wheat cultivation area**	12 Mha	Based on ABS and ABARES estimates of wheat area in Australia (2018–2023)
**Sustainable stover yield per hectare**	3.5 t/ha/year	Agricultural residues in Australia are ~80 Mt per year (Crawford et al., 2016). Wheat is 22 % of this; the average wheat yield in Australia is approximately 2.61 tonnes per hectare (USDA, 2025). The harvest index for wheat (ratio of grain yield to total above-ground biomass) is typically around 0.4 (Flower et al., 2020). Total above-ground biomass per hectare can be calculated as: Total biomass = Grain yield / HI = 2.61 t/ha / 0.4 = 6.525 t/ha. Residue yield per hectare is then: Residue yield = Total biomass - Grain yield = 6.525 t/ha - 2.61 t/ha = 3.915 t/ha. Considering variations in grain yields and harvest indices across different regions and seasons, the sustainable residue yield (amount that can be harvested consistently without negatively impacting soil health or crop productivity) can range approximately from 3.5 to 5.5 t/ha/year (Verheijen et al., 2010; Crawford et al., 2016)
**Biochar conversion efficiency**	30%	Typical pyrolysis efficiency for crop residues
**Carbon content of biochar**	80%	Standard carbon content of biochar (Lehmann and Joseph, 2024)
**Stable carbon fraction**	70%	Fraction of biochar carbon considered stable over long-term (Woolf et al., 2010)

**Stable carbon per hectare**	0.59 t C/ha/year	3.5 t/ha × 30% × 80% × 70%
**CO_2e sequestered per hectare**	2.16 t CO_2e/ha/year	Stable carbon per ha × 3.67
**Total CO_2e drawdown (Mt/year)**	25.90 Mt CO_2e/year	CO_2e per ha × 12 million ha

Interpretation & Notes

- **Wheat stover represents a nationally scalable biomass feedstock for biochar production:** in Australia, with an estimated potential of nearly 26 Mt CO_2e/year based on current cultivation area and conservative residue removal rates
- **Biomass sustainability was prioritized** in yield estimates (3.5 t/ha/year), ensuring soil organic matter is not excessively depleted.
- Relies on **fraction of biochar carbon considered stable** over long-term (Woolf et al., 2010)
- **Drawdown efficiency is lower than with energy/biomass crops** like Miscanthus, but the large land base and existing crop infrastructure provide a strong foundation
- **The CO_2e impact depends critically on biochar stability** and whether it is applied to soil with minimal further disturbance or oxidation
- This estimate **excludes emissions associated with collection, transport, and pyrolysis**, which would need to be accounted for in a full life cycle assessment.

Assumptions & Calculations: land required to draw down 1Gt CO_2e

Supplementary Table S13. Assumptions used to estimate the carbon drawdown potential of biochar-based sequestration. *Assumptions include area planted (Mha), above-ground biomass yield (t/ha/year), carbon conversion efficiency to biochar, and estimated carbon permanence. The fraction of biomass converted to stable carbon is based on conservative pyrolysis efficiency and life cycle estimates. The table also includes standard carbon content of biomass and the CO_2 equivalence conversion factor. Total CO_2e sequestration potential is calculated based on the product of sequestered carbon per hectare and total area, assuming stable incorporation of biochar in soils. Estimation of the land area required to sequester 1 Gt CO_2e/year using biochar in Australia*

Assumption		Source/calculation
**Biomass yield**	~10 t/ha/yr	High biomass crops such as switchgrass, giant miscanthus: 14 – 40 t/ha/yr (McLaughlin and Adams Kszos, 2005; Lewandowski et al., 2016). Crops with biomass side streams (e.g. broadacre cereal crops such as corn, wheat, etc.): estimate is 3.5 – 4.1 t/ha/yr *sustainable* yield - meaning it can be harvested consistently without negatively impacting soil health or crop productivity (Verheijen et al., 2010; Crawford et al., 2016; Cifuentes García et al., 2024). An average order-of-magnitude assumption considering various sources is ~10 t/ha/yr. Other useful references consulted: (Sohi et al., 2010; Lehmann and Joseph, 2024).
**Biochar yield**	~30%	(Lehmann and Joseph, 2024; Schmidt et al., 2024)
**Carbon content of biochar**	~80%	European Biochar Certificate technical guidelines and life cycle assessments (Schmidt et al., 2024)
**Carbon permanence**	~70%	(Verheijen et al., 2010; Woolf et al., 2010)
**CO_2 conversion factor**	3.67	Universal chemical stoichiometry: 1 tonne of C = 3.67 tonnes of CO_2
**Land required to remove 1 Gt CO_2e/year**	162 Mha	Approximately 162 million hectares would be required to sequester 1 Gt CO_2e/year.

Interpretation & Notes

- This estimate assumes **average global biomass yields** for feasible land—e.g., temperate, semi-arid, or degraded lands producing **~10 t/ha/year** through crop residues, grasses, or moderate-yield perennial species. For **high-yield systems** (e.g. irrigated Miscanthus or sugarcane at 20–30 t/ha/year), **less land would be required; f**or **lower-yield or residue-limited systems**, more land would be needed.
- **Estimates reflect sustainable biomass removal rates:** The biomass yields used in this analysis are based on sustainable harvest assumptions—i.e., the portion of crop residues or biomass that can be removed without degrading soil organic carbon, nutrient balance, or long-term productivity.
- **Gigaton-scale potential at realistic yields:** Based on conservative assumptions (~10 t/ha/year biomass, 30% biochar yield, 70% carbon permanence), approximately **162 million hectares** would be required to sequester **1 Gt CO_2e/year**.
- **Supported by multiple feedstock sources:** The analysis draws from a wide range of crops and residues.
- **Carbon conversion and permanence assumptions are robust:** Biochar is assumed to retain ~80% carbon, with ~70% of that carbon remaining stable over centuries to millennia, in line with empirical life cycle analyses (Verheijen et al., 2010; Woolf et al., 2010; Schmidt et al., 2024)
- **Favorable techno-economic characteristics:** Biochar production can be low-cost (~$30–100/t$CO_2$e) when integrated with existing waste streams or energy systems, and may generate co-products such as heat or syngas depending on pyrolysis configuration (Lehmann and Joseph, 2024)
- **Multiple soil-related co-benefits:** Biochar improves soil **water retention**, **nutrient holding capacity**, and **microbial activity**, potentially enhancing yield and reducing fertilizer needs—especially in degraded or sandy soils (Sohi et al., 2010; Schmidt et al., 2024)
- **Scalability depends on biomass supply and deployment logistics:** While technically scalable, implementation at the GtCO_2e level depends on regional biomass availability, transport costs, pyrolysis infrastructure, and carbon accounting frameworks. Australia's agricultural residues alone contribute ~80 Mt/year (Crawford et al., 2016), suggesting national-scale feasibility.
- **Small-scale, distributed manufacturing** is probably required to minimise transport costs and facilitate return of benefits to the source farm (soil carbon, wood oil, etc.)
- **Soil and ecological risks are low but must be monitored:** Properly produced biochar (low in contaminants) is environmentally safe, but quality control, feedstock traceability, and ecological impact assessments are critical, particularly at scale.

Reforestation (large scale)

Assumptions & Calculations

Supplementary Table S14. Assumptions used to estimate the carbon drawdown potential of reforestation. *Carbon accumulation rates are based on large-scale forest recovery datasets and IPCC Tier 1 assumptions. The estimate focuses on the early decades of regrowth and assumes stable forest recovery and low disturbance rates.*

Assumption	Value Used	Source/Calculation
**Deployment area**	150 Mha	Assumes restoration of ~150 million hectares of degraded forestland, consistent with the "high-ambition" scenario from Bastin et al. (Bastin et al., 2019) and initiatives like the Bonn Challenge.
**Annual biomass carbon sequestration rate**	3.0 t C/ha/year	Based on meta-analyses of tropical and temperate reforestation projects (Pan et al., 2011; Griscom et al., 2017), forest regrowth typically sequesters 2–4 t C/ha/year over the first few decades. A central value of 3.0 t C/ha/year is used here.
**Carbon content of biomass**	N/A	Rate already expressed in t C/ha/year.
**CO_2 equivalent per hectare**	11.0 t CO_2e/ha/year	3.0 t C/ha/year × 3.67 (conversion factor from C to CO_2e).
**Total potential drawdown**	1,650 Mt CO_2e/year	11.0 t CO_2e/ha/year × 150 Mha.

Interpretation & Notes

- **Large-scale carbon drawdown opportunity:** Reforesting 150 Mha of degraded land has the potential to sequester **1.65 Gt CO_2e/year**, making it one of the highest-impact biological carbon removal strategies currently available.
- **Carbon removal rate based on early-stage regrowth:** The estimate assumes a sequestration rate of **~3 t C/ha/year**, typical of young to mid-aged regenerating forests in tropical and temperate zones. Sequestration tends to be higher in early decades and tapers as forest biomass approaches saturation.
- **Long-duration carbon storage with minimal re-emission risk:** If protected from disturbance, reforestation can store carbon in biomass and soils for **centuries to millennia**, aligning with the highest durability tiers (>1,000 years) in carbon offset frameworks.
- **Supported by existing global policy frameworks:** This opportunity aligns with the goals of the Bonn Challenge, UN Decade on Ecosystem Restoration, and IPCC AR6 land use mitigation scenarios, many of which include large-scale reforestation as a core mitigation pillar.
- **Land availability and permanence are key challenges:** While 150 Mha is feasible globally on degraded and low-productivity lands, competing demands for food, fuel, and conservation may limit implementation. Fire, pests, or political instability could also threaten permanence.

- **Biodiversity and hydrology co-benefits:** Reforestation enhances biodiversity, watershed regulation, and soil health, especially when native or mixed-species plantings are prioritized over monocultures.
- **Excludes indirect impacts and leakage effects:** This estimate assumes all land converted remains forested and does not cause displacement of agriculture or wood harvesting elsewhere—critical factors in project-level analysis.

Afforestation

Assumptions & Calculations

Supplementary Table S15. Assumptions used to estimate the carbon drawdown potential of afforestation*. Values are based on representative sequestration rates for temperate and subtropical afforestation programs. The estimate assumes long-term forest establishment and stable biomass accumulation over decades.*

Assumption	Value Used	Source/Calculation
**Deployment area**	100 Mha	High-confidence scenario based on available land from (Bastin et al., 2019); FAO Global Forest Resources Assessment (FAO, 2020)
**Annual biomass carbon sequestration rate**	2.5 t C/ha/year	Average long-term rate across temperate and subtropical afforestation projects (IPCC, 2006; Griscom et al., 2017)
**Carbon content of biomass**	N/A (rate already in t C)	Carbon rate already expressed as t C/ha/year, no adjustment needed
**CO_2 equivalent per hectare**	9.2 t CO_2e/ha/year	2.5 t C/ha/year × 3.67 (conversion factor from C to CO_2e)
**Total potential mitigation**	920 Mt CO_2e/year	9.2 t CO_2e/ha/year × 100 Mha

Interpretation & Notes

- **High global potential for carbon drawdown:** With 100 Mha of land afforested, this strategy could sequester approximately **920 Mt CO_2e/year**, assuming moderate growth rates and stable forest cover.
- **Realistic carbon accumulation rate for new forests:** The rate of **2.5 t C/ha/year** is based on long-term averages across temperate and subtropical afforestation sites. Rates may be higher in tropical systems and lower in cold or dry regions.
- **Long-duration storage with proper forest management:** If forests are maintained, afforestation provides highly durable carbon sinks, storing biomass carbon and building soil organic matter over multi-decadal timescales.
- **Land availability and socio-ecological trade-offs:** While afforestation can occur on degraded or marginal land, scaling to 100 Mha must be balanced against biodiversity, food production, and Indigenous land rights considerations.
- **Climate and ecosystem co-benefits:** Properly implemented afforestation can restore wildlife habitat, regulate hydrology, reduce erosion, and buffer microclimates—especially if diverse native species are planted.
- **Excludes soil carbon and indirect effects:** This estimate focuses on above-ground biomass accumulation and does not account for additional soil carbon sequestration or potential indirect land use impacts

Decreasing global rice methane emissions

Assumptions & Calculations

Supplementary Table S16. Estimated CO_2e mitigation potential from reducing methane emissions in global paddy rice cultivation. *A 30% reduction in CH_4 using agronomic strategies could mitigate ~86 Mt CO_2e/year, or 0.54 t CO_2e/ha/year. Over 100 years, this totals 8.6 Gt CO_2e avoided. Assumes a CH_4 GWP100 of 27.*

Assumption	Value Used	Justification / Source
**Global rice cultivation area**	160 Mha	(FAO, 2024)
**Methane emissions from rice**	~10.6 Tg CH_4/year	(Linquist et al., 2012; IPCC, 2022)
**CH_4 Global Warming Potential (GWP100)**	27	(IPCC, 2022)
**Baseline CO_2e from rice CH_4**	286 Mt CO_2e/year	10.6 Tg × 27
**Emissions reduction scenario**	30% reduction	30 % is a realistic, conservative, evidence-based average of potential CH_4 emission reductions that can be achieved using currently available mitigation practices in rice production, including alternate wetting and drying (AWD), mid-season drainage and intermittent irrigation, improved fertilizer and organic matter management (Richards et al., 2015; IPCC, 2022). We will assume that engineered traits can deliver a similar decrease in emissions
**Reduced CH_4 emissions**	3.18 Tg CH_4/year	30% of 10.6 Tg
**CO_2e avoided (annual)**	86 Mt CO_2e/year	3.18 Tg × 27
**CO_2e avoided (per hectare)**	0.54 t CO_2e/ha/year	86 Mt ÷ 160 Mha
**100-year cumulative impact**	8.6 Gt CO_2e	86 Mt/year × 100 years

Interpretation & Notes

- **100-year mitigation potential:** At constant implementation, the 100-year cumulative mitigation potential from a 30% reduction in rice CH_4 emissions is ~8.6 Gt CO_2e.
- **Large-scale impact from small per-hectare effect:** Though the mitigation per hectare is modest, the large global rice area amplifies the impact.
- **Immediate deployability:** This mitigation strategy requires no new biotech or land, and can be rapidly scaled via training and incentives.
- **Durability & permanence:** Methane reductions avoid near-term warming, offering high time-value climate benefit. Benefits cease if practices are not maintained.
- **Potential trade-offs:** Some mitigation practices (e.g. alternate wetting/drying) can elevate N_2O emissions if not properly managed

Converting agricultural residue burning into biochar

Assumptions & Calculations

This analysis estimates the potential CO_2e emissions reduction if all agricultural residues currently burned worldwide were instead converted into biochar and applied to soil. Most studies indicate that well-managed pyrolysis emits negligible methane compared to open biomass burning, where CH_4 emissions can be significant.

Supplementary Table S17. Estimated climate mitigation potential of converting agricultural residue burning to biochar production. *This Fermi analysis demonstrates the large-scale emissions reduction potential of substituting open burning practices with stable carbon storage via biochar, including reduced methane emissions and long-term soil sequestration.*

Parameter	Estimate	Notes & Sources
**Global agricultural residues burned annually**	~500 Mt dry biomass	Moderate estimate; literature range from 300–900 Mt/year (Streets et al., 2003; Yevich and Logan, 2003; FAO, 2017)
**Biochar yield (from pyrolysis)**	30%	Standard for slow pyrolysis of crop residues
**Carbon content of biochar**	80%	Typical value for lignocellulosic biochar
**Stable carbon fraction**	70%	Long-term persistence in soil (IPCC, 2006; Woolf et al., 2010)
**Stable carbon per tonne of burned biomass**	0.168 t C	1 t biomass × 0.3 × 0.8 × 0.7
**Total stable carbon from 500 Mt biomass**	84 Mt C/year	500 × 0.168
**CO_2e drawdown (annual)**	308 Mt CO_2e/year	84 × 3.67
**100-year cumulative CO_2e drawdown**	30.8 Gt CO_2e	Assuming full deployment & 100-year persistence

Interpretation and Notes

Pros:

- Significantly reduces CH_4 and black carbon emissions from open burning
- Enables long-term, stable carbon sequestration in soils
- Enhances soil health, water retention, and nutrient cycling
- Compatible with circular bioeconomy and distributed rural deployment

Cons:

- Requires investment in pyrolysis infrastructure and logistics
- Biochar properties and soil impacts can be variable and context-specific
- Life cycle emissions from transport and processing must be monitored

Replacing open burning of ~500 Mt/year of agricultural residues with biochar production could reduce emissions by ~308 Mt CO_2e/year, with a cumulative climate benefit of ~31 Gt CO_2e over 100 years—largely from avoided methane and stabilized carbon.

Engineering Alternatives to the Haber-Bosch Process

Assumptions & Calculations

Supplementary Table S18. Estimated mitigation potential from replacing the Haber–Bosch process and fertilizer-associated emissions with synthetic biology-derived nitrogen fixation or other low-emission alternatives. *Assumes 30% emissions reduction sustained over 100 years, totaling ~130 Gt CO_2e avoided.*

Assumption	Value Used	Justification / Source
**Haber–Bosch share of global CO_2 emissions**	1.8%	(Royal_Society, 2020; IPCC, 2022)
**Global CO_2e emissions (2019 baseline)**	59 Gt CO_2e/year	UNEP Emissions Gap Report (UNEP, 2023)
**Fertilizer-derived CH_4 and N_2O share (indirect)**	~3.2 Gt CO_2e/year	(IPCC, 2022)
**Combined fertilizer-related emissions (direct + indirect)**	~4.3 Gt CO_2e/year	1.8% HB + ~2.5 Gt indirect soil N_2O/CH_4
**Feasible reduction from synthetic bio-based replacement**	30% emissions reduction	Engineered nitrogen fixation may reduce synthetic N use by ~30%. **SynBio potential in maize and cereals:** Van Gelder et al. (2023) performed a Fermi-style analysis of a *maize aerial root mucilage trait*, suggesting it could reduce N fertilizer requirements by ~10% without yield penalty. Burén and Rubio (2017) and Guo et al. (2023) discussed engineering symbiotic or associative nitrogen fixation in cereals, with target nitrogen replacement goals ranging from 20% to 50%, depending on strategy maturity and biological feasibility**. Industry benchmarks:** Companies like Pivot Bio and Kula Bio claim N fertilizer replacement in the range of 20–40% in initial product pipelines under field conditions for cereal crops. Pilot deployment of diazotroph-based inoculants in the U.S. corn belt has shown potential for 10–30% reductions in conventional N application. **Comparative precedent**: In sustainable intensification literature, interventions that optimize nitrogen use efficiency or biological inputs often achieve 15–35% emissions reductions when applied at scale (IPCC, 2022; Smith, 2023). **30 % is a mid-range of realistic expectations** for nitrogen replacement by 2050 across a range of crop types and geographies. It reflects partial substitution: full replacement is biologically and agronomically difficult in the short term. It allows for mixed-system scenarios where synthetic and biological nitrogen coexist and evolve in parallel.
**Annual avoided emissions**	~1.3 Gt CO_2e/year	30% of 4.3 Gt
**Avoided emissions per hectare (global arable land)**	~0.46 t CO_2e/ha/year	~1.3 Gt ÷ 2.8 Bn ha est. global cropland
**100-year cumulative mitigation impact**	130 Gt CO_2e	Assumes sustained 1.3 Gt/year reduction

Interpretation & Notes:

- **Substantial long-term potential:** Fertilizer-related emissions exceed 4 Gt CO_2e/year. Replacing Haber–Bosch with biological alternatives could meaningfully reduce this.
- **Plausible deployment:** A 30% reduction aligns with intermediate adoption of engineered diazotrophs and other biotechnologies.
- **Large 100-year benefit:** Sustained deployment would yield ~130 Gt CO_2e mitigation.
- **Risk factors:** Deployment, regulatory acceptance, and ecological safety are all challenges.
- **Dual benefit:** Reductions stem from both manufacturing (fossil hydrogen) and downstream emissions (e.g., N_2O).

References

Amthor JS, Bar-Even A, Hanson AD, Millar AH, Stitt M, Sweetlove LJ, Tyerman SD (2019) Engineering Strategies to Boost Crop Productivity by Cutting Respiratory Carbon Loss. The Plant Cell **31:** 297-314

Bastin J-F, Finegold Y, Garcia C, Mollicone D, Rezende M, Routh D, Zohner CM, Crowther TW (2019) The global tree restoration potential. Science **365:** 76-79

Bathe U, Leong BJ, Van Gelder K, Barbier GG, Henry CS, Amthor JS, Hanson AD (2023) Respiratory energy demands and scope for demand expansion and destruction. Plant Physiol **191:** 2093-2103

Burén S, Rubio LM (2017) State of the art in eukaryotic nitrogenase engineering. FEMS Microbiology Letters **365**

Cifuentes García R, Galán G, Martín M (2024) Multiscale analysis for the valorization of biomass via pellets production towards energy security. Journal of Cleaner Production **461:** 142663

Crawford DF, O'Connor MH, Jovanovic T, Herr A, Raison RJ, O'Connell DA, Baynes T (2016) A spatial assessment of potential biomass for bioenergy in Australia in 2010, and possible expansion by 2030 and 2050. GCB Bioenergy **8:** 707-722

Du Z, Zhang X, Zhang Y (2022) Estimating Global Cropland GPP Using a Light Use Efficiency Model Constrained by Satellite Observations. Remote Sensing **14:** 1722

FAO (2017) The Future of Food and Agriculture – Trends and Challenges. Food and Agriculture Organization of the United Nations, https://www.fao.org/3/i6583e/i6583e.pdf

FAO (2020) Global Forest Resources Assessment 2020. Food and Agriculture Organization of the United Nations, https://www.fao.org/forest-resources-assessment/2020/en/

FAO (2021) FAOSTAT: Land Use Statistics. Food and Agriculture Organization of the United Nations, FaAOotU Nations. https://www.fao.org/faostat/en/#data/RL

FAO (2024) FAOSTAT: Cropland Area Dataset. *In* FaAOotU Nations, ed, https://www.fao.org/faostat/en/

FAO (2024) FAOSTAT: Crops and Livestock Products - Rice Area Harvested. *In* FaAOotU Nations, ed, https://www.fao.org/faostat/en/#data/QCL

Flower K, Dang Y, Ward P (2020) Advances in crop residue management. *In* J Pratley, J Kirkegaard, eds, Australian Agriculture in 2020: From Conservation to Automation, https://espace.library.uq.edu.au/view/UQ:ab5456f. Agronomy Australia, Wagga Wagga, NSW, Australia, pp 137-149

Grattapaglia D, Kirst M (2008) Eucalyptus applied genomics: from gene sequences to breeding tools. New Phytologist **179:** 911–929

Griscom BW, Adams J, Ellis PW, Houghton RA, Lomax G, Miteva DA, Schlesinger WH, Shoch D, Siikamäki JV, Smith P, Woodbury P, Zganjar C, Blackman A, Campari J, Conant RT, Delgado C, Elias P, Gopalakrishna T, Hamsik MR, Herrero M, Kiesecker J, Landis E, Laestadius L, Leavitt SM, Minnemeyer S, Polasky S, Potapov P, Putz FE, Sanderman J, Silvius M, Wollenberg E, Fargione J (2017) Natural climate solutions. Proceedings of the National Academy of Sciences **114:** 11645-11650

Guo K, Yang J, Yu N, Luo L, Wang E (2023) Biological nitrogen fixation in cereal crops: Progress, strategies, and perspectives. Plant Communications **4:** 100499

IPCC (2006) 2006 IPCC Guidelines for National Greenhouse Gas Inventories, Volume 4: Agriculture, Forestry and Other Land Use, https://www.ipcc-nggip.iges.or.jp/public/2006gl/vol4.html. Institute for Global Environmental Strategies (IGES), Hayama, Japan

IPCC (2006) Guidelines for National Greenhouse Gas Inventories: Agriculture, Forestry and Other Land Use (AFOLU). Intergovernmental Panel on Climate Change, https://www.ipcc-nggip.iges.or.jp/public/2006gl/vol4.html

IPCC (2022) Climate Change 2022: Mitigation of Climate Change – Working Group III Contribution to the IPCC Sixth Assessment Report. Cambridge University Press, https://www.ipcc.ch/report/ar6/wg3/

IPCC (2022) Climate Change 2022: Mitigation of Climate Change. Contribution of Working Group III to the Sixth Assessment Report. Intergovernmental Panel on Climate Change, CU Press. https://www.ipcc.ch/report/ar6/wg3/

Katsura K, Okami M, Mizunuma H, Kato Y (2010) Radiation use efficiency, N accumulation and biomass production of high-yielding rice in aerobic culture. Field Crops Research **117:** 81-89

Khanday I, Skinner D, Yang B, Mercier R, Sundaresan V (2019) A male-expressed rice embryogenic trigger redirected for asexual propagation through seeds. Nature **565:** 91-95

Lai J, Kooijmans LMJ, Sun W, Lombardozzi D, Campbell JE, Gu L, Luo Y, Kuai L, Sun Y (2024) Terrestrial photosynthesis inferred from plant carbonyl sulfide uptake. Nature **634:** 855-861

Lehmann J, Joseph S (2024) Biochar for environmental management: science, technology and implementation, Ed 3. Taylor & Francis, London

Lewandowski I, Clifton-Brown J, Trindade LM, van der Linden GC, Schwarz KU, Muller-Samann K, Anisimov A, Chen CL, Dolstra O, Donnison IS, Farrar K, Fonteyne S, Harding G, Hastings A, Huxley LM, Iqbal Y, Khokhlov N, Kiesel A, Lootens P, Meyer H, Mos M, Muylle H, Nunn C, Ozguven M, Roldan-Ruiz I, Schule H, Tarakanov I, van der Weijde T, Wagner M, Xi Q, Kalinina O (2016) Progress on Optimizing Miscanthus Biomass Production for the European Bioeconomy: Results of the EU FP7 Project OPTIMISC. Front Plant Sci **7:** 1620

Li F-S, Phyo P, Jacobowitz J, Hong M, Weng J-K (2019) The molecular structure of plant sporopollenin. Nature Plants **5:** 41-46

Linquist BA, van Groenigen KJ, Adviento-Borbe MA, Pittelkow CM, van Kessel C (2012) An agronomic assessment of greenhouse gas emissions from major cereal crops. Global Change Biology **18:** 194–209

Liu D, He J, Li Q, Zhang X, Wang Y, Sun Q, Wang W, Zhang M, Wang Y, Xu H, Fang L, Jiang L, Liu S, Chen L, Tian Y, Liu X, Wang R, Zhang Z, Chern M, Dong X, Wang H, Liu Y, Ronald PC, Wan J (2025) A WRKY transcription factor confers broad-spectrum resistance to biotic stresses and yield stability in rice. Proceedings of the National Academy of Sciences **122:** e2411164122

Liu W, Hou P, Liu G, Yang Y, Guo X, Ming B, Xie R, Wang K, Liu Y, Li S (2020) Contribution of total dry matter and harvest index to maize grain yield—A multisource data analysis. Food and Energy Security **9:** e256

Living_Carbon (2022) Photosynthesis Enhanced Trees Grow Faster and Capture More Carbon. https://www.livingcarbon.com/post/photosynthesis-enhanced-trees-grow-faster-and-capture-more-carbon. Accessed 22/03/2025

McLaughlin SB, Adams Kszos L (2005) Development of switchgrass (Panicum virgatum) as a bioenergy feedstock in the United States. Biomass and Bioenergy **28:** 515-535

Ort DR, Merchant SS, Alric J, Barkan A, Blankenship RE, Bock R, Croce R, Hanson MR, Hibberd JM, Long SP, Moore TA, Moroney J, Niyogi KK, Parry MAJ, Peralta-Yahya PP, Prince RC, Redding KE, Spalding MH, van Wijk KJ, Vermaas WFJ, von Caemmerer S, Weber APM, Yeates TO, Yuan JS, Zhu XG (2015) Redesigning photosynthesis to sustainably meet global food and bioenergy demand. Proceedings of the National Academy of Sciences **112:** 8529-8536

Pan Y, Birdsey RA, Fang J, Houghton R, Kauppi PE, Kurz WA, Phillips OL, Shvidenko A, Lewis SL, Canadell JG, Ciais P, Jackson RB, Pacala SW, McGuire AD, Piao S, Rautiainen A, Sitch S, Hayes D (2011) A Large and Persistent Carbon Sink in the World's Forests. Science **333:** 988-993

Ramankutty N, Evan AT, Monfreda C, Foley JA (2008) Farming the planet: 1. Geographic distribution of global agricultural lands in the year 2000. Global Biogeochemical Cycles **22**

Richards M, Sander BO, Bruun TB (2015) Alternate wetting and drying in irrigated rice: Implementation guidance for policy makers and investors. CGIAR Research Program on Climate Change, Agriculture and Food Security (CCAFS), https://cgspace.cgiar.org/handle/10568/68831

Rim EY, Garrett OD, Howard AJ, Shim Y, Li Y, Van Dyke JE, Packer RC, Ho N, Jain R, Stewart V, Dinesh-Kumar SP, Notwell JH, Ronald PC (2025) Directed Evolution of a Plant Immune Receptor for Broad Spectrum Effector Recognition. bioRxiv 10.1101/2024.09.30.614878**:** 2024.2009.2030.614878

Royal_Society (2020) Ammonia: Zero-Carbon Fertiliser, Fuel and Energy Store: Policy Briefing. Royal Society

Schmidt HP, Bucheli T, Kammann C, Glaser B, Abiven S, Leifeld J, Soja G, Hagemann N (2024) European Biochar Certificate - Guidelines for a Sustainable Production of Biochar 2021-2024. Carbon Standards International (CSI), Frick, Switzerland. Frick, Switzerland

Sitch S, Friedlingstein P, Gruber N, Jones SD, Murray-Tortarolo G, Ahlström A, Doney SC, Graven H, Heinze C, Huntingford C, Levis S, Levy PE, Lomas M, Poulter B, Viovy N, Zaehle S, Zeng N, Arneth A, Bonan G, Bopp L, Canadell JG, Chevallier F, Ciais P, Ellis R, Gloor M, Peylin P, Piao SL, Le Quéré C, Smith B, Zhu Z, Myneni R (2015) Recent trends and drivers of regional sources and sinks of carbon dioxide. Biogeosciences **12:** 653-679

Smith A (2023) Methane mitigation: methods to reduce emissions, potential, and costs. *In* UNEPUaU-cCaCA Coalition, ed, Global Methane Assessment: Benefits and Costs of Mitigating Methane Emissions, https://www.globalmethanepledge.org/sites/default/files/documents/2023-11/2021_Global-Methane_Assessment_full_0.pdf. United Nations Environment Programme, Nairobi

Sohi SP, Krull E, Lopez-Capel E, Bol R (2010) A Review of Biochar and Its Use and Function in Soil. *In* Advances in Agronomy, Vol 105. Academic Press, pp 47-82

South PF, Cavanagh AP, Liu HW, Ort DR (2019) Synthetic glycolate metabolism pathways stimulate crop growth and productivity in the field. Science **363:** eaat9077

Streets DG, Yarber KF, Woo J-H, Carmichael GR (2003) Biomass burning in Asia: Annual and seasonal estimates and atmospheric emissions. Global Biogeochemical Cycles **17**

Swain KC, Singha C, Pradhan B (2024) Estimating Total Rice Biomass and Crop Yield at Field Scale Using PlanetScope Imagery Through Hybrid Machine Learning Models. Earth Systems and Environment **8:** 1713-1731

Tao Y, Chiu L-W, Hoyle JW, Dewhirst RA, Richey C, Rasmussen K, Du J, Mellor P, Kuiper J, Tucker D, Crites A, Orr GA, Heckert MJ, Godinez-Vidal D, Orozco-Cardenas ML, Hall ME (2023) Enhanced Photosynthetic Efficiency for Increased Carbon Assimilation and Woody Biomass Production in Engineered Hybrid Poplar. Forests **14:** 827

Tu D, Wu W, Xi M, Zhou Y, Xu Y, Chen J, Shao C, Zhang Y, Zhao Q (2022) Effect of Temperature and Radiation on Indica Rice Yield and Quality in Middle Rice Cropping System. Plants (Basel) **11**

UNEP (2023) Emissions Gap Report 2023: Broken Record – Temperatures Hit New Highs, Yet World Fails to Cut Emissions (Again). United Nations Environment Programme, https://www.unep.org/resources/emissions-gap-report-2023

USDA (2023) USDA National Agricultural Statistics Service - Charts and Maps - A to Z - Corn. *In* NASS United States Department of Agriculture, ed, https://www.nass.usda.gov/Charts_and_Maps/A_to_Z/in-corn.php

USDA (2025) Australia Wheat Area, Yield and Production. *In* FAS United States Department of Agriculture, ed, https://ipad.fas.usda.gov/countrysummary/Default.aspx?crop=Wheat&id=AS. International Production Assessment Division

USDA (2025) United States Corn Area, Yield and Production Summary. *In* FAS United States Department of Agriculture, ed, https://ipad.fas.usda.gov/countrysummary/Default.aspx?id=US&crop=Corn

Van Gelder K, Oliveira-Filho ER, Messina CD, Venado RE, Wilker J, Rajasekar S, Ane JM, Amthor JS, Hanson AD (2023) Running the numbers on plant synthetic biology solutions to global problems. Plant Sci **335:** 111815

Verheijen F, Jeffery S, Bastos AC, van der Velde M, Diafas I (2010) Biochar Application to Soils: A Critical Scientific Review of Effects on Soil Properties, Processes and Functions. Office for the Official Publications of the European Communities, Luxembourg. https://publications.jrc.ec.europa.eu/repository/handle/JRC57810

Woolf D, Amonette JE, Street-Perrott FA, Lehmann J, Joseph S (2010) Sustainable biochar to mitigate global climate change. Nat Commun **1:** 56

Wu A, Hammer GL, Doherty A, von Caemmerer S, Farquhar GD (2019) Quantifying impacts of enhancing photosynthesis on crop yield. Nat Plants **5:** 380-388

Yevich R, Logan JA (2003) An assessment of biofuel use and burning of agricultural waste in the developing world. Global Biogeochemical Cycles **17**

Zhao M, Running SW (2010) Drought-Induced Reduction in Global Terrestrial Net Primary Production from 2000 Through 2009. Science **329:** 940-943

Zheng B, Wang J, Wu S, Wu H, Xie Z, Wan W (2023) Spatio-temporal patterns and driving mechanisms of rice biomass during the growth period in China since 2000. Ecological Indicators **153:** 110389

Zub HW, Arnoult S, Brancourt-Hulmel M (2011) Key traits for biomass production identified in different Miscanthus species at two harvest dates. Biomass and Bioenergy **35:** 637-651
